# Supplementary material for: Fish predators control outbreaks of Crown-of-Thorns Starfish
Source: Nat Commun. 2021 Dec 8;12:6986. doi: 10.1038/s41467-021-26786-8 (PMC8654818; doi:10.1038/s41467-021-26786-8)
Supplement: Supplementary file 1 — Supplementary Information [file 41467_2021_26786_MOESM1_ESM.pdf]

Supplementary Information

Fish predators control outbreaks of Crown-of-Thorns Starfish

Frederieke J. Kroon[1,*], Diego R. Barneche[2,3] & Michael J. Emslie[1]

[1]Australian Institute of Marine Science, Townsville, Qld 4810, Australia

[2]Australian Institute of Marine Science, Crawley, WA 6009, Australia

[3]Oceans Institute, The University of Western Australia, Crawley, WA 6009, Australia

*email: f.kroon@aims.gov.au

Supplementary Methods

Supplementary Method 1. The Great Barrier Reef Marine Park and population outbreaks of Crown-of-Thorns Starfish

The Great Barrier Reef

The GBR is the largest coral reef system in the world and extends for 2,300 km along Australia's north-eastern coast (all information from[1, 2] unless otherwise noted). It contains a variety of tropical marine ecosystems including almost 3 000 individual coral reefs covering an area of ~20,000 km^2, ~43,000 km^2 of seagrass meadows, ~25,600 km^2 of shoals, extensive mangrove forests and approximately 1,050 islands. The 346,000 km^2 GBR Region was established in 1975, with the 344,400 km^2 GBR Marine Park declared in sections from 1979 to 2001 and amalgamated into one section in 2004. In 1981, the GBR was inscribed as the 348,000 km^2 GBR World Heritage Area by UNESCO. Since establishment of the GBR Region in 1975, the GBR has been managed as a multiple-use area by the Australian Federal and Queensland State Governments. The main uses are commercial marine tourism, commercial and recreational fishing, recreation, research and educational activities, and traditional use, as well as shipping, defence activities, and ports. These multiple uses have been managed under various zoning plans for the GBR Marine Park, outlining activities allowed, prohibited, or requiring a permit in the seven different zones (Supplementary Table 1).

Spatial zoning and marine reserves in the Great Barrier Reef Marine Park

Spatial zoning and marine reserves are critical components of the adaptive management of the GBR Marine Park (all information from[3], unless otherwise noted). Following the implementation of initial zoning schemes from 1981 to 1992, a new zoning plan was implemented in 2004. This resulted in an increase in areas zoned as Marine National Park, i.e. unfished reefs where extractive use is prohibited, from 4.6% to 33.3% of the Marine Park

(Supplementary Table 1), while simultaneously improving the representation of habitat types in these no-take zones. The other two zones covering relatively large areas of the GBR Marine Park are General Use (33.8%) and Habitat Protection (28.2%). The different uses and activities that are allowed within a zone generally become more restrictive from General Use and Habitat Protection Zones to Marine National Park and Preservation Zones. For example, fishing and other harvest activities with the exception of trawling are permitted in Habitat Protection zones; limited fishing (excluding gill netting and trawling) is allowed in Conservation Park zones; and extractive use is prohibited in Marine National Park zones. Of importance to our study is that the 2004 zoning of individual reefs was not affected by their history of CoTS population outbreaks[4], nor was the spatial zoning configuration designed with consideration of any influence on CoTS population outbreaks. In our statistical analyses, we classified a reef's zoning status as 'fished' (General Use, Habitat Protection and Conservation Park zones) or 'unfished' (Marine National Park, Preservation and Scientific Research zones), based on permitted fisheries uses and activities (Supplementary Table 1).

Crown-of-Thorns Starfish population outbreaks on the Great Barrier Reef

Since the 1960s, four CoTS population outbreaks have been recorded on the GBR with all four following a similar pattern of initiation and spread[5, 6]. The first and second outbreaks were reported from Green Island (16.8°S) and nearby reefs in 1962[7, 8] and in 1979[9], respectively. The first stages of the third and fourth outbreaks were detected on mid-shelf reefs between Lizard Island (14.7°S) and Cairns (16.9°S) in 1993–1995[10] and in 2010[5], respectively. These first reports of high CoTS densities in the north-central GBR suggest that outbreaks initiate between Lizard Island and Cairns (i.e. the 'initiation box')[11]. This was recently corroborated by spatiotemporal modelling[6] with high connectivity between reefs in this region potentially exacerbating CoTS outbreaks[12]. Following initiation, the spread of each outbreak progressed in a south-easterly, and likely north-westerly direction, at a rate of

about 60 km per year[6, 13, 14]. The south-easterly spread is consistent with southward dispersal of CoTS larvae spawned at the outbreak front[11, 15], while north-westerly spread is less clear due to fewer surveys having been conducted in the northern GBR. In the southern GBR, outbreaks appear to die off on mid-shelf reefs in the Pompeys' section (21.0° - 22.0° S) approximately 15 years after initiation[5, 6, 11]. The apparent presence of consistent and chronic outbreaks on offshore reefs in the Swain section (22.0°S)[4, 13] suggests that their dynamics are largely independent of the four outbreaks reported since the 1960s, and may also be exacerbated by high connectivity[12].

Life cycle of Crown-of-Thorns Starfish

The life cycle of *Acanthaster* spp. comprises a relatively short planktonic stage (i.e. days to weeks) and a longer settled stage (i.e. years) (all information from[5] unless otherwise noted). The planktonic stage starts with the release of gametes, with estimates of phenomenal oocyte (>100 million eggs) and sperm (1.1×10^{13} sperm) production for larger individual female and male starfish[16]. Detection of larval CoTS in waters of the GBR indicate that spawning generally occurs from November to February[17]. The larval phase (0.5 to 1.5 mm long) typically lasts 11 days with dispersal occurring over large areas including 100 km south of reefs with current outbreaks[15]. Larvae settlement onto reef habitat and subsequent metamorphosis into a benthic juvenile starfish (0.5 mm diameter) takes place over a period of two days. Juvenile CoTS (1–10 mm diameter) will feed on crustose coralline algae before a permanent shift to corallivory. This herbivorous stage generally lasts six months although can extend for 6.5 years in the laboratory[18]. The corallivorous juvenile and sub-adult stages (10–200 mm diameter) last approximately two years, after which CoTS sexually mature into adults (200–350 mm diameter). Larger CoTS (>350 mm in diameter) generally decline and cease gametogenesis after 5+ years. Across both planktonic and settled stages, CoTS are

likely exposed to predation by a variety of coral reef organisms including coral reef fishes[19, 20].

Fisheries in the GBR Marine Park

Both commercial and recreational fishing are allowed in the GBR Marine Park, with different fisheries uses and activities allowed within each of the seven zones (Supplementary Table 1). In line with our hypotheses, the two fisheries operating in the GBR Marine Park that are of particular interest are the Coral Reef Fin Fish Fishery and the Marine Aquarium Fish Fishery[21], with the former comprising line fisheries across the commercial, recreational (including charters) and Indigenous sectors[22]. The Coral Reef Fin Fish Fishery dates back to at least the 1950s[3, 23, 24], with the commercial line fishery primarily targeting coral trout (*Plectropomus* spp. and *Variola* spp.) and redthroat emperor (*Lethrinus miniatus*), with another 20 species targeted including emperors (Lethrinidae), tropical snappers (Lutjanidae), and rockcods (Serranidae) (Supplementary Data 1)[25]. From 2010-11 to 2018-19, the mean total catch and gross value product of this fishery was 1,375 t (±78.6 S.D.) and AUS$29M (±1.7 S.D.), with coral trout and redthroat emperor comprising 57% (±2.4 S.D.) and 14% (±2.6 S.D.) of the total catch, respectively[26]. Similarly, recreational fishers commonly catch coral trout together with several emperor and tropical snapper species[27], however, the retained catch estimates for these species for the recreational charter (Supplementary Data 1), and the recreational and Indigenous sectors (Supplementary Data 2) are less reliable. Notwithstanding, the take of rockcods, emperors, and tropical snappers by recreational (non-charter) fisheries is estimated to have been of similar magnitude to that of the commercial line fisheries for decades[23, 27, 28, 29, 30, 31]. The Marine Aquarium Fish Fishery dates back to the 1970s[32] and targets over 600 different coral reef fish species (Supplementary Data 3)[33]. Between 1998 and 2008, this fishery collected between 130,000 and 260,000 coral reef fish

annually[33] including Pomacentridae (damselfish), Tetraodontidae (pufferfish) and Ballistidae (triggerfish)[34].

Supplementary Method 2. The effects of coral reef fish biomass removal on CoTS densities.

We employed a Bayesian hierarchical approach with mean CoTS density across tows (individuals / minute) at grid site i and year $t + x$ (with $x \in \{1 - 6\}$), $\bar{Y}_{i,[t+x]}$, as a function of mean coral cover across tows, $\bar{C}_{i,[t+x]}$ at year $t + x$, and fish biomass removal, $\bar{B}_{i,t}$, (kg) at year t by assuming that $\bar{Y}_{i,[t+x]}$ follows a hurdle-gamma distribution with a log probability density function, ψ:

$$\psi\left(\bar{Y}_{i,[t+x]} \middle| \alpha, \alpha e^{-\eta_{i,t,x}}, \lambda_{i,t,x}\right) =$$

$$= \begin{cases} \ln\left(\mathrm{Bern}\left(1 \middle| \mathrm{logit}^{-1}\left(\lambda_{i,t,x}\right)\right)\right), & \bar{Y}_{i,[t+x]} = 0 \\ \ln\left(\mathrm{Bern}\left(0 \middle| \mathrm{logit}^{-1}\left(\lambda_{i,t,x}\right)\right)\right) + \ln\left(\mathrm{G}\left(\bar{Y}_{i,[t+x]} \middle| \alpha, \alpha e^{-\eta_{i,t,x}}\right)\right), & \bar{Y}_{i,[t+x]} > 0 \end{cases}$$

$$\lambda_{i,t,x} = \theta_0 + \theta_1 \bar{C}_{i,[t+x]}$$

$$\eta_{i,t,x} = \beta_0 + \beta_1 \bar{C}_{i,[t+x]} + \beta_2 \bar{B}_{i,t} + \zeta_i$$

$$\zeta_i = \delta_i \sigma_\zeta$$

$$\alpha \sim \Gamma(2, 0.5);\ \theta_0 \sim \mathcal{L}(0, 1);\ \theta_1 \sim \mathcal{N}(0, 5);\ \beta_{\{0-2\}} \sim \mathcal{N}(0, 5);$$

$$\delta_i \sim \mathcal{N}(0, 1);\ \sigma_\zeta \sim \Gamma(2, 0.5)$$

where Bern and G are respectively the probability mass and probability density functions of the Bernoulli and Gamma distributions. $\mathrm{logit}^{-1}\left(\lambda_{i,t,x}\right)$ is the hurdle probability of an outcome being zero, which is determined by the linear parameters $\theta_{\{0-1\}}$. The Gamma distribution is parametrised in terms of the shape α and inverse scale $\alpha e^{-\eta_{i,t,x}}$, which is in turn determined by the log linear prediction $\eta_{i,t,x}$ and its linear parameters $\beta_{\{0-2\}}$. i is a vector comprising levels of grid site IDs (total of 42–135 sites depending on the coral reef fish

group, t and x) which in turn compose a hierarchical vector ζ of same length representing grid site-level deviations from $\eta_{i,t,x}$; δ_i is the vector of standardised hierarchical effect and σ_ζ represents the among-grid site standard deviation. The prior sampling distributions are the Gaussian ($\mathcal{N}$(location, scale)), Gamma (Γ(shape, inverse scale)) and Logistic ($\mathcal{L}$(location, scale)).

Hurdle-gamma models were fitted in R using the package *brms* version 2.14.4 [35]. The posterior distributions of model parameters were estimated using Hamiltonian Monte Carlo (HMC) methods by constructing four chains of 5,000 draws each, whereby chains were initiated at distinct points drawn at random. Half of these draws were used as a warm-up, so a total of 10,000 draws were retained to estimate posterior distributions (i.e. 4 × (5,000 - 2,500) = 10,000). We used an adaptation step of 0.99, and a maximum tree depth of 20. All four independent chains reached convergence, i.e. the Gelman-Rubin statistic [36], $\hat{R}$, was 1. We used Bayesian R^2 [37] for the hurdle-gamma models in order to estimate the amount of variation explained by the model (Supplementary Figs. 2 and 3; Supplementary Table 3). Posterior predictive checks, comparisons between prior and posteriors distributions, posterior distribution of model parameters and chain mixing trace-plots can be found in Supplementary Figs. 4–40.

We also ran a post-hoc power analysis to test whether the model and chosen distribution were able to recover the data and parameters originally estimated from our data. In other words, whether the model could be considered a good true data processes generator. To do so, we first drew at random 100 sets of posterior predictions of CoTS density (using the function "posterior_predict" in *brms*), then re-ran the original model for each one of these sets and retained 1,000 draws per model to generate posterior distributions. We then overlaid the original posterior distributions of model parameters (i.e., 10,000 draws as described above) on top of the combination of all posterior distributions across all 100 runs (i.e., 100 ×

1,000 = 100,000 per parameter per model). We demonstrate that the posterior distribution of both originally reported and simulated parameters largely agree (Supplementary Figs. 5–40).

Supplementary Method 3. The effects of zoning on coral reef fish biomass, density, and size.

To determine whether zoning influences coral reef fish density (individuals 1,000 m^{-2}), mean population total body length (cm) and standing biomass (kg 1,000 m^{-2}), we used coral reef fish observations from 840 transects conducted on 56 reefs (each encompassing three sites, with five belt transects (250 x 4 m) per site) along the length of the GBR Marine Park every two years between 2006 and 2020. Each of the three responses was analysed for the following six fish groups, in accordance with the biomass removal analysis described above (Supplementary Method 2). Sites are nested in reefs, and each reef is embedded within a pair of reefs, one being fished, and another unfished. We employed a Bayesian hierarchical approach with mean site-level response (fish density, biomass, or length) at site s, reef pair p, reef r and year t, $\bar{Y}_{s,p,r,t}$, as a function of site-level reef zoning status (fished vs. unfished), $F_{s,r}$, at year t. In the case of fish length, we assume that $\bar{Y}_{s,p,r,t}$ follows a gamma distribution, G, with shape α and inverse scale $\alpha e^{-\eta_{s,p,r,t}}$:

$$\bar{Y}_{s,p,r,t} \sim \mathrm{G}(\alpha, \alpha e^{-\eta_{s,p,r,t}})$$

$$\eta_{s,p,r,t} = \beta_0 + \beta_1 F_{s,r} + \zeta_s + \zeta_p + \zeta_r + \zeta_t$$

$$\zeta_s = \delta_s \sigma_{\zeta,s};\ \zeta_p = \delta_p \sigma_{\zeta,p};\ \zeta_r = \delta_r \sigma_{\zeta,r};\ \zeta_t = \delta_t \sigma_{\zeta,t}$$

$$\alpha \sim \Gamma(1,0.1);\ \beta_{\{0-1\}} \sim \mathcal{N}(0,5);$$

$$\delta_{\{s,p,r,t\}} \sim \mathcal{N}(0,1);\ \sigma_{\zeta,\{s,p,r,t\}} \sim \Gamma(1,0.1),$$

where $\eta_{s,p,r,t}$ is the log linear prediction of site-level mean population length; $F_{s,r}$ is a dummy vector indicating whether a site is unfished (0) or fished (1); thus, β_0 corresponds to the value of $\eta_{s,p,r,t}$ in reefs that are closed to fishing, whereas β_1 is the difference in $\eta_{s,p,r,t}$ between fished and unfished reefs; s, p, r and t are vectors respectively comprising levels of sites, pairs, reefs and year (n = 168, 28, 56, 8) which in turn compose hierarchical vectors ζ of same length representing deviations from $\eta_{s,p,r,t}$; δ are vectors of standardised hierarchical effect and σ_ζ are the standard deviations.

In the case of fish density and standing biomass, we assume that $\bar{Y}_{s,p,r,t}$ follows a hurdle-gamma distribution with a log probability density function, ψ:

$$\psi\left(\bar{Y}_{s,p,r,t}|\alpha, \alpha e^{-\eta_{s,p,r,t}}, \lambda_{s,r}\right) =$$

$$= \begin{cases} \ln\left(\text{Bern}\left(1|\text{logit}^{-1}(\lambda_{s,r})\right)\right), & \bar{Y}_{s,p,r,t} = 0 \\ \ln\left(\text{Bern}\left(0|\text{logit}^{-1}(\lambda_{s,r})\right)\right) + \ln\left(\text{G}\left(\bar{Y}_{s,p,r,t}|\alpha, \alpha e^{-\eta_{s,p,r,t}}\right)\right), & \bar{Y}_{s,p,r,t} > 0 \end{cases}$$

$$\lambda_{s,r} = \theta_0 + \theta_1 F_{s,r}$$

$$\eta_{s,p,r,t} = \beta_0 + \beta_1 F_{s,r} + \zeta_s + \zeta_p + \zeta_r + \zeta_t$$

$$\zeta_s = \delta_s \sigma_{\zeta,s};\ \zeta_p = \delta_p \sigma_{\zeta,p};\ \zeta_r = \delta_r \sigma_{\zeta,r};\ \zeta_t = \delta_t \sigma_{\zeta,t}$$

$$\alpha \sim \Gamma(1, 0.1);\ \theta_0 \sim \mathcal{L}(0,1);\ \theta_1 \sim \mathcal{N}(0,5);\ \beta_{\{0-1\}} \sim \mathcal{N}(0,5);$$

$$\delta_{\{s,p,r,t\}} \sim \mathcal{N}(0,1);\ \sigma_{\zeta,\{s,p,r,t\}} \sim \Gamma(1,0.1).$$

Distribution and fitting specifications for the gamma and hurdle-gamma models follow the same as the one described in Supplementary Method 2, and linear prediction as well as random effects are the same as above for the gamma model (site-level mean population length). Parameter estimates as well as hypothesis testing are presented in Supplementary Table 6. Posterior predictive checks, comparisons between prior and posteriors distributions,

posterior distribution of model parameters and chain mixing trace-plots can be found in Supplementary Figs. 41–60.

Supplementary Method 4. The effects of reef zoning and coral cover on CoTS density.

We employed a Bayesian hierarchical approach to model CoTS density per i^{th} tow (individuals / 2 minutes), Y_i, following a negative binomial distribution, NB, with log-location η_i and inverse overdispersion control φ:

$$Y_i \sim \mathrm{NB}(e^{\eta_i}, \varphi)$$
$$\eta_i = \beta_0 + \beta_1 F_i + \beta_2 C_i + \zeta_j$$
$$\zeta_j = \delta_j \sigma_\zeta$$
$$\varphi \sim \Gamma(1, 0.1);\ \beta_{\{0-2\}} \sim \mathcal{N}(0, 5);$$
$$\delta_j \sim \mathcal{N}(0, 1);\ \sigma_\zeta \sim \Gamma(1, 0.1),$$

where F_i is a dummy vector indicating whether a reef is unfished (0) or fished (1); thus, β_0 corresponds to the value of η_i in reefs that are closed to fishing, whereas β_1 is the difference in η_i between fished and unfished reefs, both when manta-tow level coral cover (proportion), C_i, is zero; β_2 is the rate of change in η_i with coral cover; j is a vector comprising levels of reef-year combinations (total of 3,358 combinations) which in turn compose a hierarchical vector ζ of same length representing reef-year-level deviations from η_i; δ_j is the vector of standardised hierarchical effect and σ_ζ represents the among reef-year standard deviation.

Fitting specifications for the negative binomial model follow the same as the one described in Supplementary Method 2, apart from the maximum tree depth which was 15. A posterior predictive check together with the Bayesian R^2 [37] can be found in Supplementary Fig. 61. Comparisons between prior and posteriors distributions, posterior distribution of model parameters and chain mixing trace-plots can be found in Supplementary Fig. 62. We also employed the R package DHARMa[38] to demonstrate that the data was not zero inflated. The

test is done by contrasting the observed number of zeroes in the data against the posterior distribution of expected zeroes given the negative binomial distribution and parameters (Supplementary Fig. 63). Different from the previous models described in sections 2 and 3, we did not standardise CoTS density to the unit minute because here the data is on the scale of individual manta tow observations (2 minutes), whereas in the previous sections it was an aggregate across manta tows within a reef or grid.

Supplementary Figures

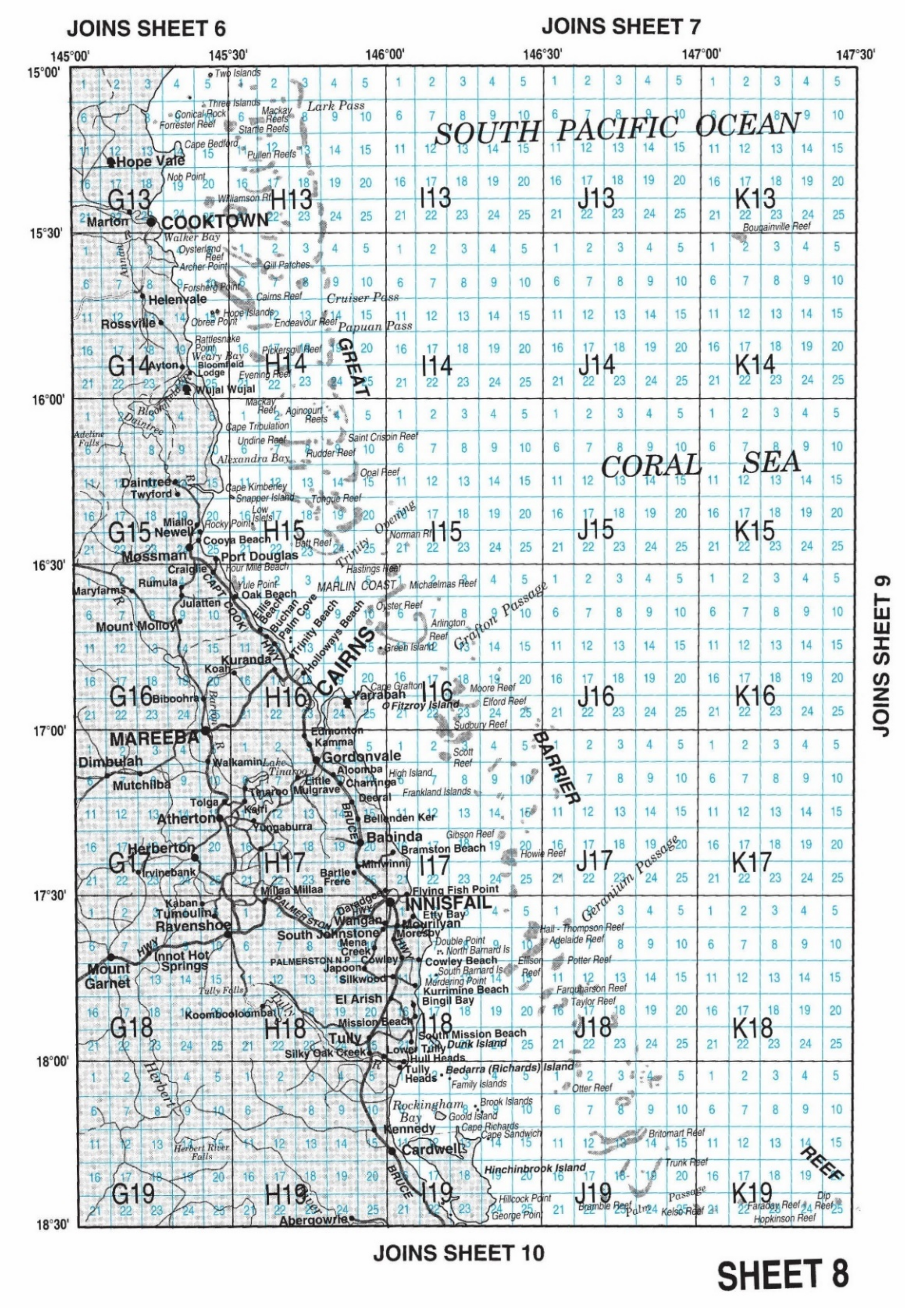

Supplementary Figure 1. Example of commercial fishing logbook map of Queensland.

Commercial fishing logbooks are used to record the retained catch and the location where the catch was taken, using the grids (30 nautical miles) and sites (6 nautical miles). The example provided is ‘Sheet 8’ for the area of the Great Barrier Reef Marine Park near Cairns, Australia.

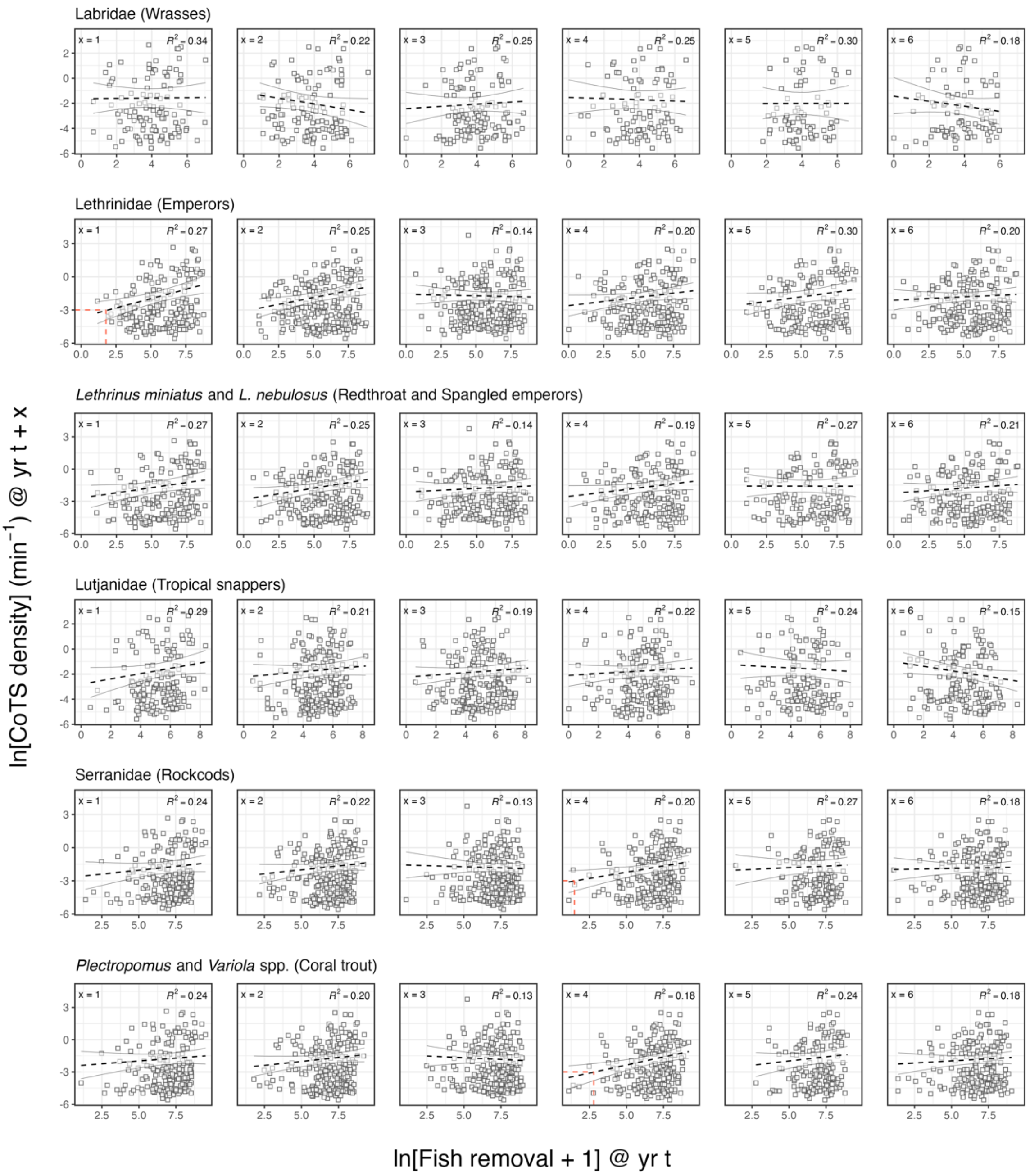

Supplementary Figure 2. Response of Crown-of-Thorns Starfish density to removal of coral reef fish biomass.

Pacific Crown-of-Thorns Starfish (CoTS, *Acanthaster* cf. *solaris*) density at time $t + x$ as a function of coral reef fish biomass removal (kg) at time t evaluated at different values of x. Models were fitted to biomass removal data for six fish groups, namely (1) Labridae

(wrasses), (2) Lethrinidae (emperors), (3) *Lethrinus miniatus* and *L. nebulosus* (redthroat and spangled emperors), (4) Lutjanidae (tropical snappers), (5) Serranidae (rockcods) and (6) *Plectropomus* spp. and *Variola* spp. (coral trout), at six time lags (in years) each. This represents the gamma component of the hurdle-gamma approach to examine whether removal of coral reef fish biomass influences CoTS density in the Great Barrier Reef Marine Park, Australia. Lines and shaded polygons represent respectively the median prediction and associated 95% credible intervals. The slopes correspond to parameter ν_2 (see model description and parameter notation in Supplementary Method 2).

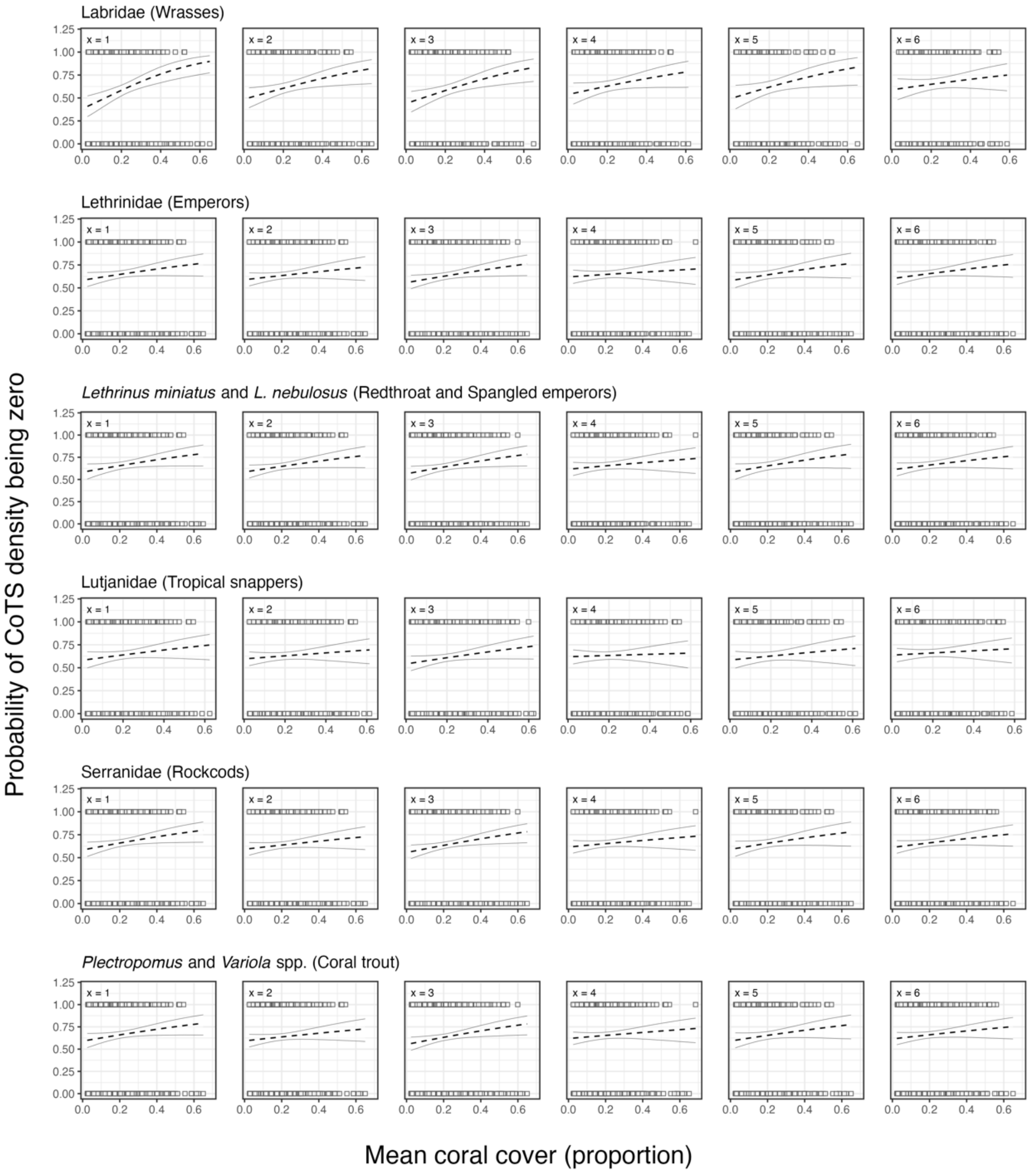

Supplementary Figure 3. Response of Crown-of-Thorns Starfish density to coral cover.

Probability of Pacific Crown-of-Thorns Starfish (CoTS, *Acanthaster* cf. *solaris*) density being zero at time $t + x$ as a function of coral cover at time $t + x$ evaluated at different values of x. Models were fitted to biomass removal data for six fish groups, namely (1) Labridae (wrasses), (2) Lethrinidae (emperors), (3) *Lethrinus miniatus* and *L. nebulosus* (redthroat and spangled emperors), (4) Lutjanidae (tropical snappers), (5) Serranidae

(rockcods) and (6) *Plectropomus* spp. and *Variola* spp. (coral trout), at six time lags (in years) each. This represents the hurdle (logistic) component of the hurdle-gamma approach to examine whether removal of coral reef fish biomass influences CoTS density in the Great Barrier Reef Marine Park, Australia. Lines and shaded polygons represent respectively the mean prediction and associated 95% credible intervals. The slopes correspond to the probability evaluated as $\ln\left(\mathrm{Bern}\left(1|\mathrm{logit}^{-1}(\lambda_{\mathrm{i,t,x}})\right)\right)$ (see model description and parameter notation in the Supplementary Method 2).

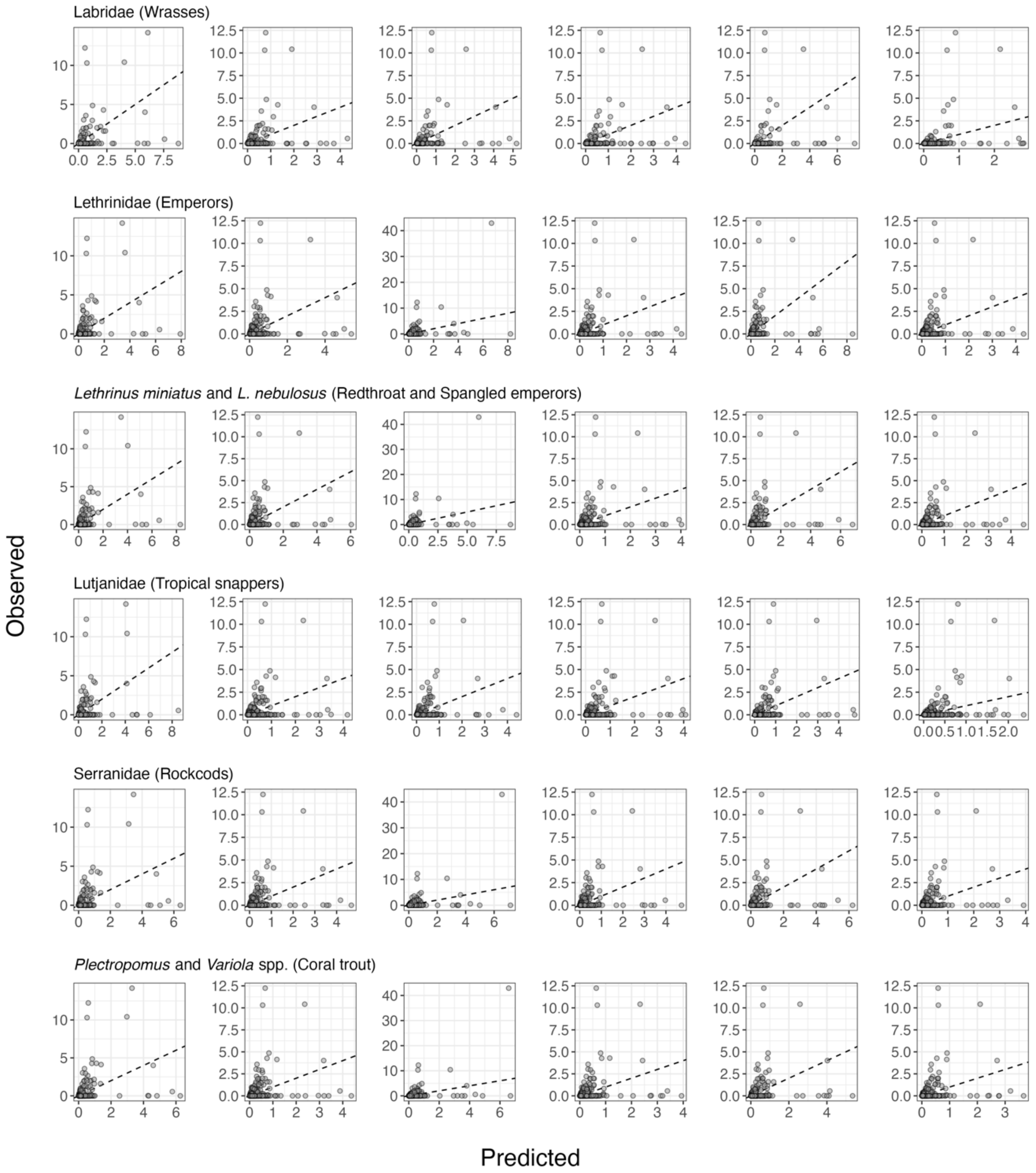

Supplementary Figure 4. Posterior predictive check of the hurdle-gamma model.

The model was used to examine whether removal of coral reef fish biomass influences density of Pacific Crown-of-Thorns Starfish (CoTS, *Acanthaster* cf. *solaris*) in the Great Barrier Reef Marine Park, Australia. Observed values on the y axis, and observation-level mean posterior prediction on the x axis. In each row, time lags increase (from one through to

six) from left to right. Dashed line represents a 1-to-1 fit. See model description in the Supplementary Method 2.

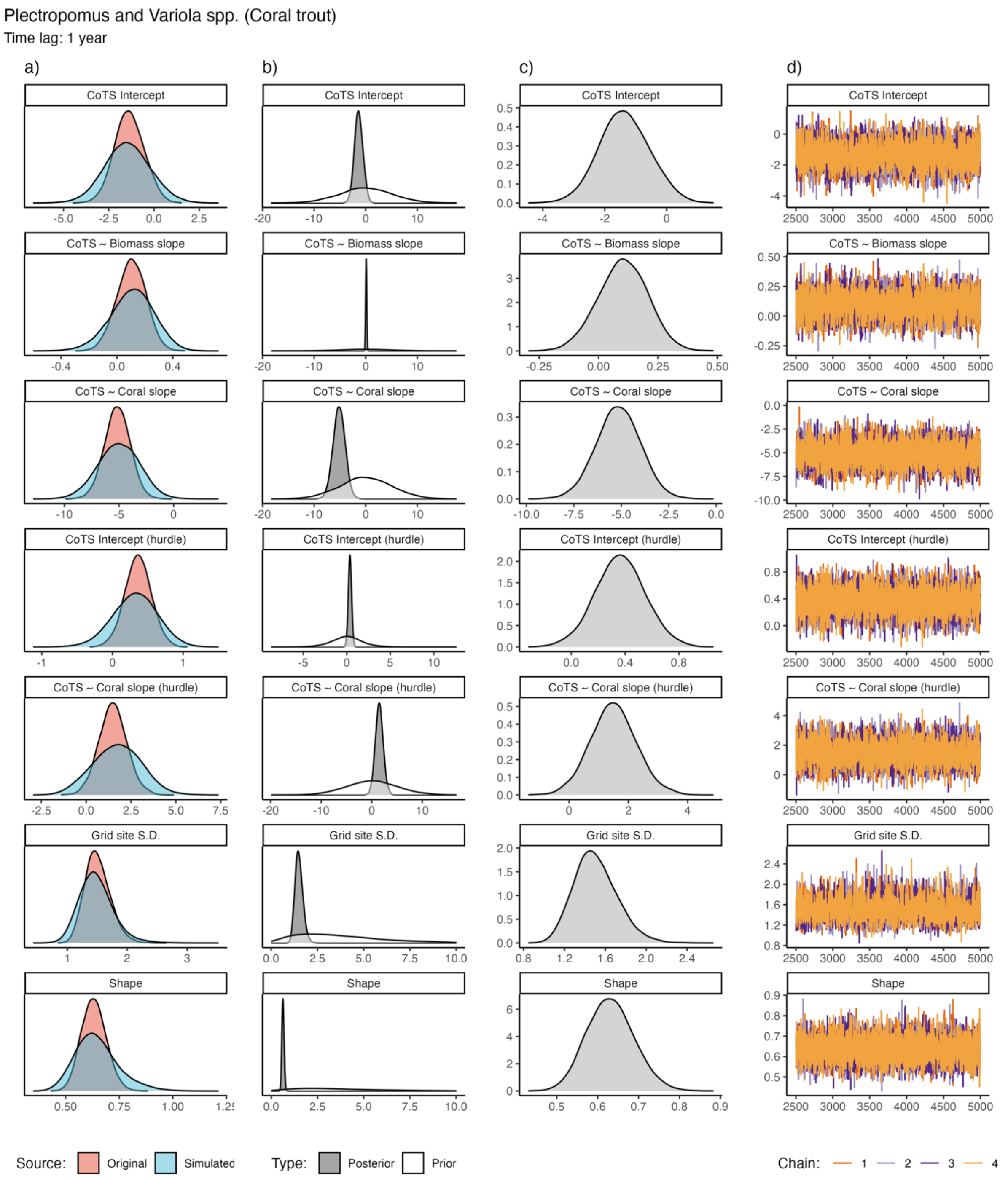

Supplementary Figure 5. Model validation checks.

a) Comparison between original posterior distributions of model parameters (red) on top of the combination of all posterior distributions across all 500 runs (blue, see Supplementary Method 2). b) Comparison between prior and posterior distribution of model parameters. c) Posterior distribution of model parameters. d) Chain mixing trace-plots.

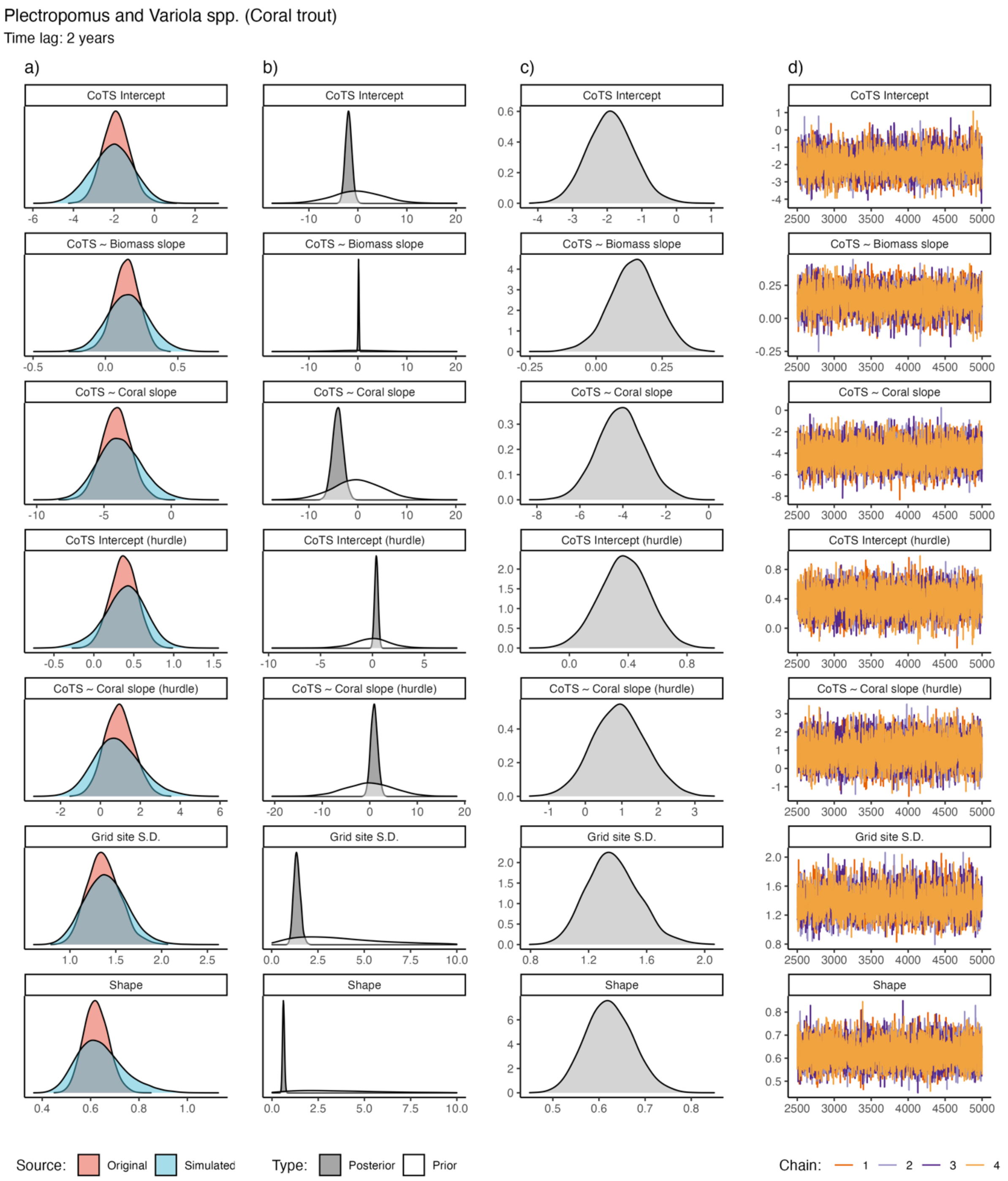

Supplementary Figure 6. Model validation checks.

a) Comparison between original posterior distributions of model parameters (red) on top of the combination of all posterior distributions across all 500 runs (blue, see Supplementary Method 2). b) Comparison between prior and posterior distribution of model parameters. c) Posterior distribution of model parameters. d) Chain mixing trace-plots.

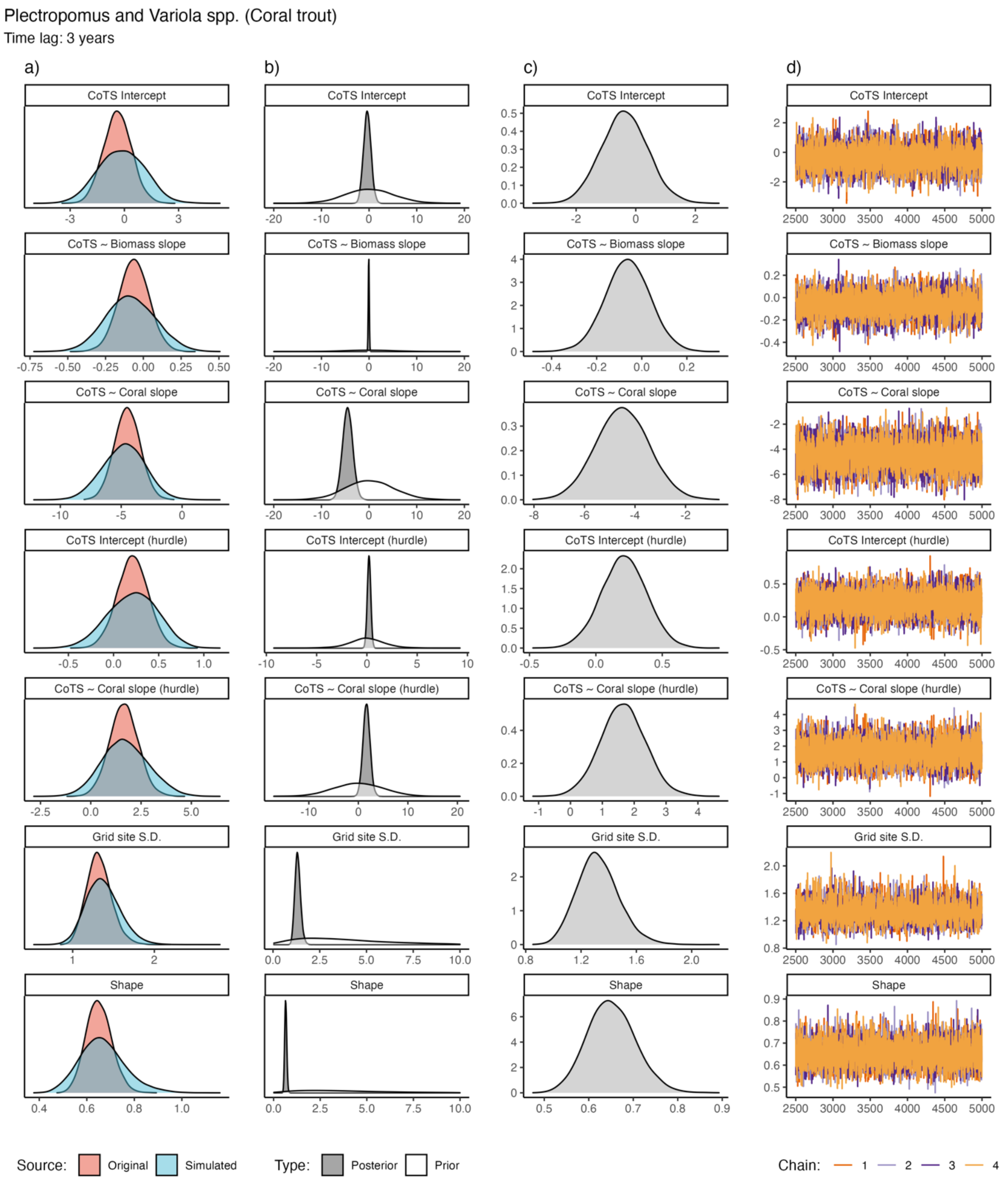

Supplementary Figure 7. Model validation checks.

a) Comparison between original posterior distributions of model parameters (red) on top of the combination of all posterior distributions across all 500 runs (blue, see Supplementary Method 2). b) Comparison between prior and posterior distribution of model parameters. c) Posterior distribution of model parameters. d) Chain mixing trace-plots.

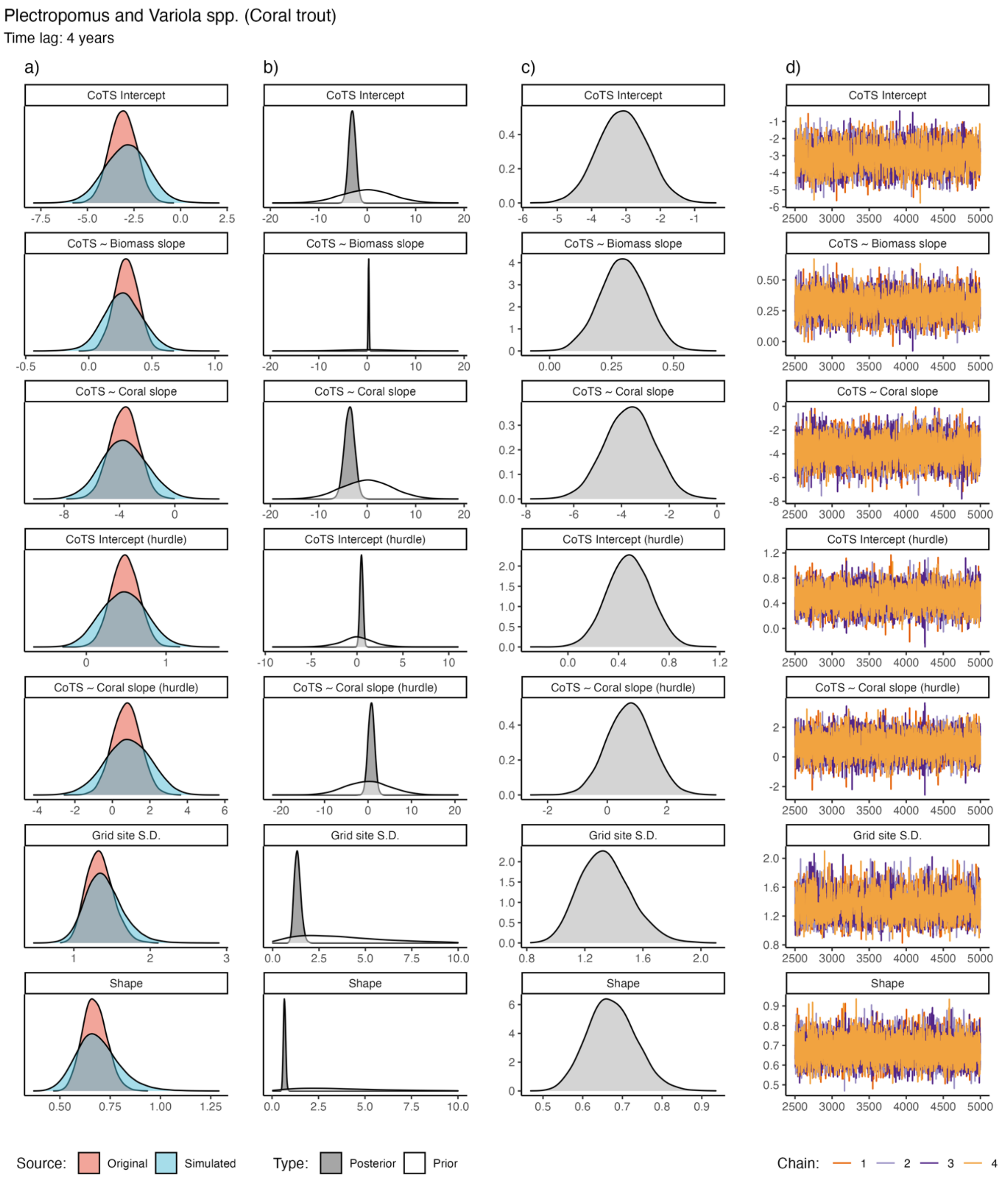

Supplementary Figure 8. Model validation checks.

a) Comparison between original posterior distributions of model parameters (red) on top of the combination of all posterior distributions across all 500 runs (blue, see Supplementary Method 2). b) Comparison between prior and posterior distribution of model parameters. c) Posterior distribution of model parameters. d) Chain mixing trace-plots.

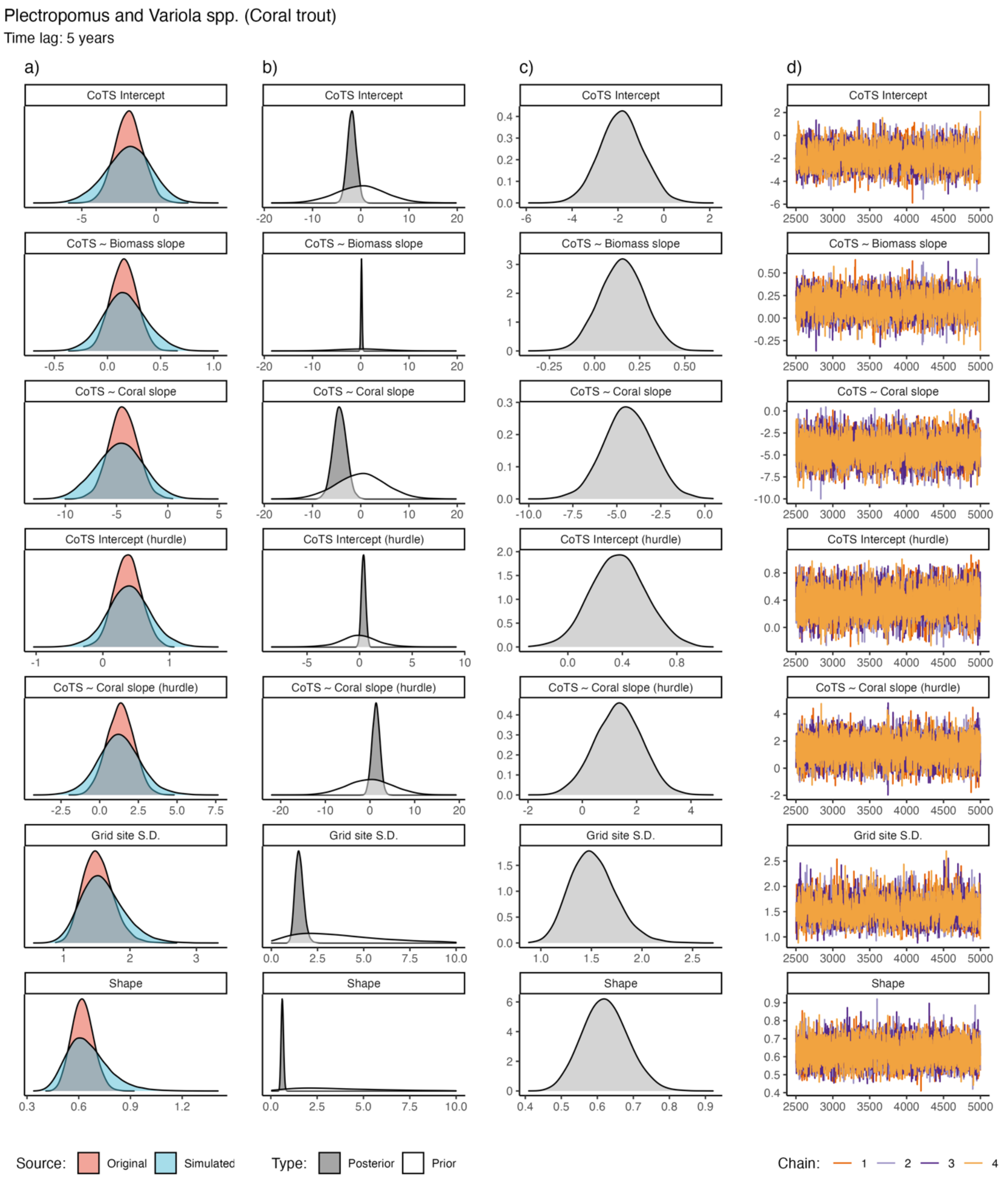

Supplementary Figure 9. Model validation checks.

a) Comparison between original posterior distributions of model parameters (red) on top of the combination of all posterior distributions across all 500 runs (blue, see Supplementary Method 2). b) Comparison between prior and posterior distribution of model parameters. c) Posterior distribution of model parameters. d) Chain mixing trace-plots.

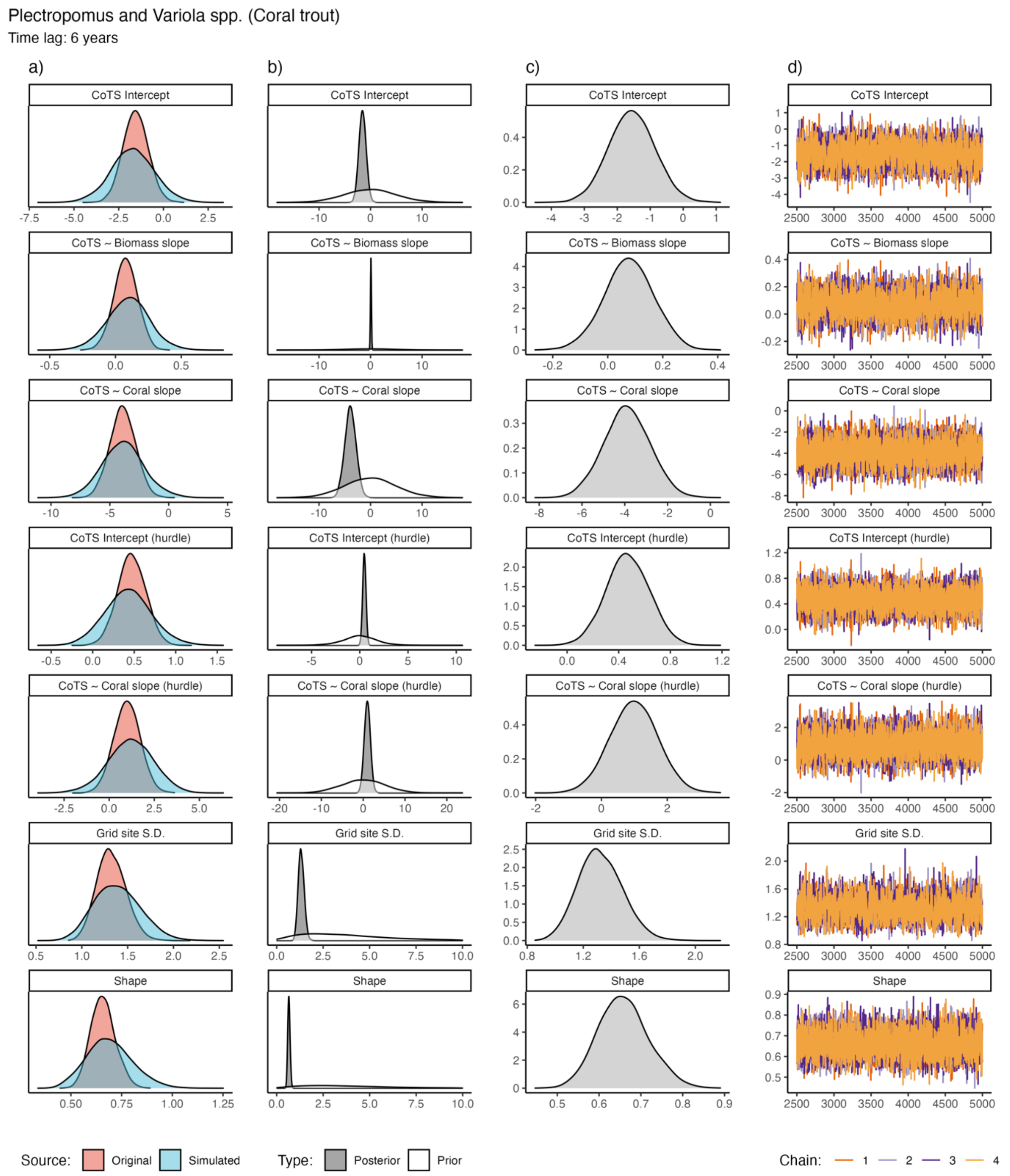

Supplementary Figure 10. Model validation checks.

a) Comparison between original posterior distributions of model parameters (red) on top of the combination of all posterior distributions across all 500 runs (blue, see Supplementary Method 2). b) Comparison between prior and posterior distribution of model parameters. c) Posterior distribution of model parameters. d) Chain mixing trace-plots.

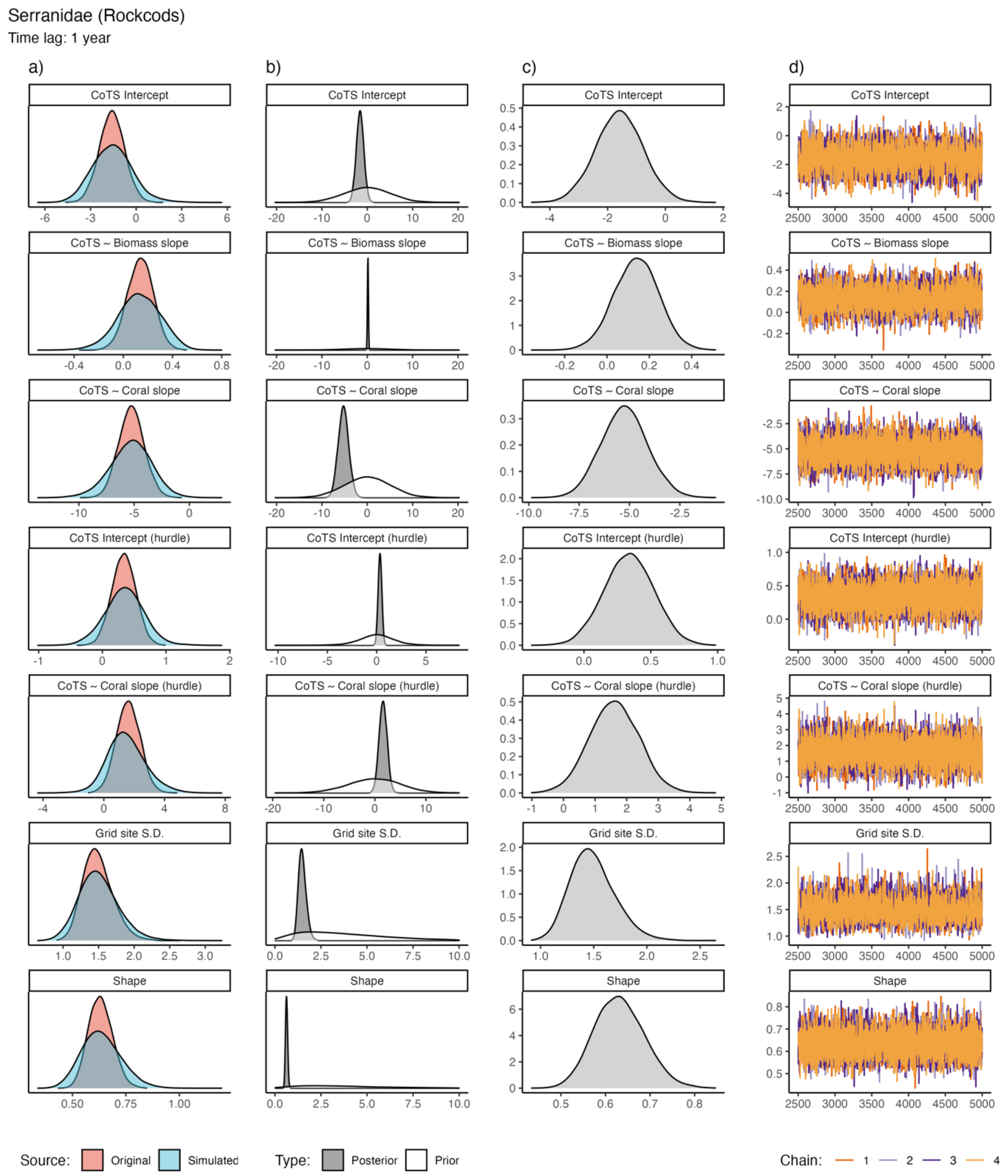

Supplementary Figure 11. Model validation checks.

a) Comparison between original posterior distributions of model parameters (red) on top of the combination of all posterior distributions across all 500 runs (blue, see Supplementary Method 2). b) Comparison between prior and posterior distribution of model parameters. c) Posterior distribution of model parameters. d) Chain mixing trace-plots.

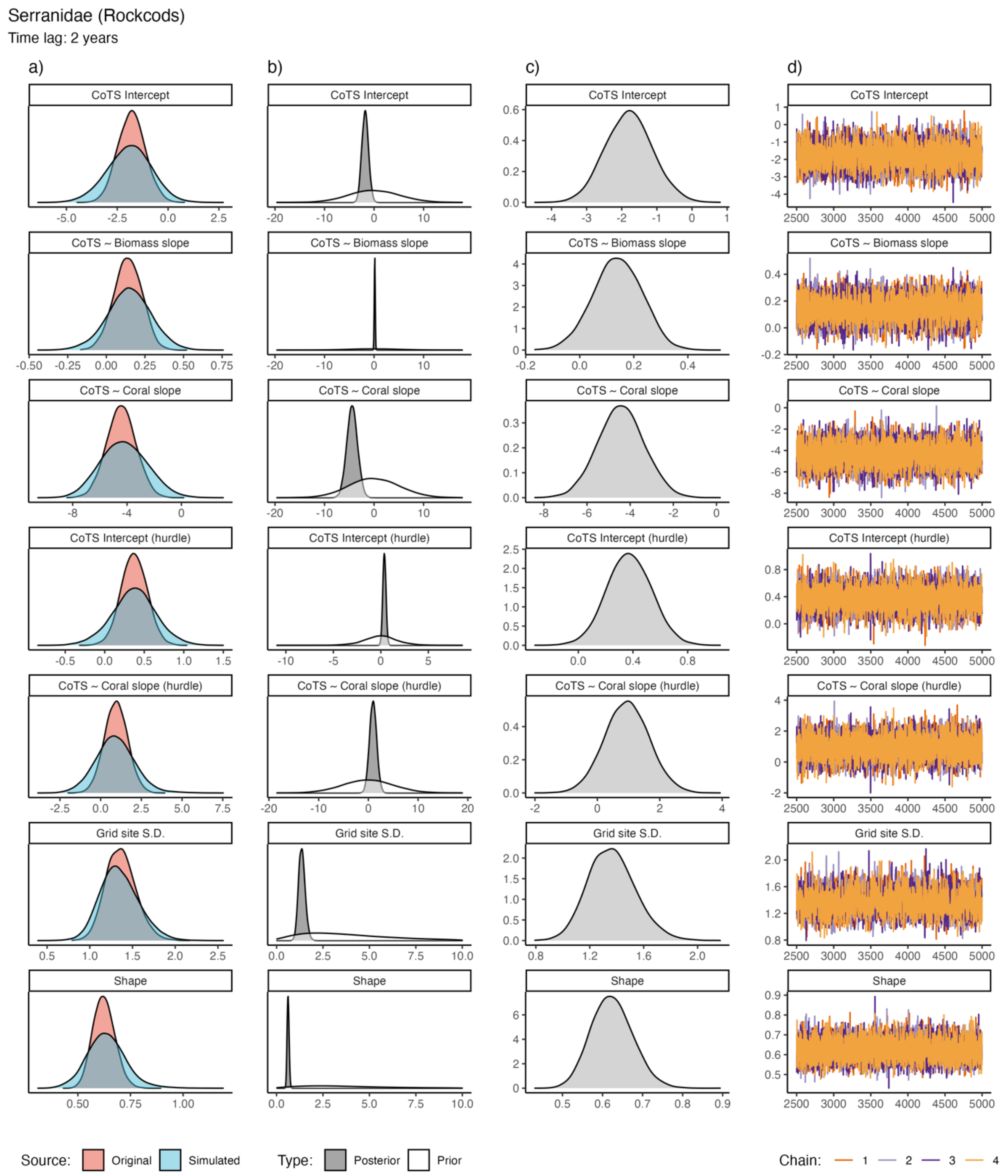

Supplementary Figure 12. Model validation checks.

a) Comparison between original posterior distributions of model parameters (red) on top of the combination of all posterior distributions across all 500 runs (blue, see Supplementary Method 2). b) Comparison between prior and posterior distribution of model parameters. c) Posterior distribution of model parameters. d) Chain mixing trace-plots.

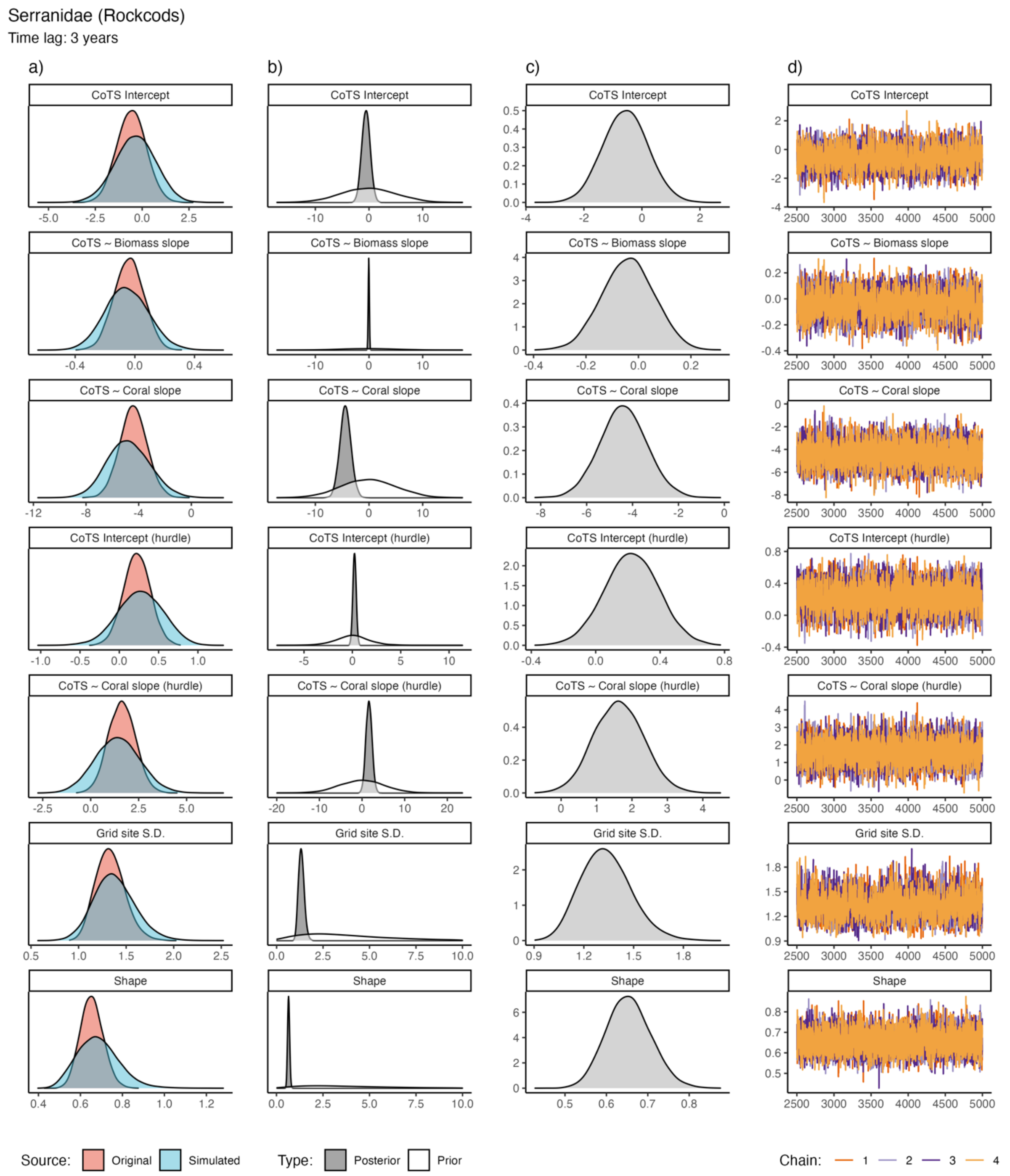

Supplementary Figure 13. Model validation checks.

a) Comparison between original posterior distributions of model parameters (red) on top of the combination of all posterior distributions across all 500 runs (blue, see Supplementary Method 2). b) Comparison between prior and posterior distribution of model parameters. c) Posterior distribution of model parameters. d) Chain mixing trace-plots.

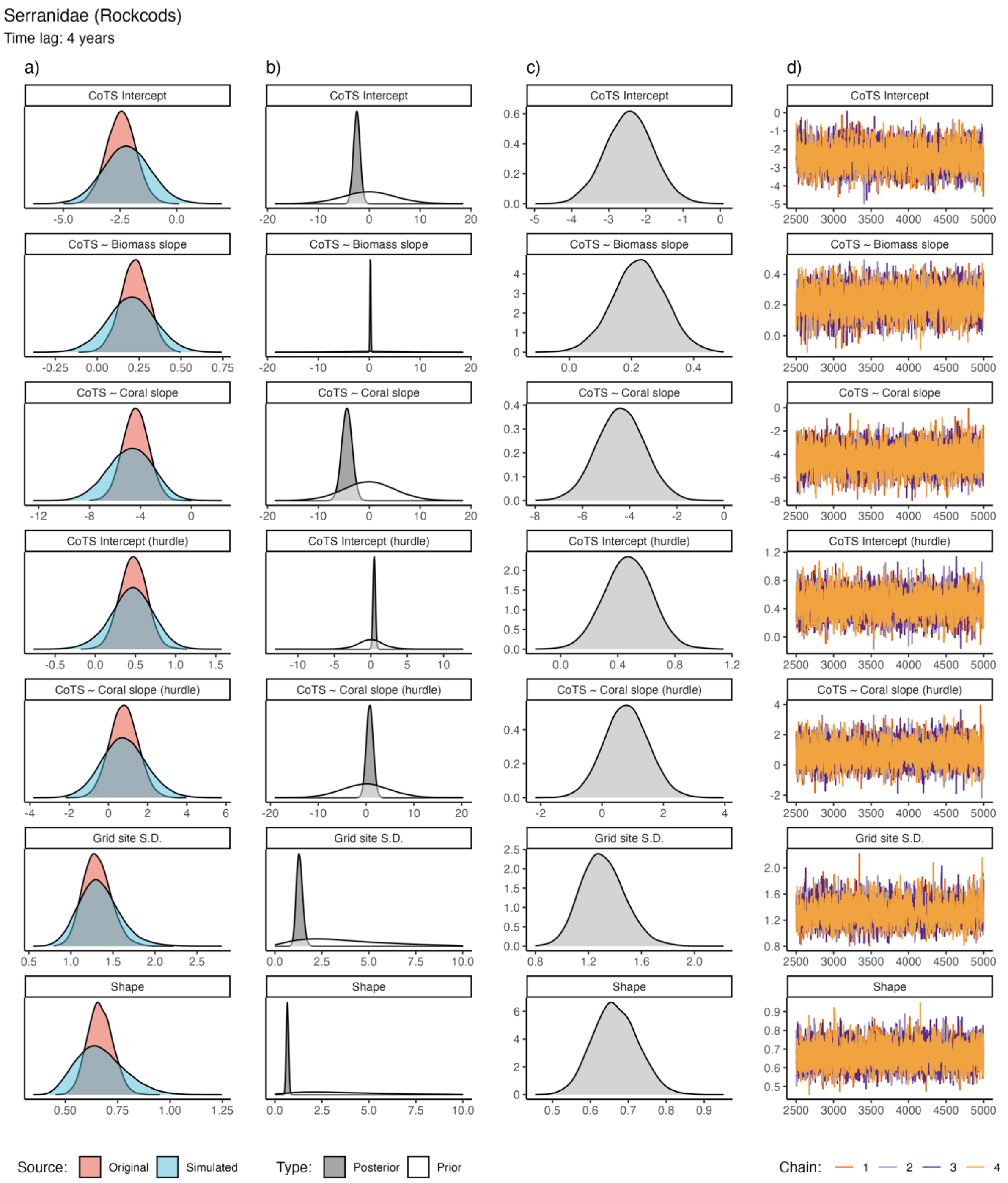

Supplementary Figure 14. Model validation checks.

a) Comparison between original posterior distributions of model parameters (red) on top of the combination of all posterior distributions across all 500 runs (blue, see Supplementary Method 2). b) Comparison between prior and posterior distribution of model parameters. c) Posterior distribution of model parameters. d) Chain mixing trace-plots.

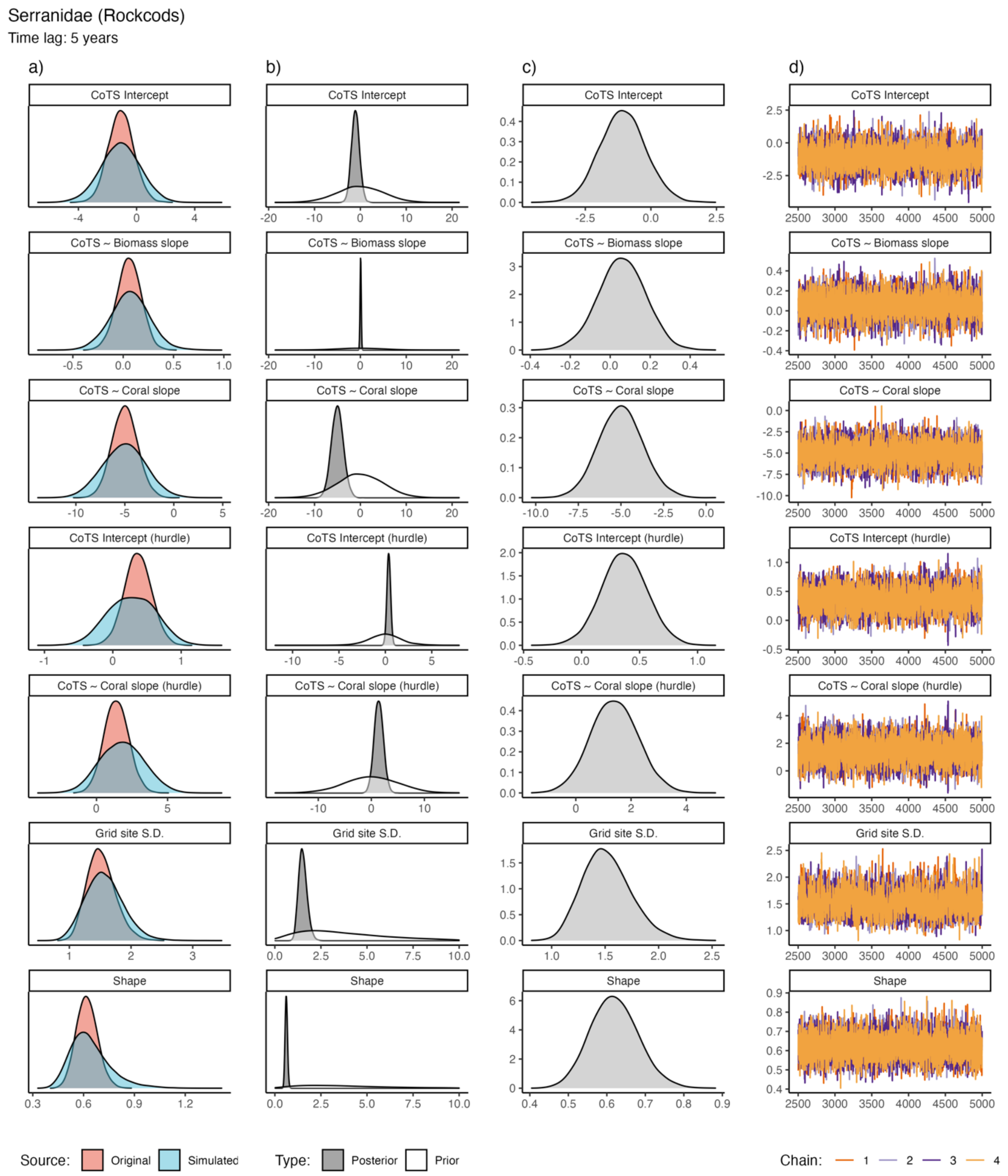

Supplementary Figure 15. Model validation checks.

a) Comparison between original posterior distributions of model parameters (red) on top of the combination of all posterior distributions across all 500 runs (blue, see Supplementary Method 2). b) Comparison between prior and posterior distribution of model parameters. c) Posterior distribution of model parameters. d) Chain mixing trace-plots.

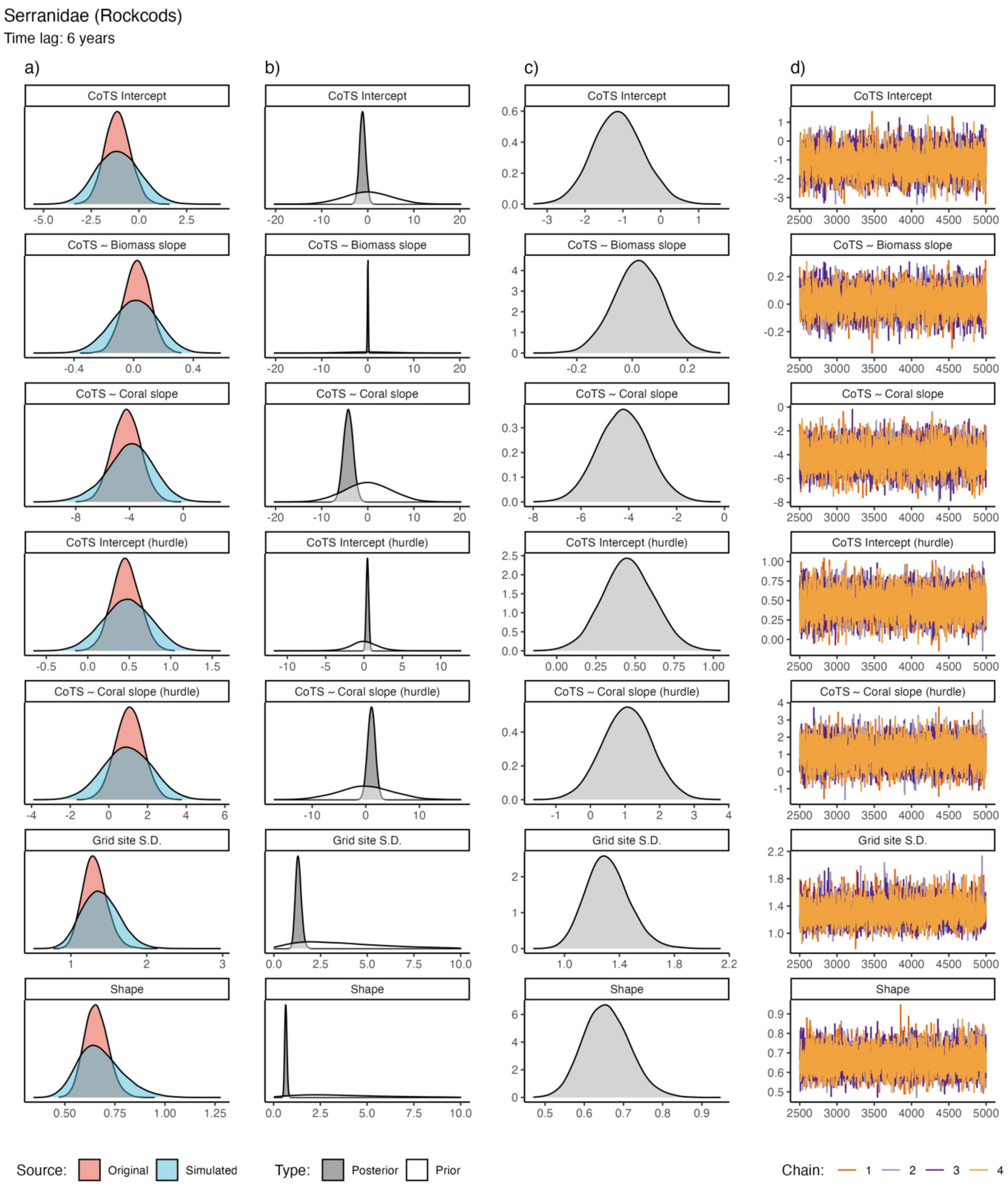

Supplementary Figure 16. Model validation checks.

a) Comparison between original posterior distributions of model parameters (red) on top of the combination of all posterior distributions across all 500 runs (blue, see Supplementary Method 2). b) Comparison between prior and posterior distribution of model parameters. c) Posterior distribution of model parameters. d) Chain mixing trace-plots.

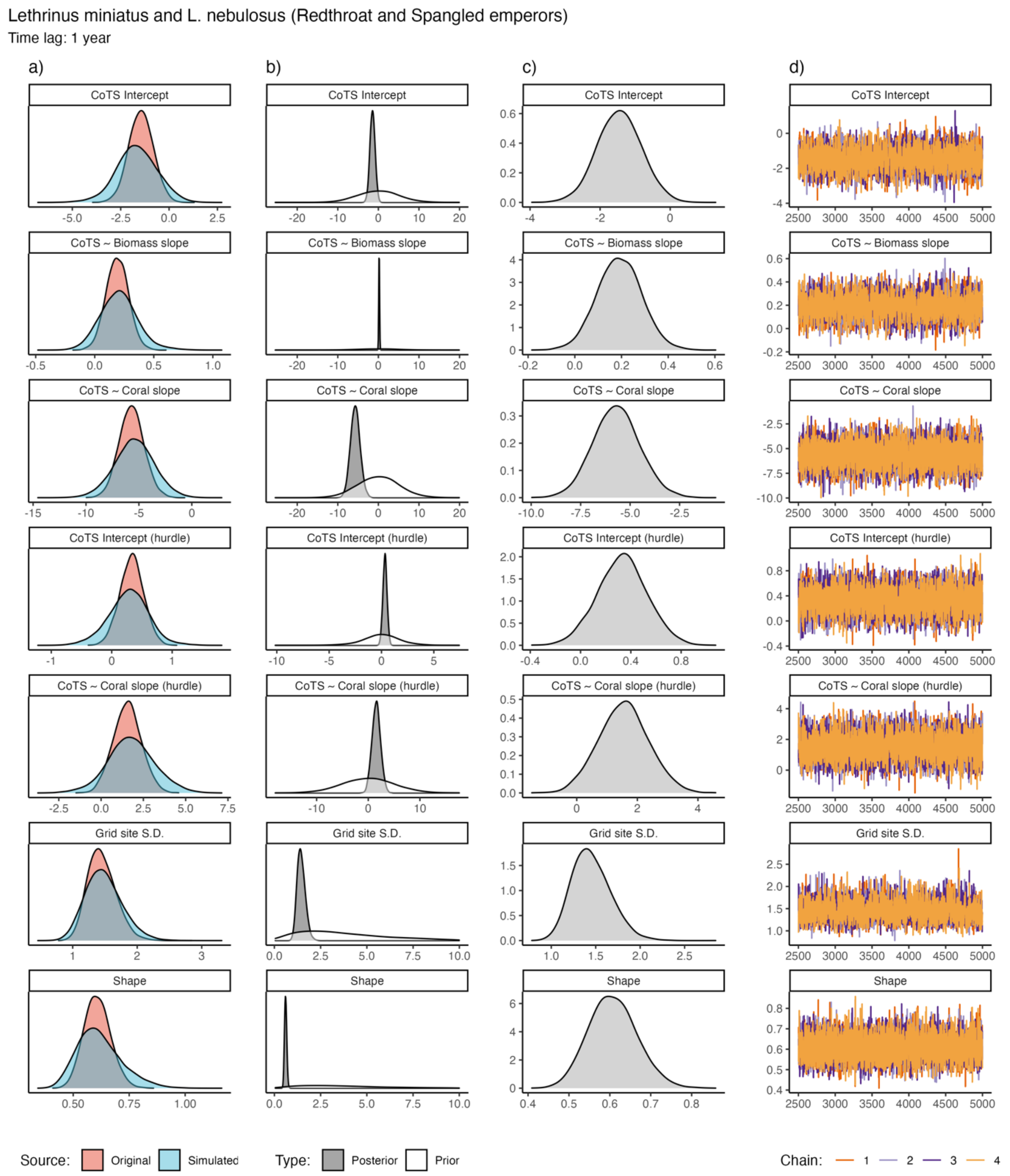

Supplementary Figure 17. Model validation checks.

a) Comparison between original posterior distributions of model parameters (red) on top of the combination of all posterior distributions across all 500 runs (blue, see Supplementary Method 2). b) Comparison between prior and posterior distribution of model parameters. c) Posterior distribution of model parameters. d) Chain mixing trace-plots.

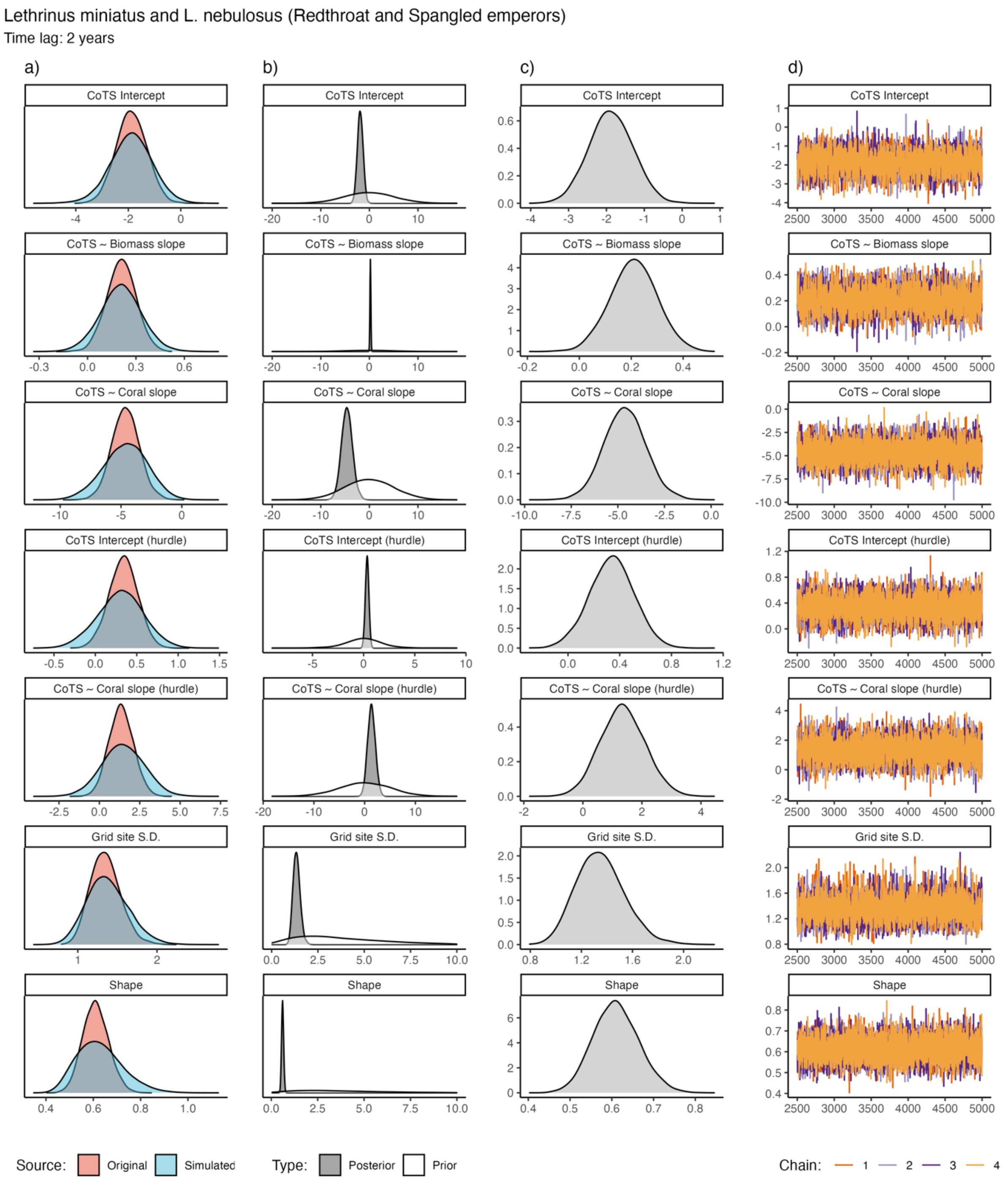

Supplementary Figure 18. Model validation checks.

a) Comparison between original posterior distributions of model parameters (red) on top of the combination of all posterior distributions across all 500 runs (blue, see Supplementary Method 2). b) Comparison between prior and posterior distribution of model parameters. c) Posterior distribution of model parameters. d) Chain mixing trace-plots.

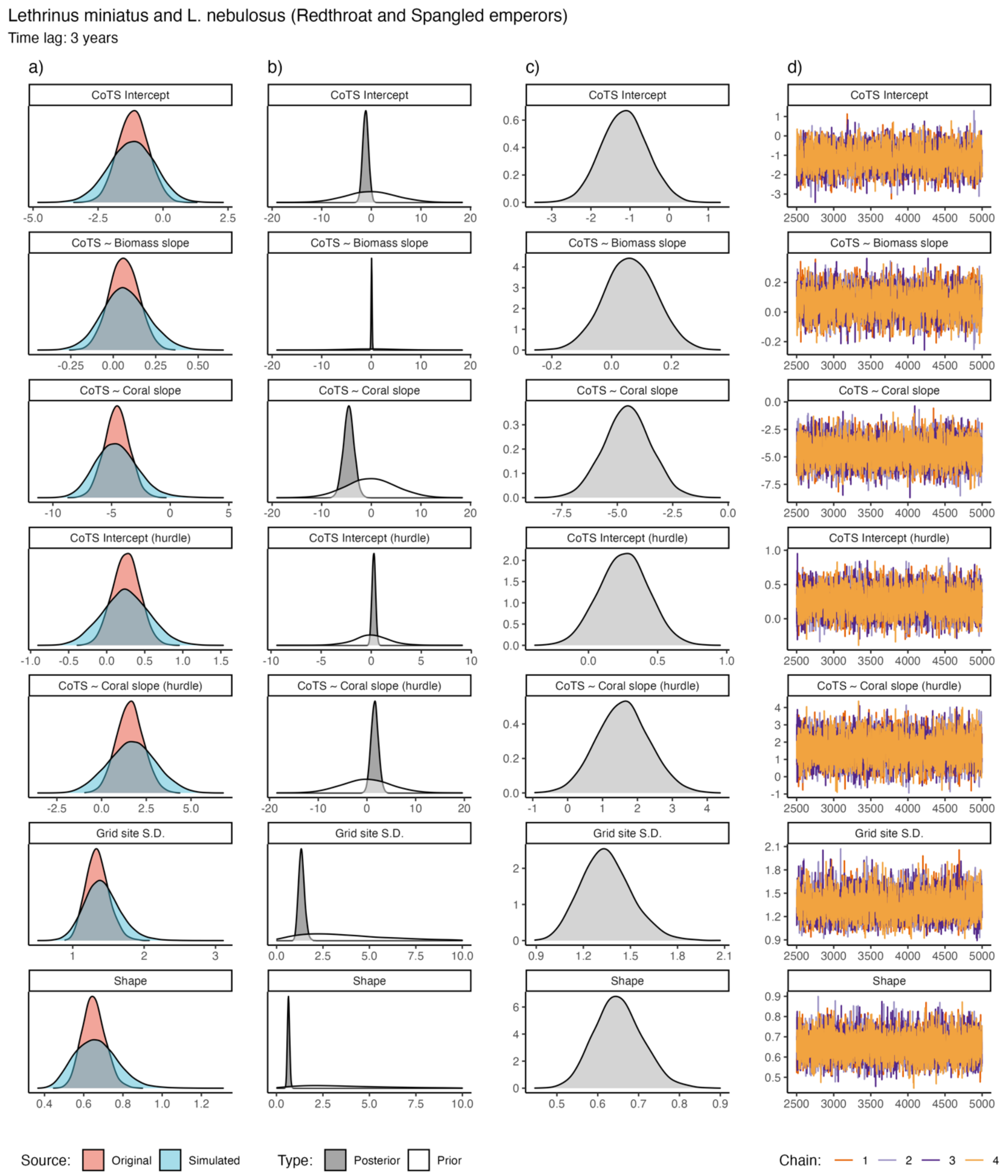

Supplementary Figure 19. Model validation checks.

a) Comparison between original posterior distributions of model parameters (red) on top of the combination of all posterior distributions across all 500 runs (blue, see Supplementary Method 2). b) Comparison between prior and posterior distribution of model parameters. c) Posterior distribution of model parameters. d) Chain mixing trace-plots.

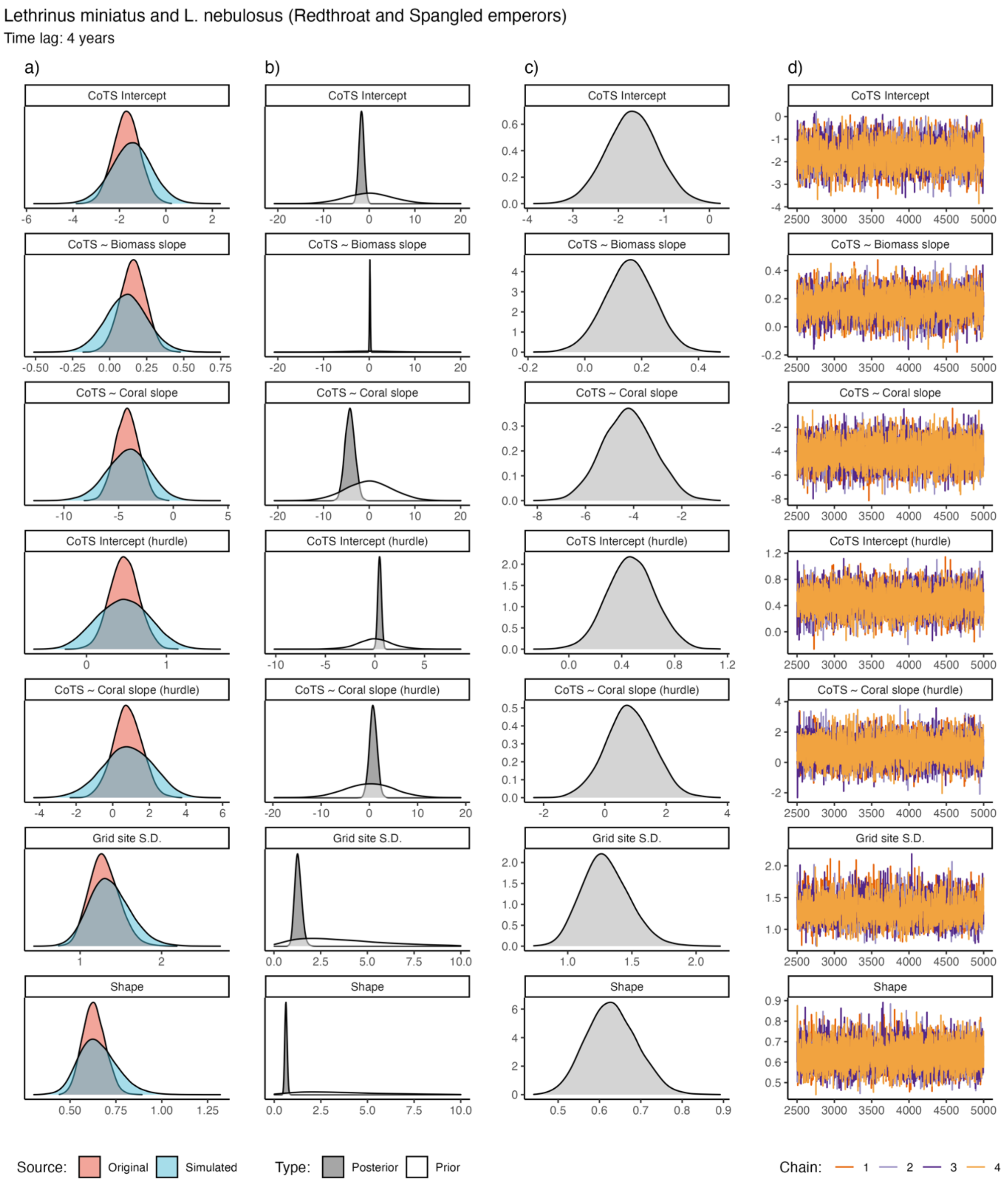

Supplementary Figure 20. Model validation checks.

a) Comparison between original posterior distributions of model parameters (red) on top of the combination of all posterior distributions across all 500 runs (blue, see Supplementary Method 2). b) Comparison between prior and posterior distribution of model parameters. c) Posterior distribution of model parameters. d) Chain mixing trace-plots.

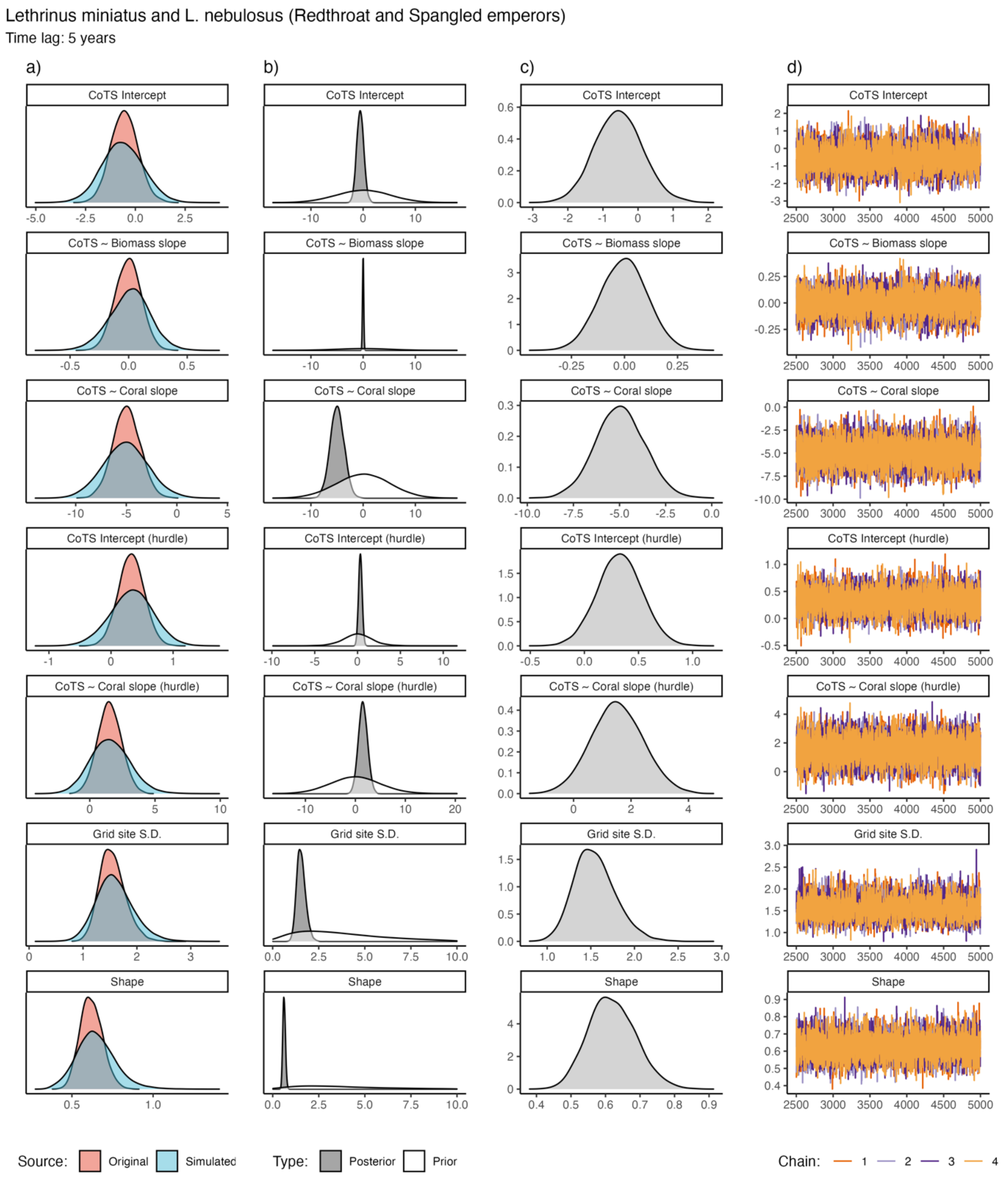

Supplementary Figure 21. Model validation checks.

a) Comparison between original posterior distributions of model parameters (red) on top of the combination of all posterior distributions across all 500 runs (blue, see Supplementary Method 2). b) Comparison between prior and posterior distribution of model parameters. c) Posterior distribution of model parameters. d) Chain mixing trace-plots.

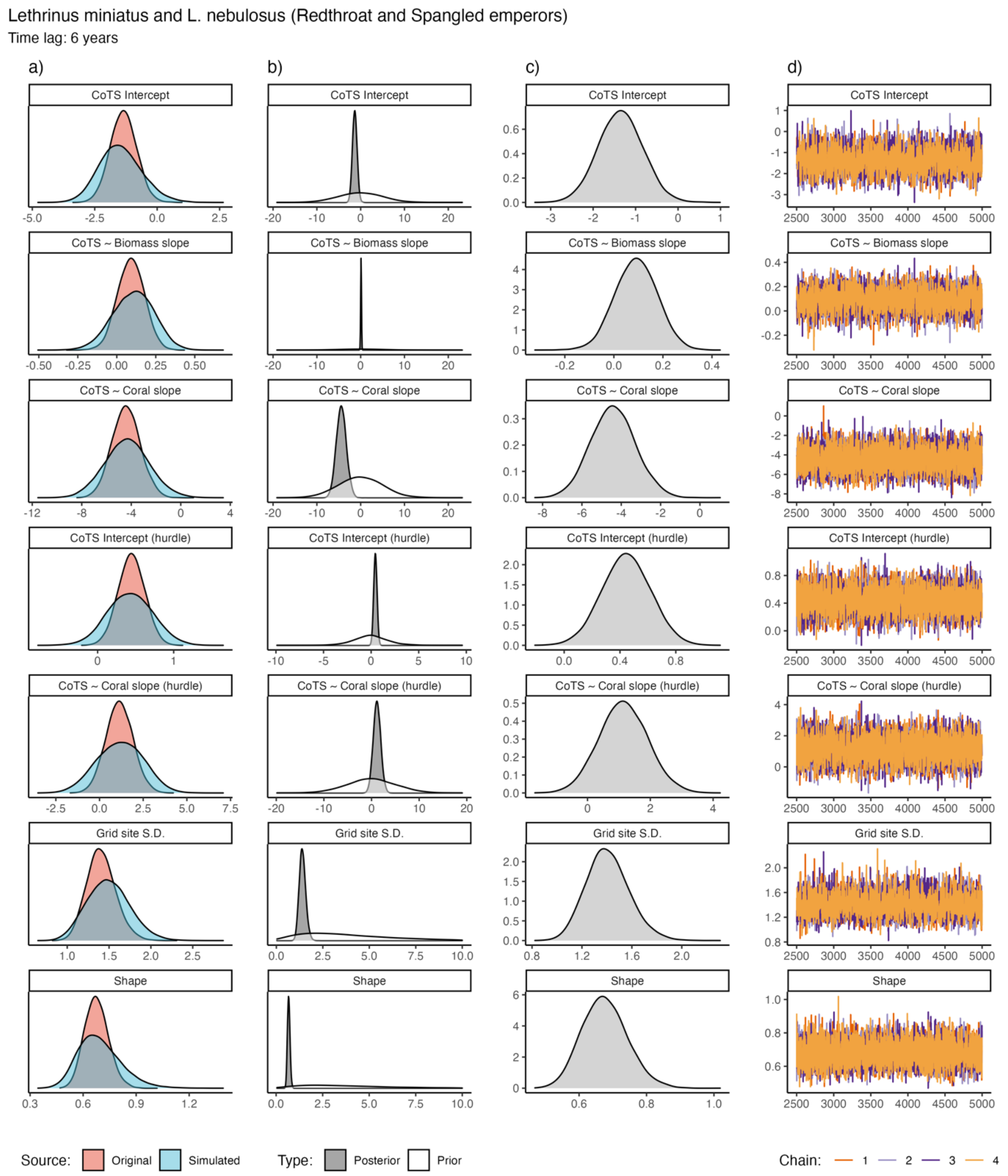

Supplementary Figure 22. Model validation checks.

a) Comparison between original posterior distributions of model parameters (red) on top of the combination of all posterior distributions across all 500 runs (blue, see Supplementary Method 2). b) Comparison between prior and posterior distribution of model parameters. c) Posterior distribution of model parameters. d) Chain mixing trace-plots.

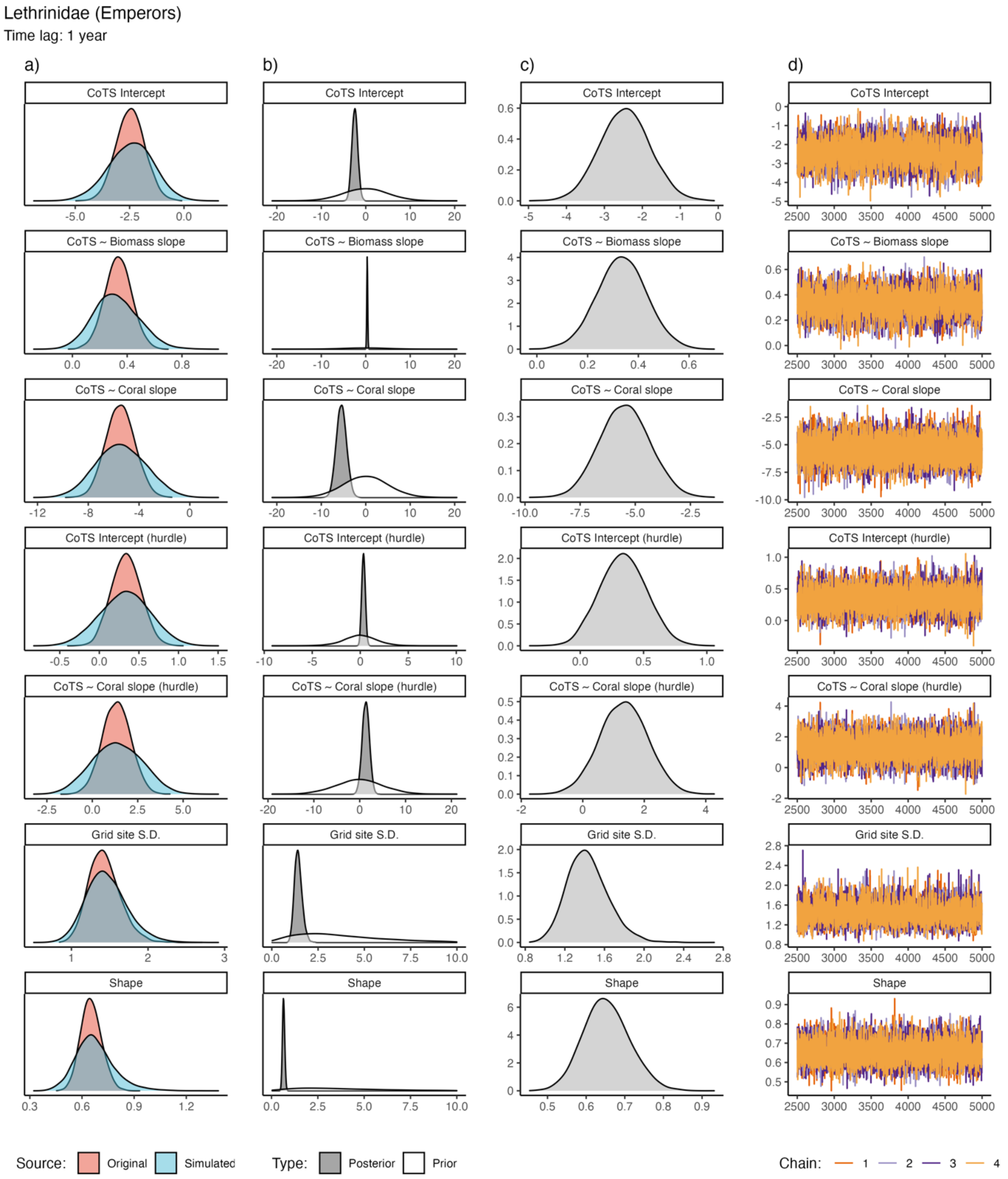

Supplementary Figure 23. Model validation checks.

a) Comparison between original posterior distributions of model parameters (red) on top of the combination of all posterior distributions across all 500 runs (blue, see Supplementary Method 2). b) Comparison between prior and posterior distribution of model parameters. c) Posterior distribution of model parameters. d) Chain mixing trace-plots.

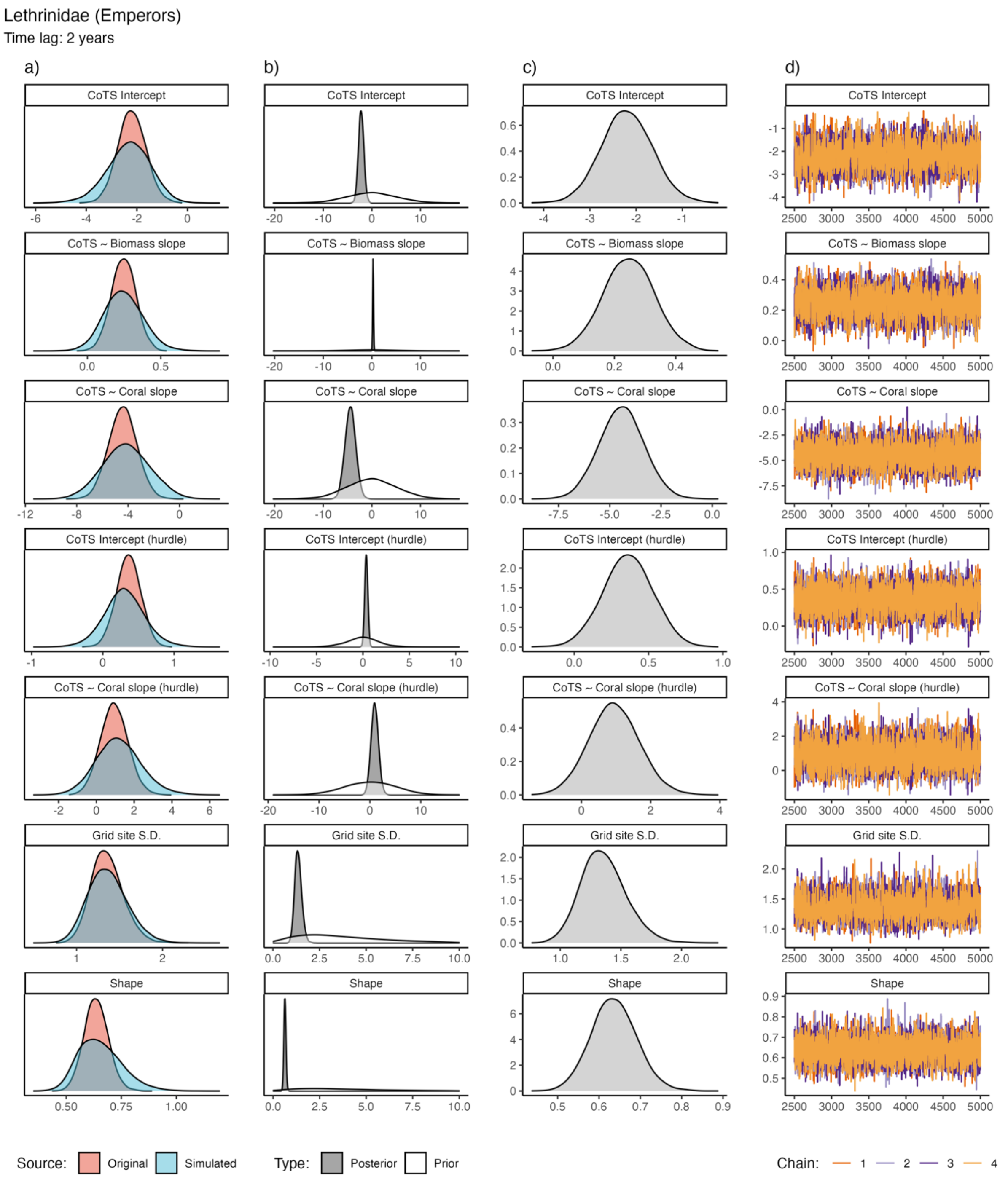

Supplementary Figure 24. Model validation checks.

a) Comparison between original posterior distributions of model parameters (red) on top of the combination of all posterior distributions across all 500 runs (blue, see Supplementary Method 2). b) Comparison between prior and posterior distribution of model parameters. c) Posterior distribution of model parameters. d) Chain mixing trace-plots.

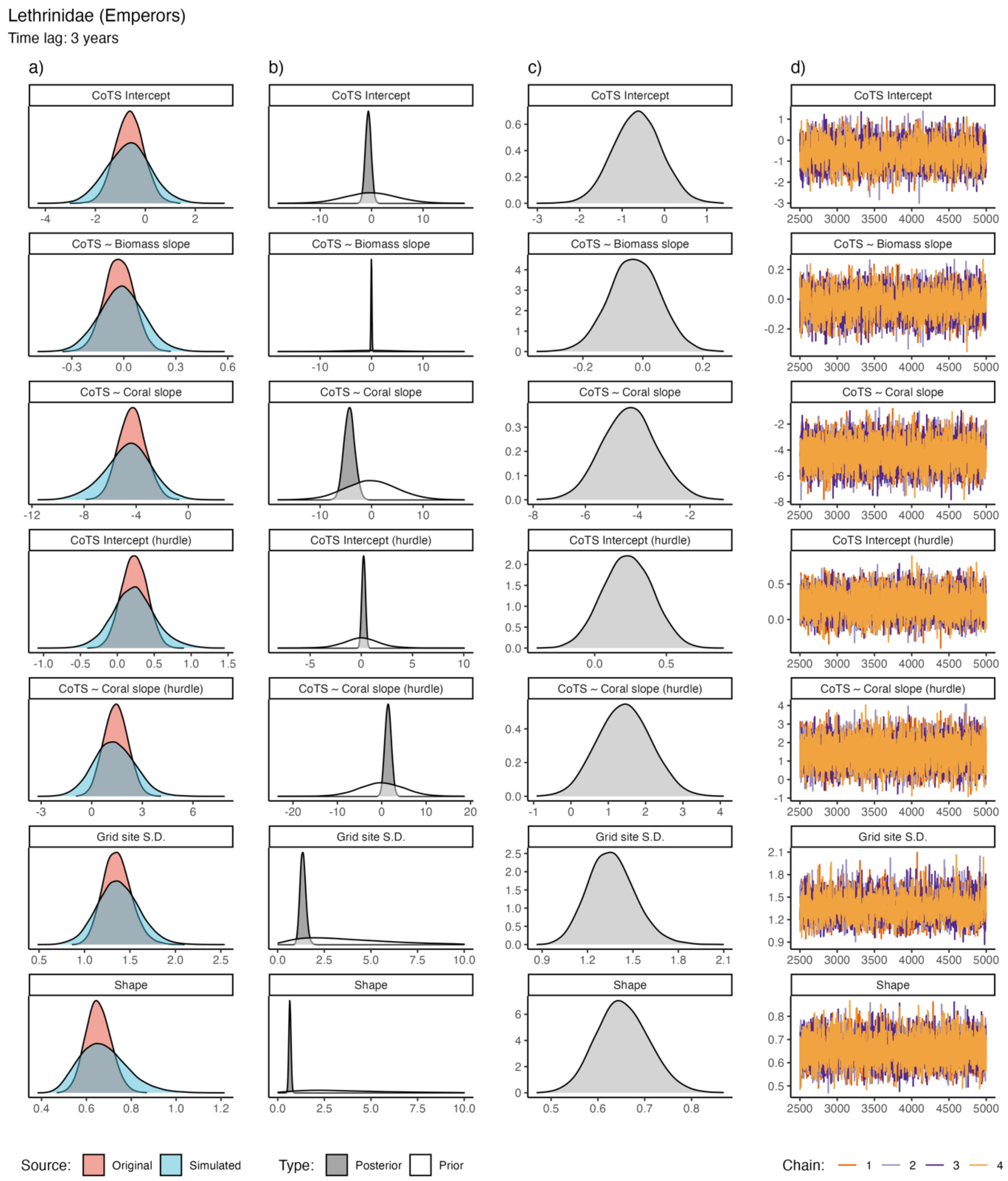

Supplementary Figure 25. Model validation checks.

a) Comparison between original posterior distributions of model parameters (red) on top of the combination of all posterior distributions across all 500 runs (blue, see Supplementary Method 2). b) Comparison between prior and posterior distribution of model parameters. c) Posterior distribution of model parameters. d) Chain mixing trace-plots.

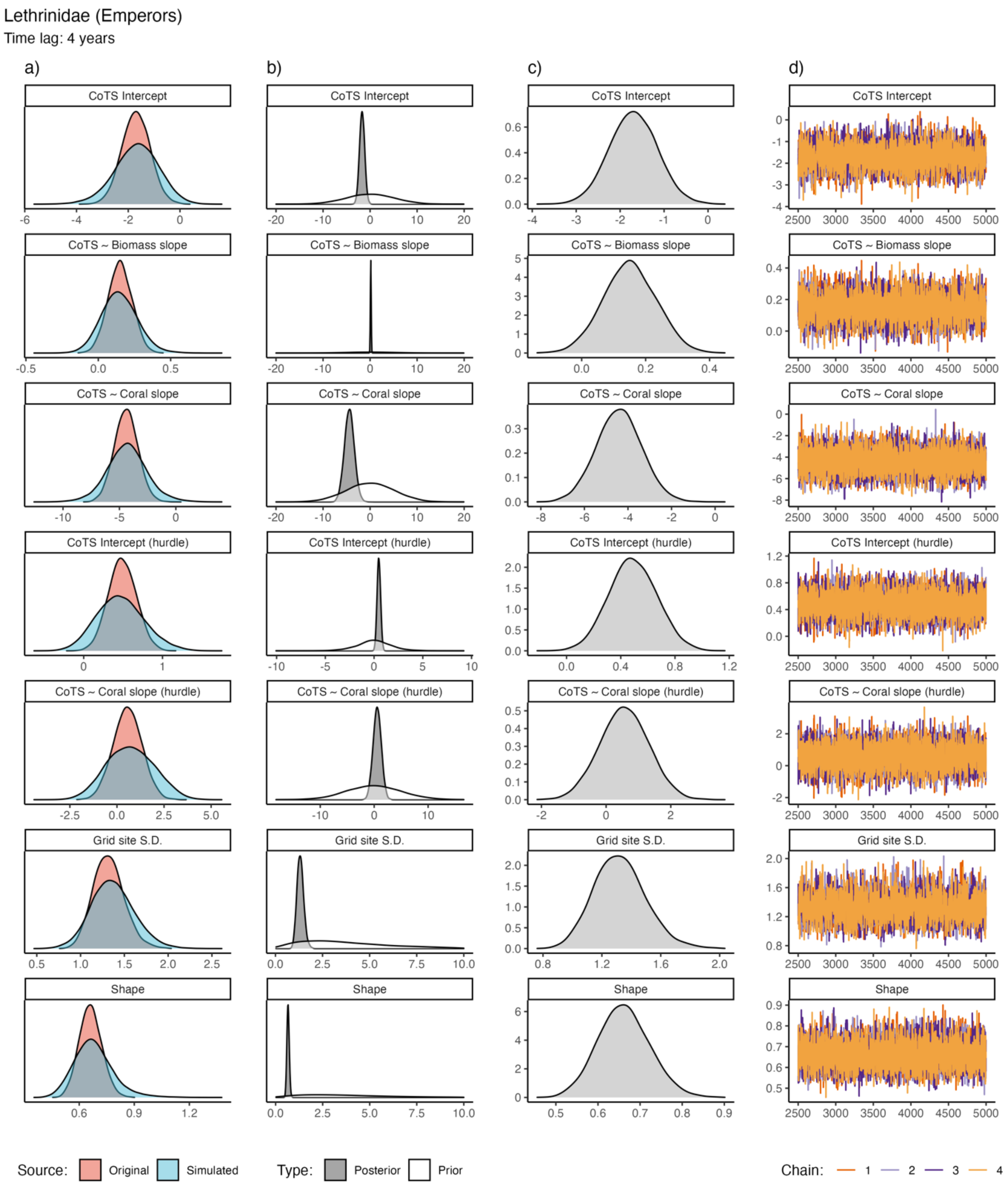

Supplementary Figure 26. Model validation checks.

a) Comparison between original posterior distributions of model parameters (red) on top of the combination of all posterior distributions across all 500 runs (blue, see Supplementary Method 2). b) Comparison between prior and posterior distribution of model parameters. c) Posterior distribution of model parameters. d) Chain mixing trace-plots.

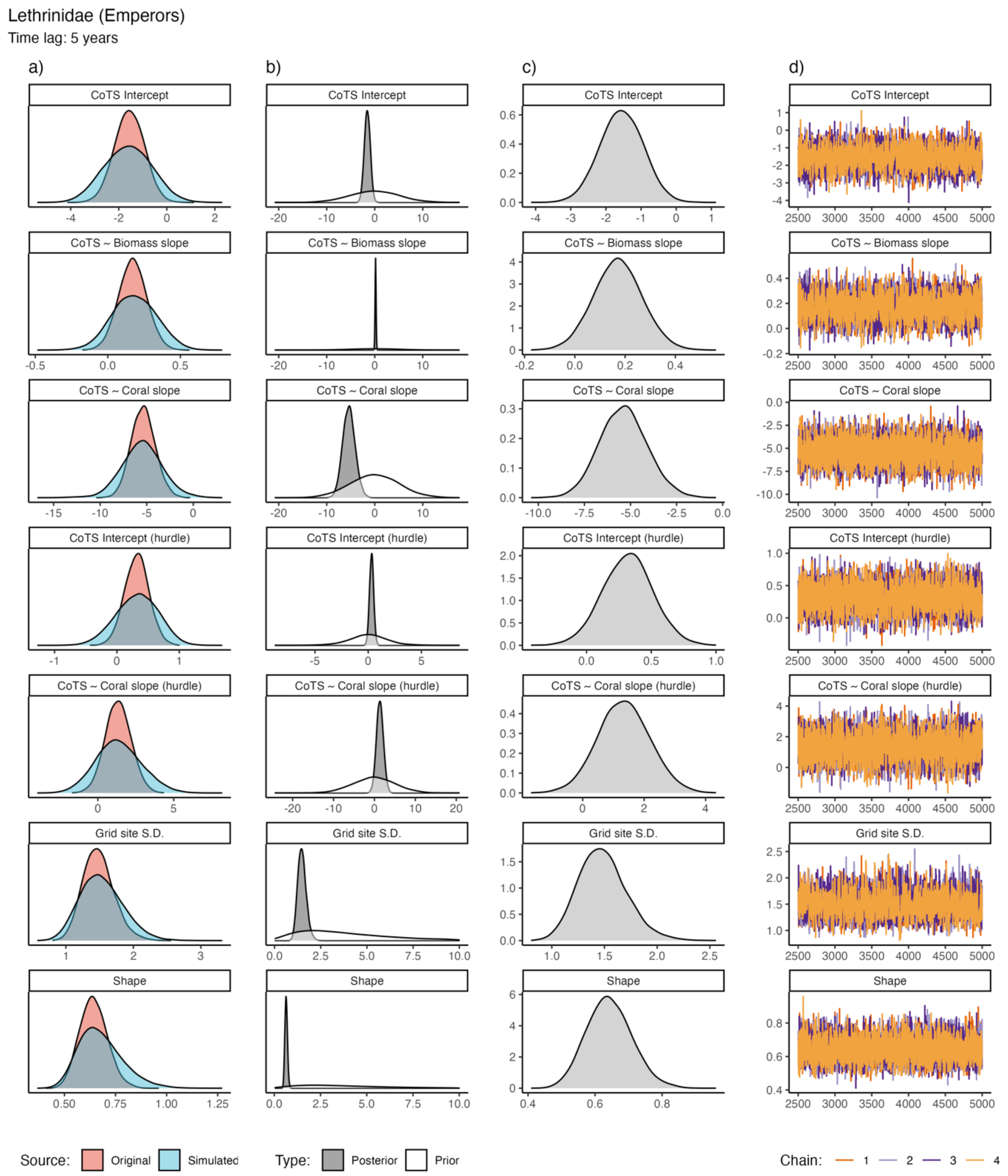

Supplementary Figure 27. Model validation checks.

a) Comparison between original posterior distributions of model parameters (red) on top of the combination of all posterior distributions across all 500 runs (blue, see Supplementary Method 2). b) Comparison between prior and posterior distribution of model parameters. c) Posterior distribution of model parameters. d) Chain mixing trace-plots.

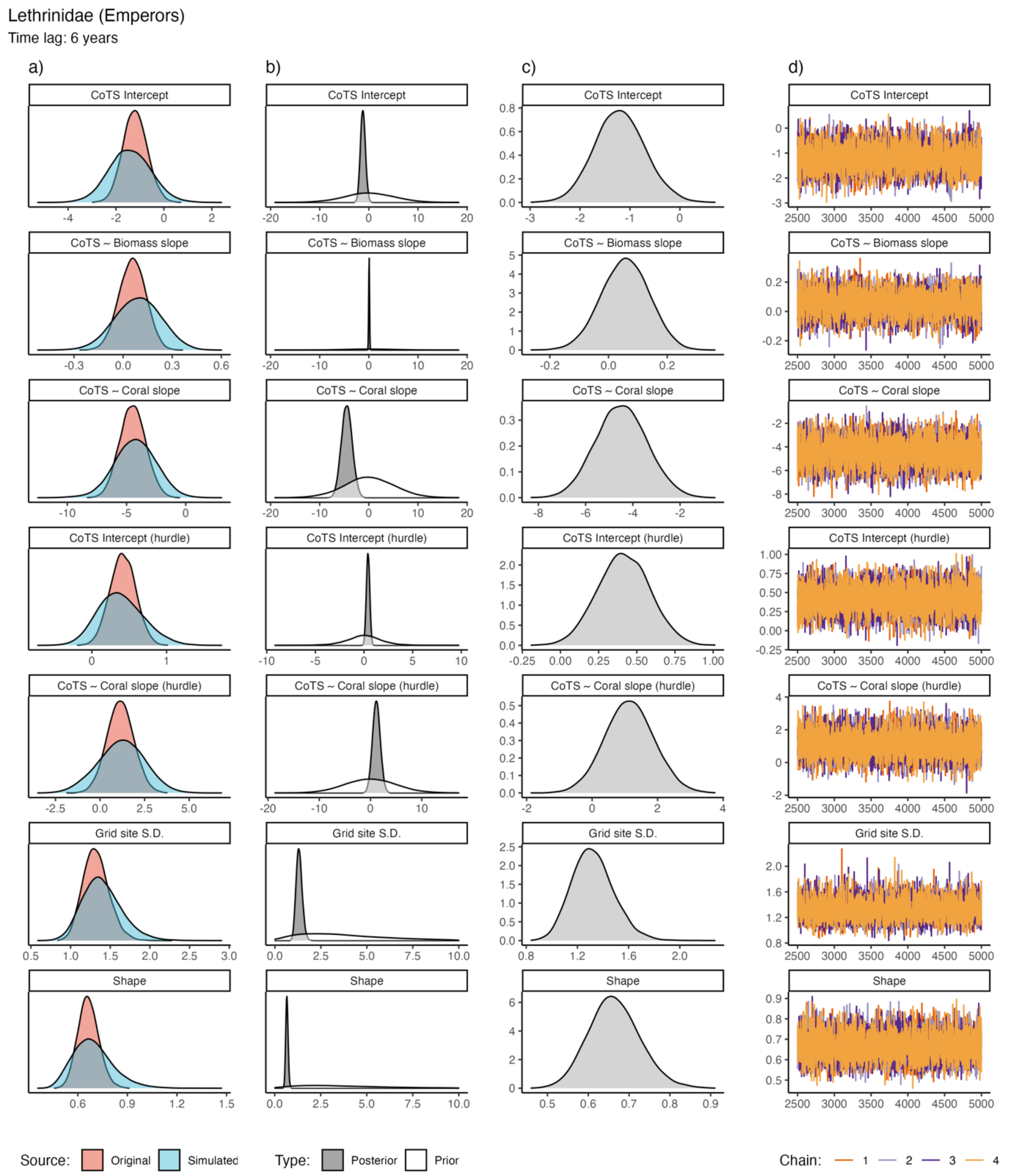

Supplementary Figure 28. Model validation checks.

a) Comparison between original posterior distributions of model parameters (red) on top of the combination of all posterior distributions across all 500 runs (blue, see Supplementary Method 2). b) Comparison between prior and posterior distribution of model parameters. c) Posterior distribution of model parameters. d) Chain mixing trace-plots.

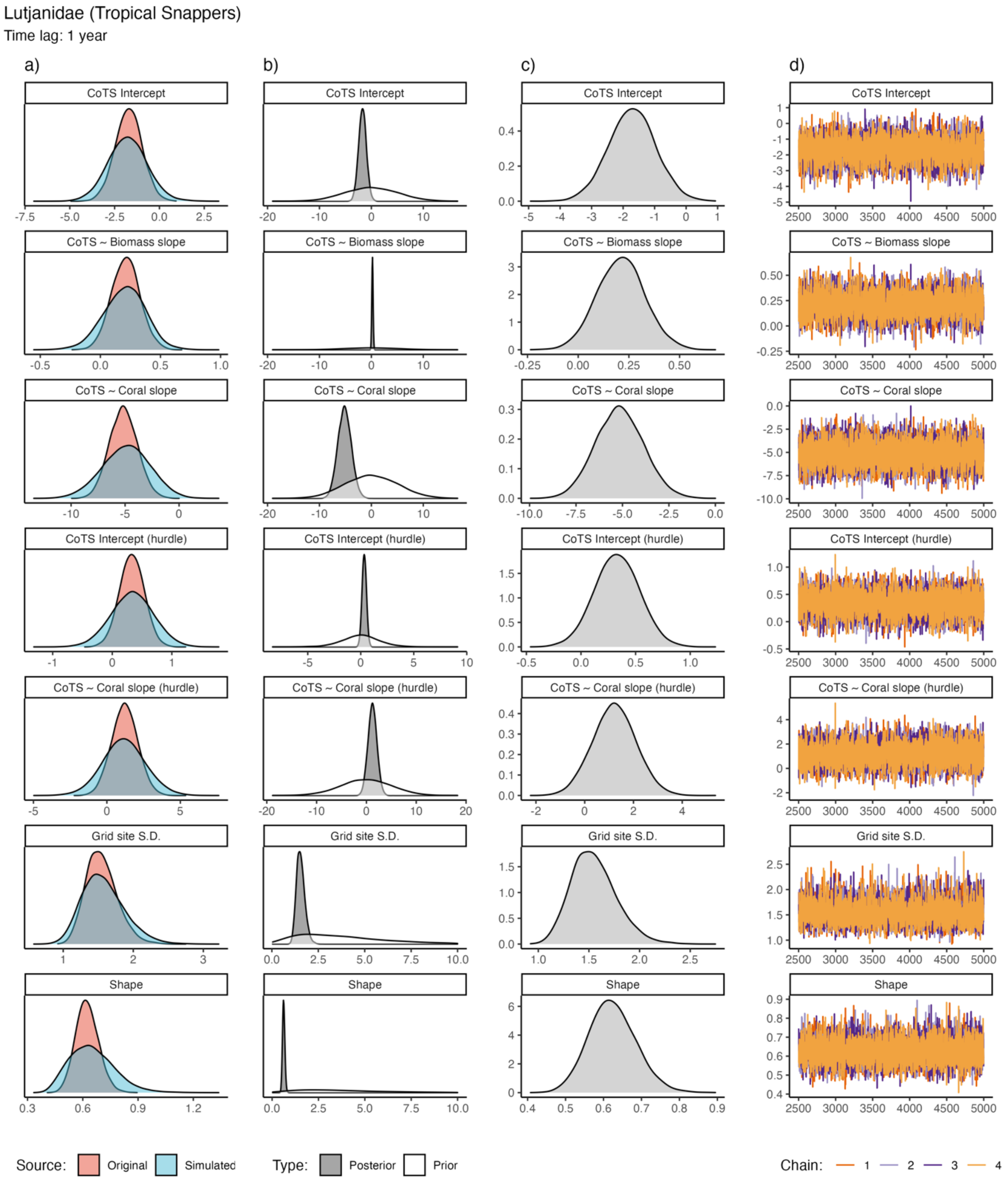

Supplementary Figure 29. Model validation checks.

a) Comparison between original posterior distributions of model parameters (red) on top of the combination of all posterior distributions across all 500 runs (blue, see Supplementary Method 2). b) Comparison between prior and posterior distribution of model parameters. c) Posterior distribution of model parameters. d) Chain mixing trace-plots.

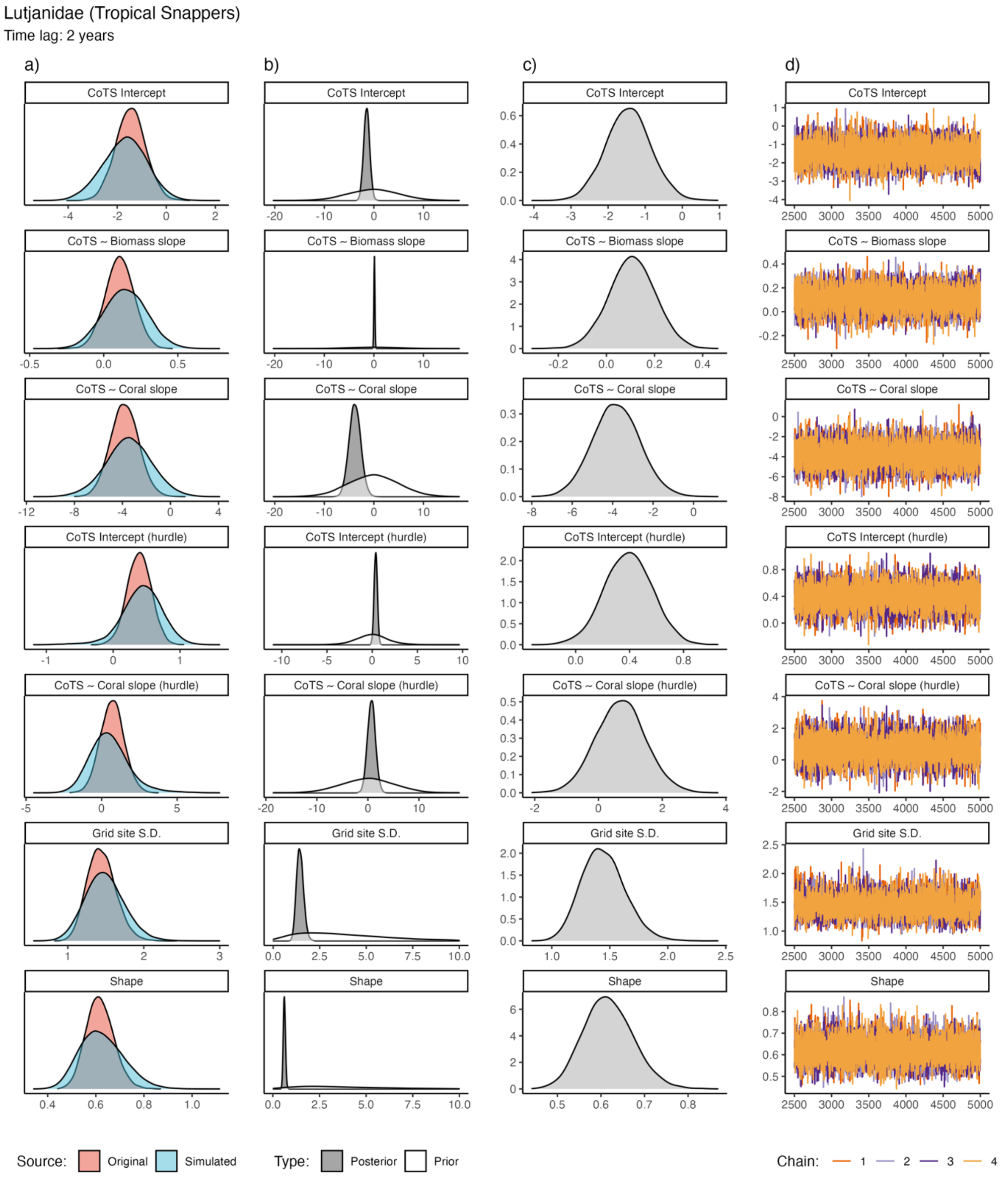

Supplementary Figure 30. Model validation checks.

a) Comparison between original posterior distributions of model parameters (red) on top of the combination of all posterior distributions across all 500 runs (blue, see Supplementary Method 2). b) Comparison between prior and posterior distribution of model parameters. c) Posterior distribution of model parameters. d) Chain mixing trace-plots.

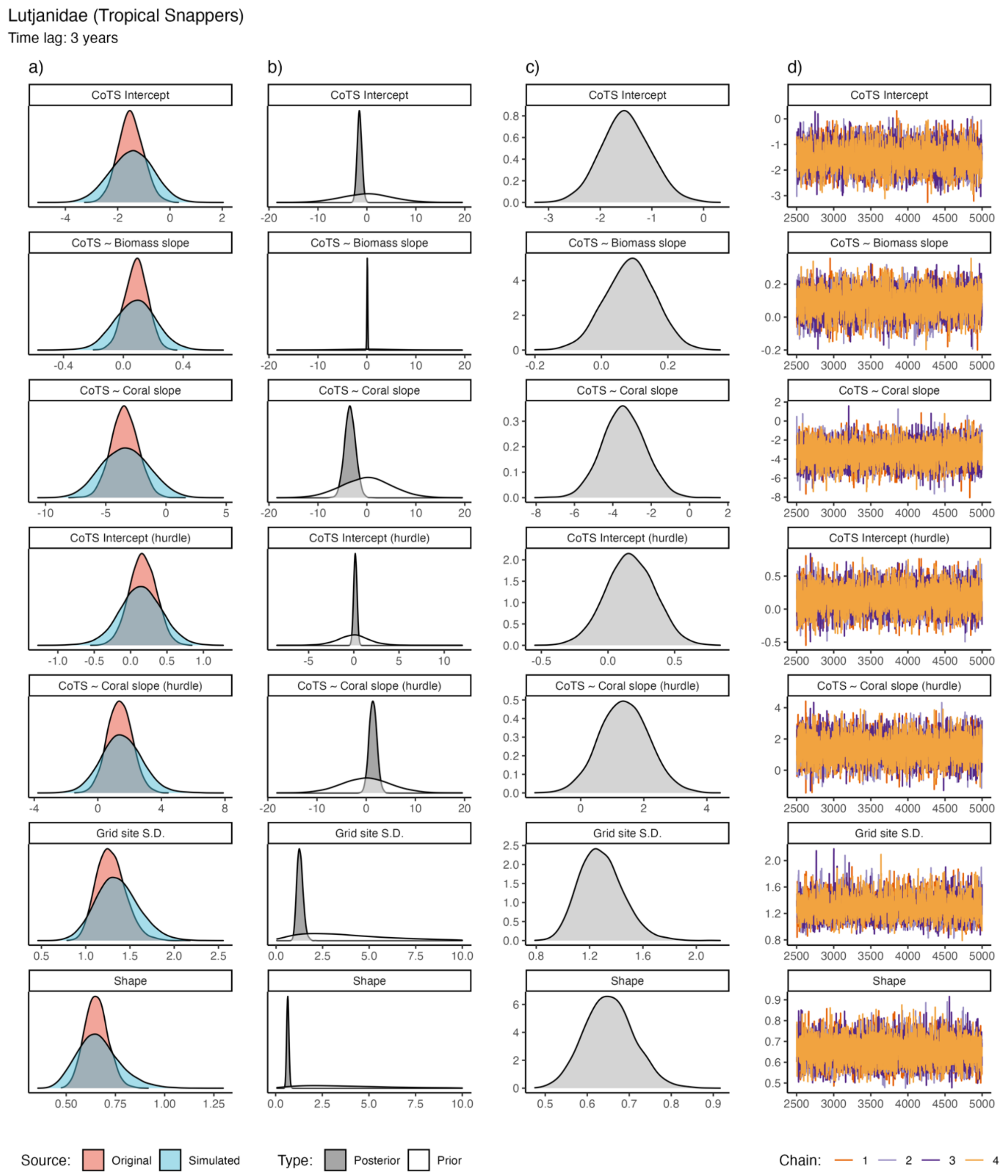

Supplementary Figure 31. Model validation checks.

a) Comparison between original posterior distributions of model parameters (red) on top of the combination of all posterior distributions across all 500 runs (blue, see Supplementary Method 2). b) Comparison between prior and posterior distribution of model parameters. c) Posterior distribution of model parameters. d) Chain mixing trace-plots.

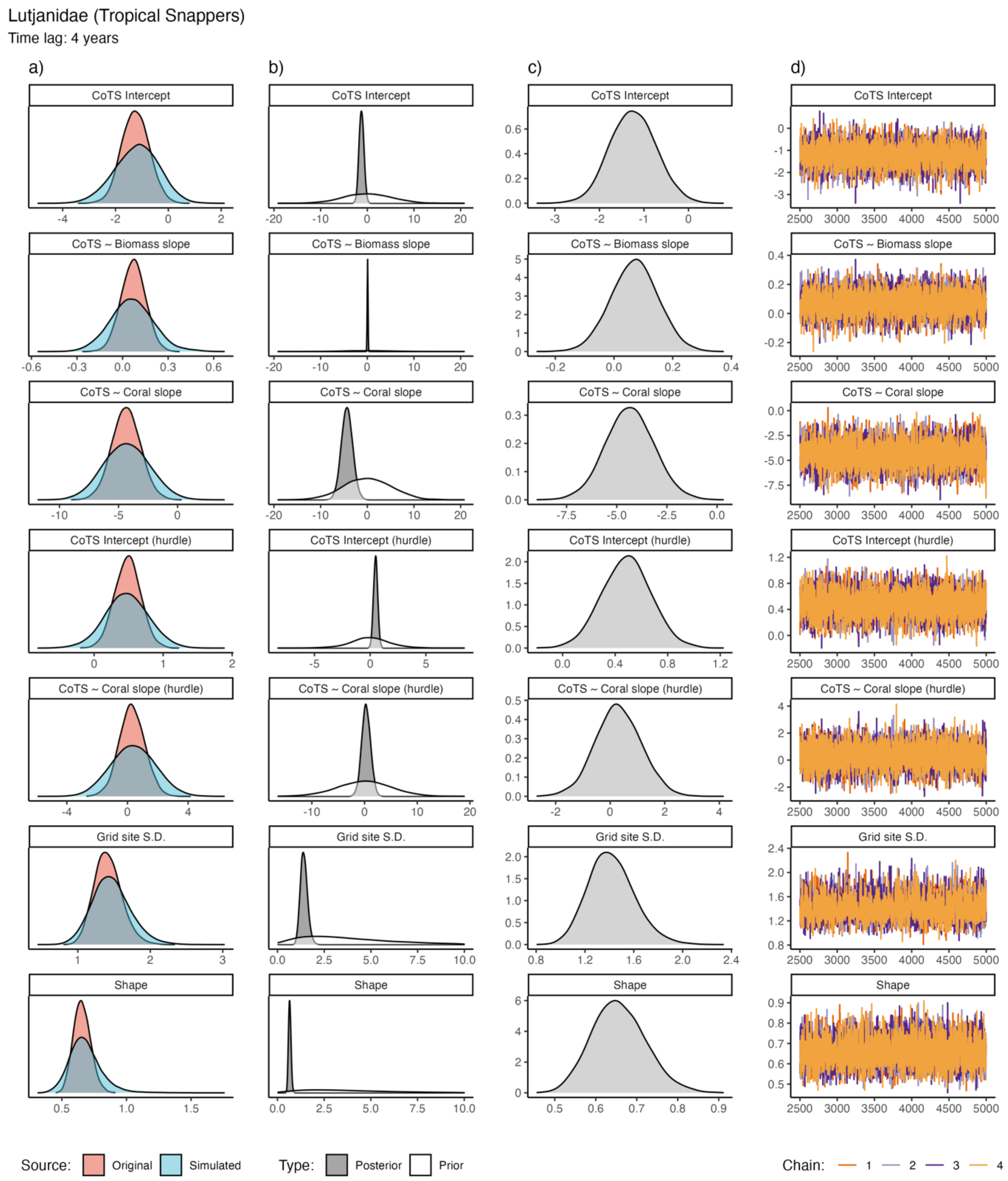

Supplementary Figure 32. Model validation checks.

a) Comparison between original posterior distributions of model parameters (red) on top of the combination of all posterior distributions across all 500 runs (blue, see Supplementary Method 2). b) Comparison between prior and posterior distribution of model parameters. c) Posterior distribution of model parameters. d) Chain mixing trace-plots.

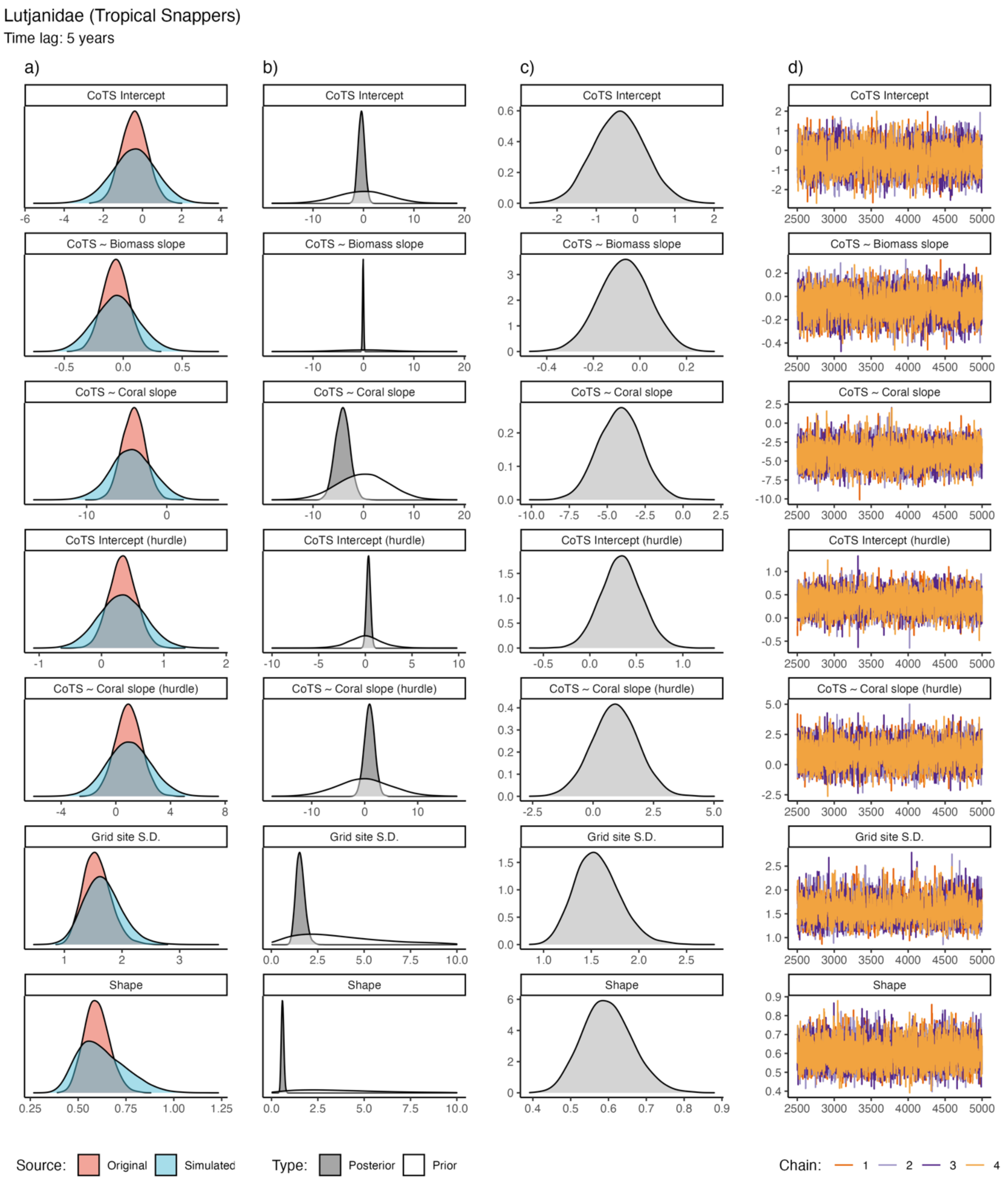

Supplementary Figure 33. Model validation checks.

a) Comparison between original posterior distributions of model parameters (red) on top of the combination of all posterior distributions across all 500 runs (blue, see Supplementary Method 2). b) Comparison between prior and posterior distribution of model parameters. c) Posterior distribution of model parameters. d) Chain mixing trace-plots.

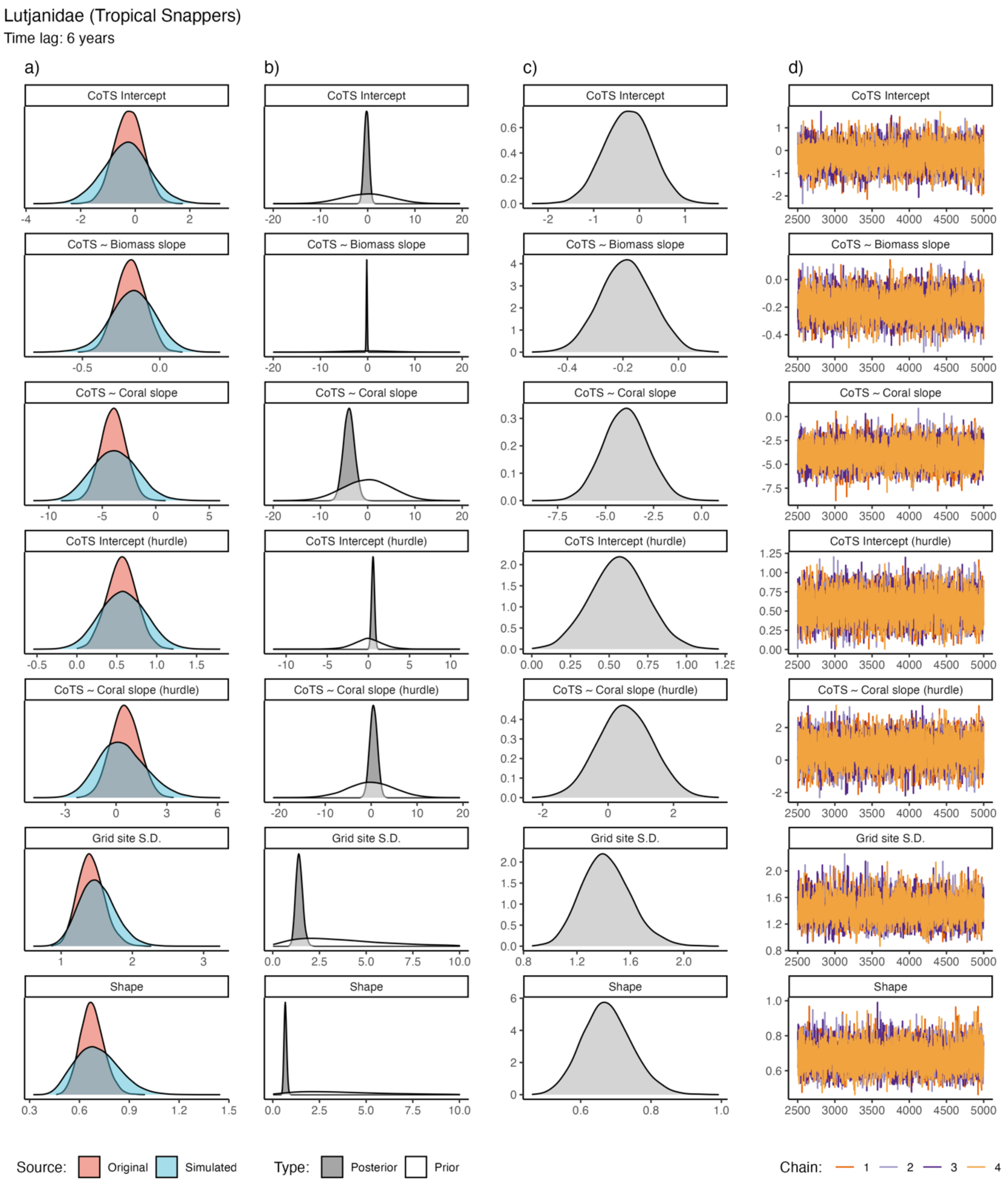

Supplementary Figure 34. Model validation checks.

a) Comparison between original posterior distributions of model parameters (red) on top of the combination of all posterior distributions across all 500 runs (blue, see Supplementary Method 2). b) Comparison between prior and posterior distribution of model parameters. c) Posterior distribution of model parameters. d) Chain mixing trace-plots.

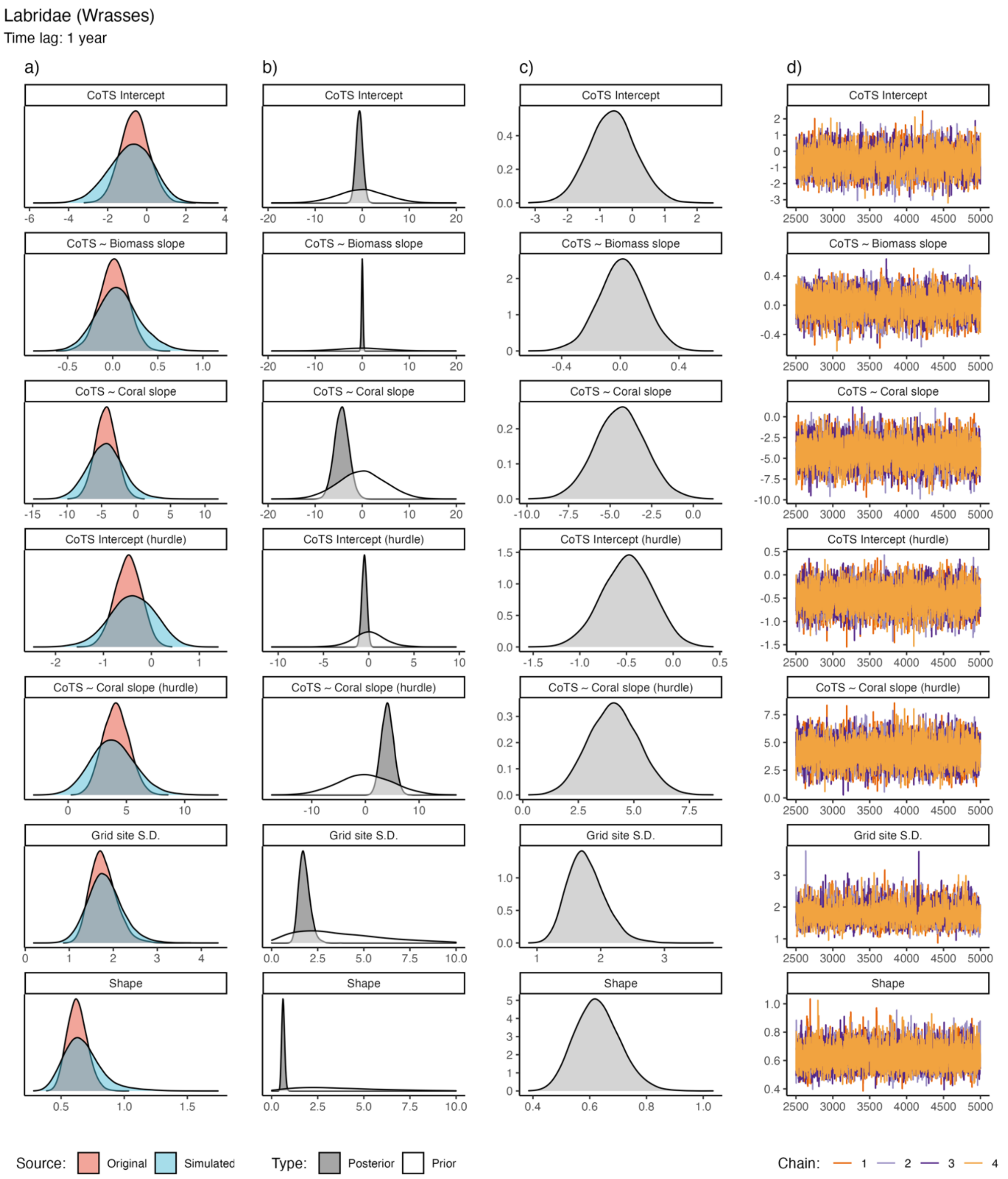

Supplementary Figure 35. Model validation checks.

a) Comparison between original posterior distributions of model parameters (red) on top of the combination of all posterior distributions across all 500 runs (blue, see Supplementary Method 2). b) Comparison between prior and posterior distribution of model parameters. c) Posterior distribution of model parameters. d) Chain mixing trace-plots.

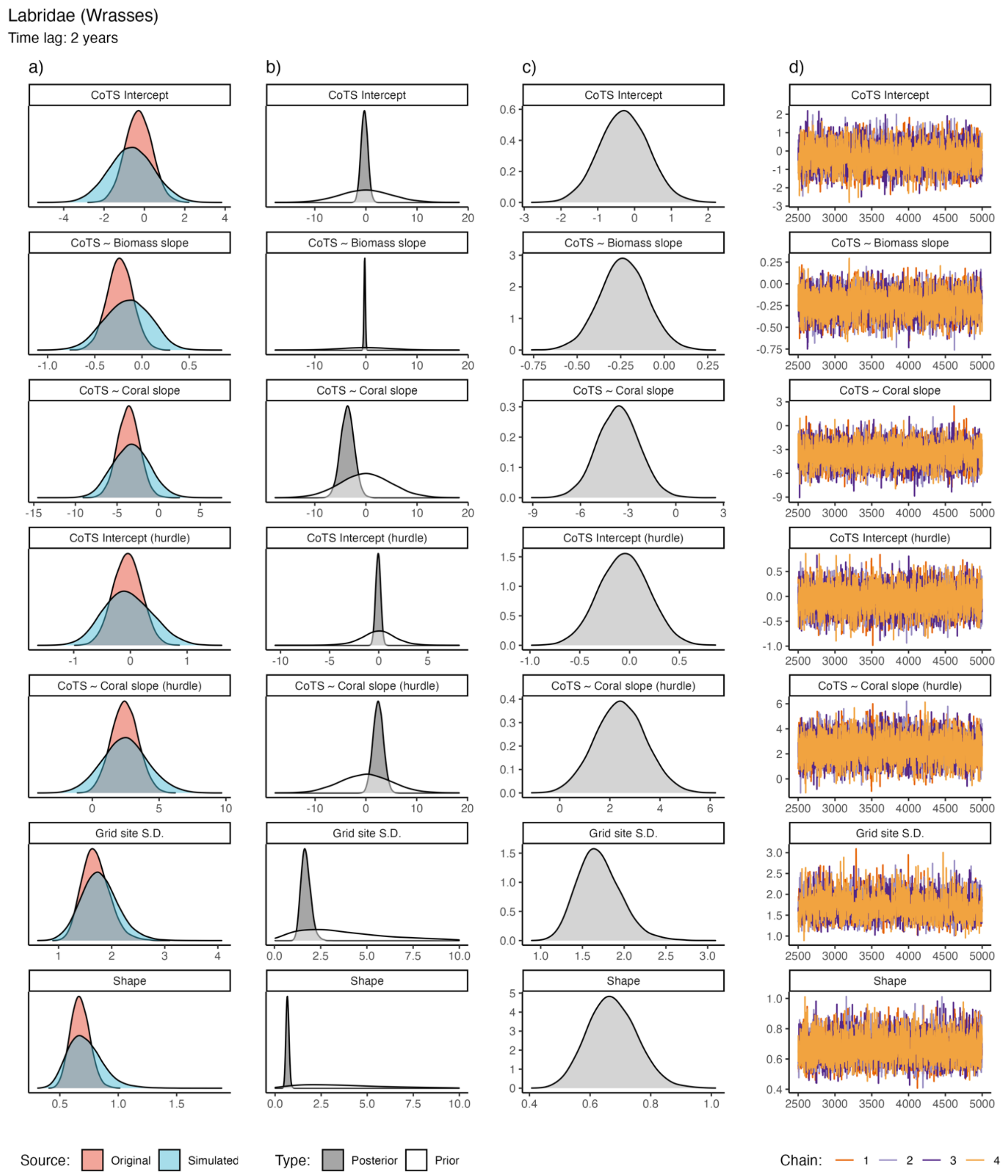

Supplementary Figure 36. Model validation checks.

a) Comparison between original posterior distributions of model parameters (red) on top of the combination of all posterior distributions across all 500 runs (blue, see Supplementary Method 2). b) Comparison between prior and posterior distribution of model parameters. c) Posterior distribution of model parameters. d) Chain mixing trace-plots.

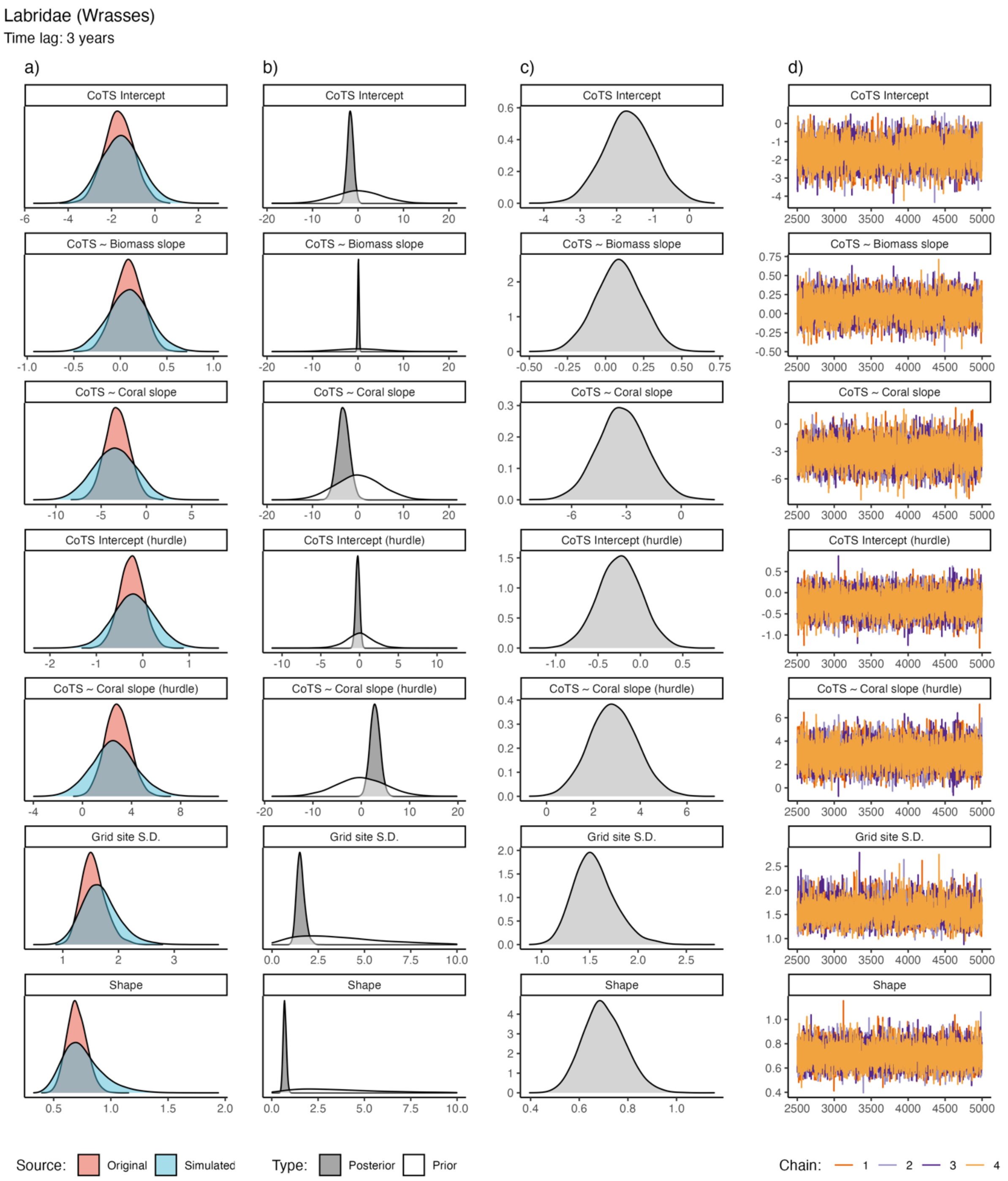

Supplementary Figure 37. Model validation checks.

a) Comparison between original posterior distributions of model parameters (red) on top of the combination of all posterior distributions across all 500 runs (blue, see Supplementary Method 2). b) Comparison between prior and posterior distribution of model parameters. c) Posterior distribution of model parameters. d) Chain mixing trace-plots.

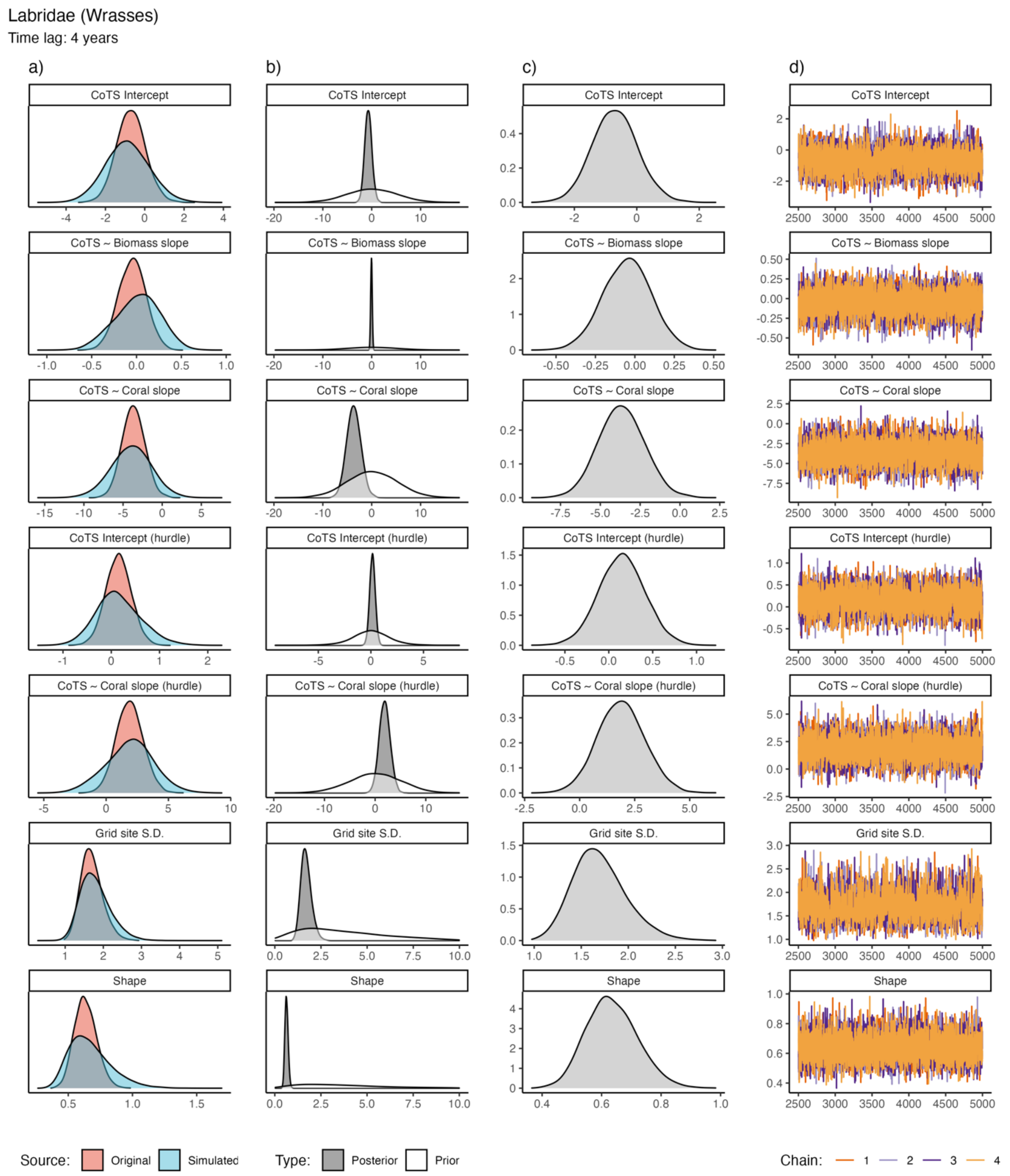

Supplementary Figure 38. Model validation checks.

a) Comparison between original posterior distributions of model parameters (red) on top of the combination of all posterior distributions across all 500 runs (blue, see Supplementary Method 2). b) Comparison between prior and posterior distribution of model parameters. c) Posterior distribution of model parameters. d) Chain mixing trace-plots.

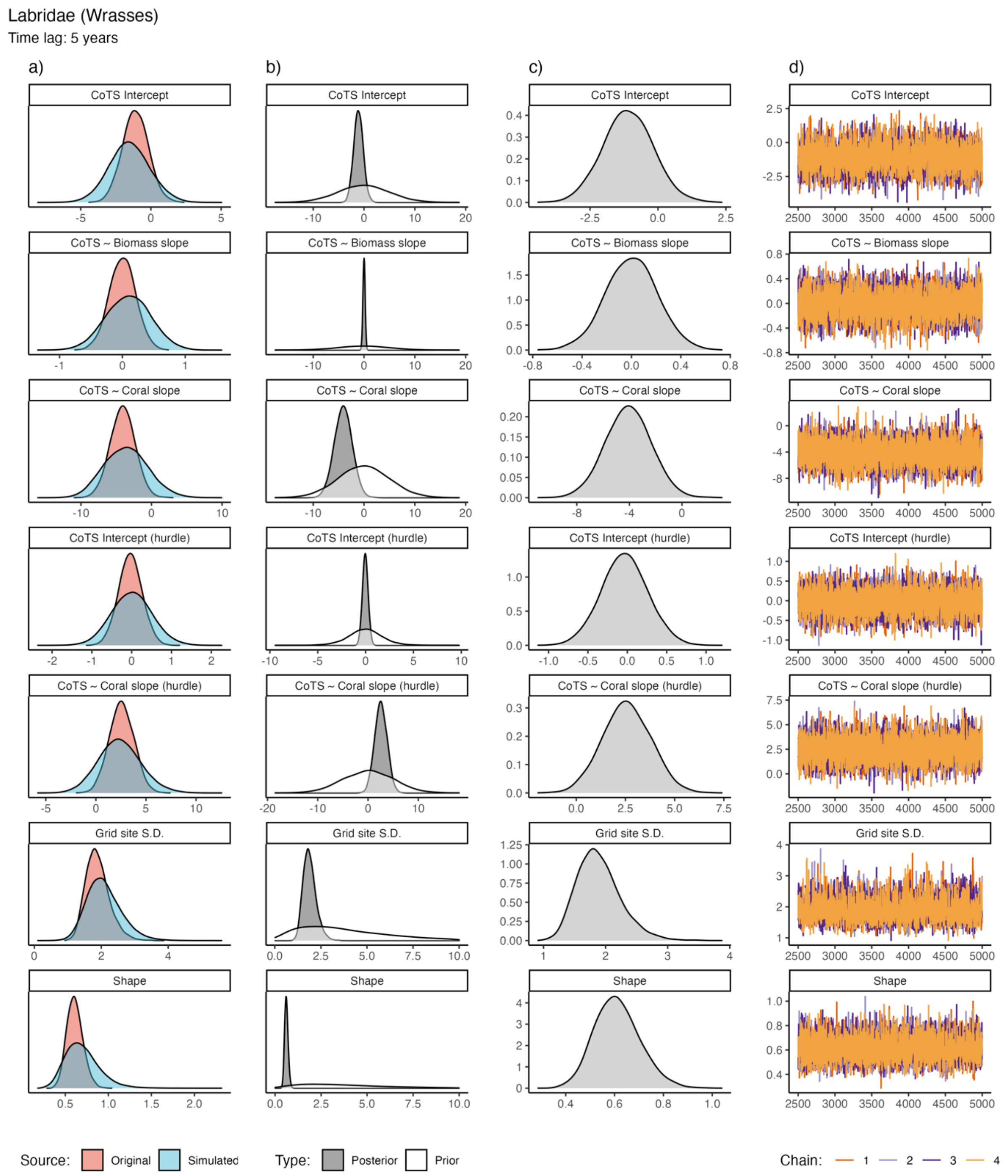

Supplementary Figure 39. Model validation checks.

a) Comparison between original posterior distributions of model parameters (red) on top of the combination of all posterior distributions across all 500 runs (blue, see Supplementary Method 2). b) Comparison between prior and posterior distribution of model parameters. c) Posterior distribution of model parameters. d) Chain mixing trace-plots.

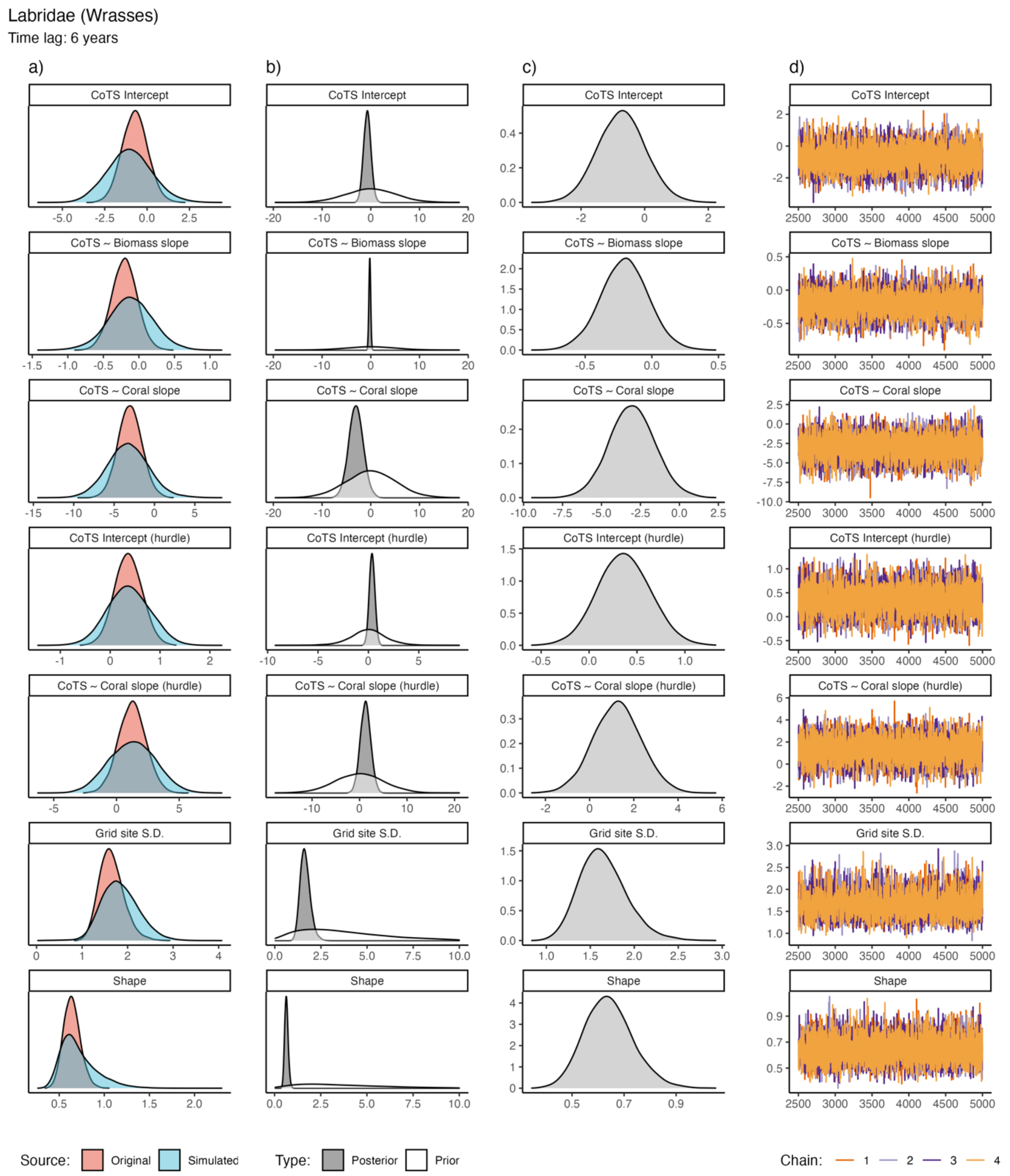

Supplementary Figure 40. Model validation checks.

a) Comparison between original posterior distributions of model parameters (red) on top of the combination of all posterior distributions across all 500 runs (blue, see Supplementary Method 2). b) Comparison between prior and posterior distribution of model parameters. c) Posterior distribution of model parameters. d) Chain mixing trace-plots.

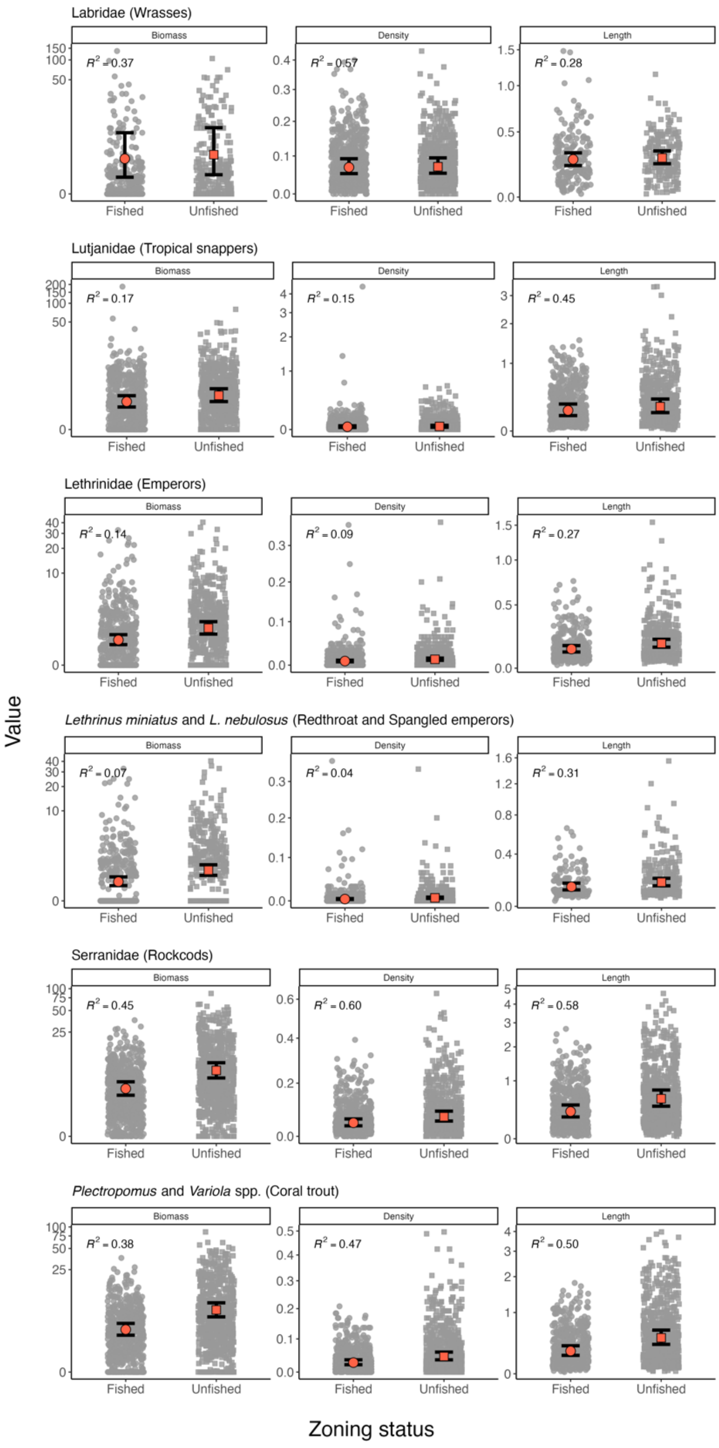

Supplementary Figure 41. The effects of zoning on coral reef fish biomass, density and length.

Presented are actual data (grey points), the mean (red points) and 95% Bayesian credible intervals (error bars) for fish biomass, density and length, obtained from all surveys

conducted on 56 paired fished and unfished reefs since the re-zoning of the Great Barrier Reef Marine Park, Australia, in 2004. The y axis has been scaled to a log + 1 for visualisation purposes. Results are presented from top to bottom for six fish groups, namely (1) Labridae (wrasses), (2) Lethrinidae (emperors), (3) *Lethrinus miniatus* and *L. nebulosus* (redthroat and spangled emperors), (4) Lutjanidae (tropical snappers), (5) Serranidae (rockcods) and (6) *Plectropomus* spp. and *Variola* spp. (coral trout). See model description in the Supplementary Method 3.

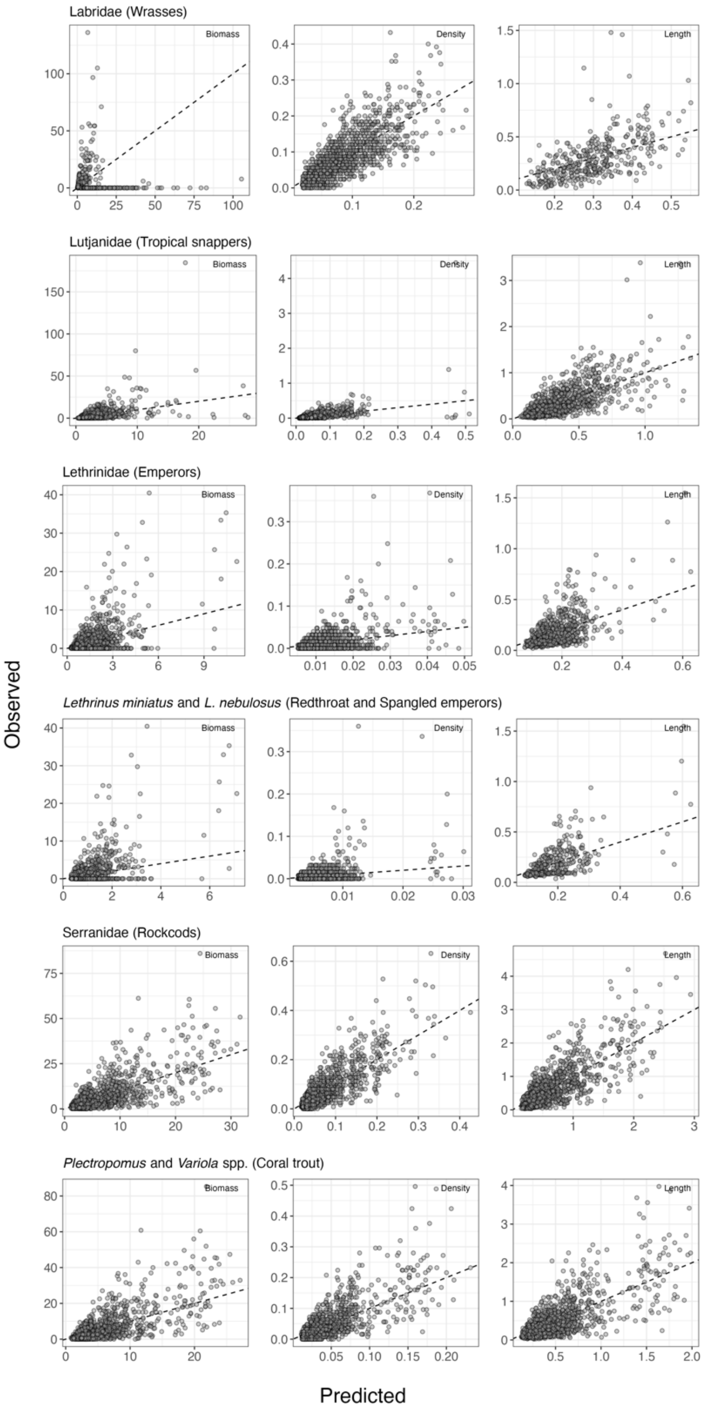

Supplementary Figure 42. Posterior predictive check of the effects of zoning on coral reef fish biomass, density and length.

Observed values on the y axis, and observation-level mean posterior prediction on the x axis. Dashed line represents a 1-to-1 fit. See model description in the Supplementary Method 3.

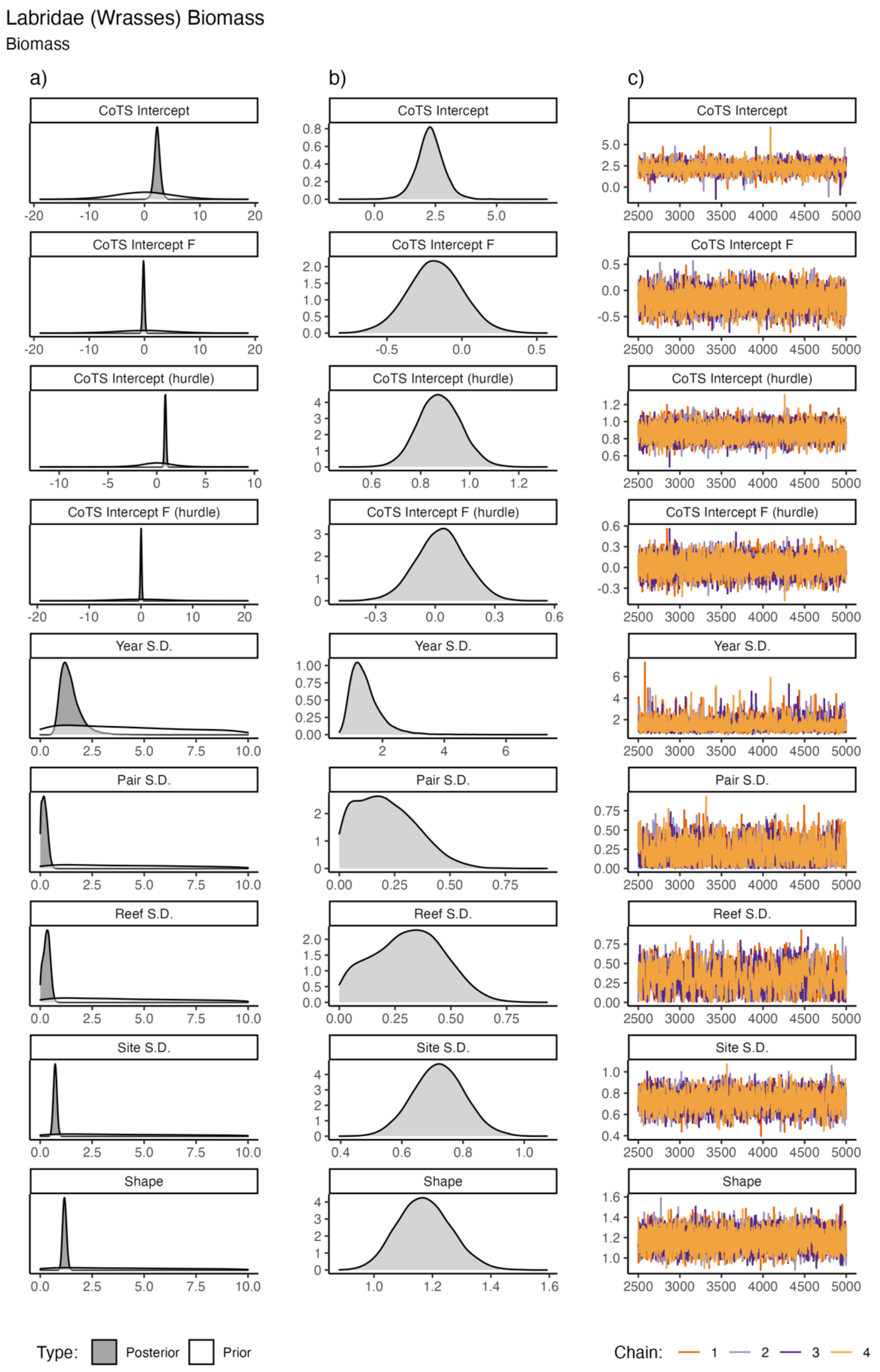

Supplementary Figure 43. Model validation checks.

a) Comparison between prior and posterior distribution of model parameters. b) Posterior distribution of model parameters. c) Chain mixing trace-plots.

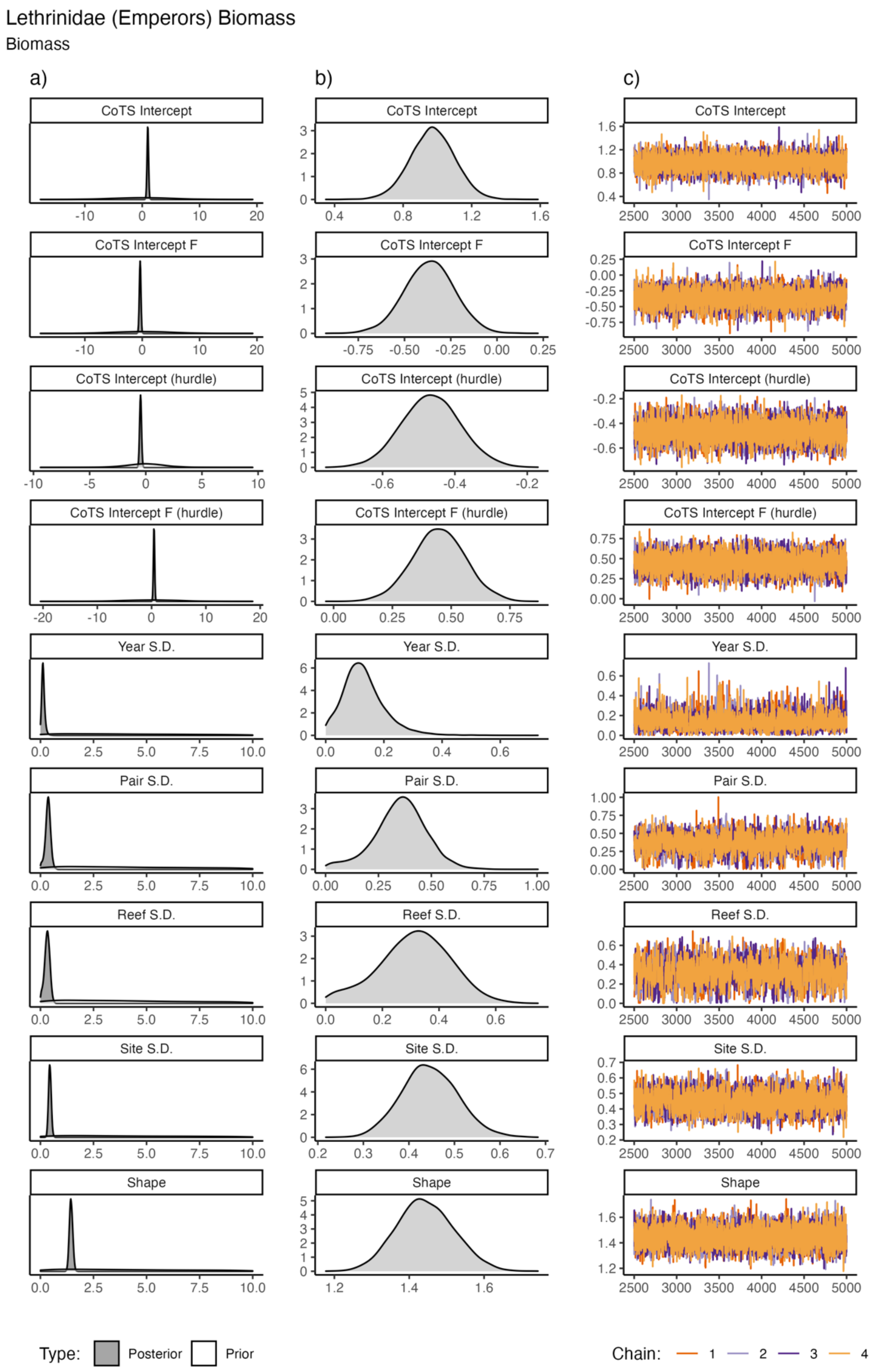

Supplementary Figure 44. Model validation checks.

a) Comparison between prior and posterior distribution of model parameters. b) Posterior distribution of model parameters. c) Chain mixing trace-plots.

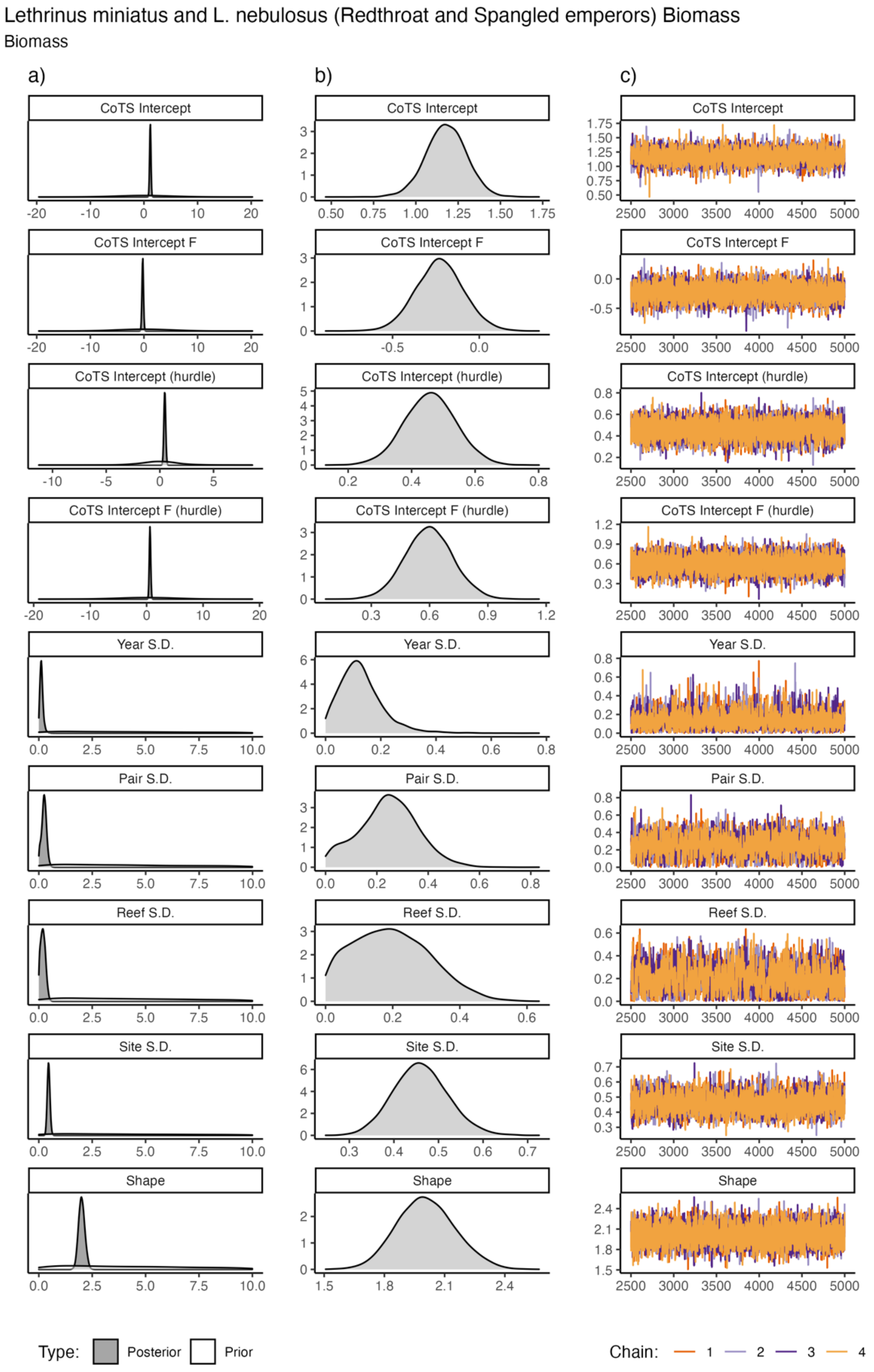

Supplementary Figure 45. Model validation checks.

a) Comparison between prior and posterior distribution of model parameters. b) Posterior distribution of model parameters. c) Chain mixing trace-plots.

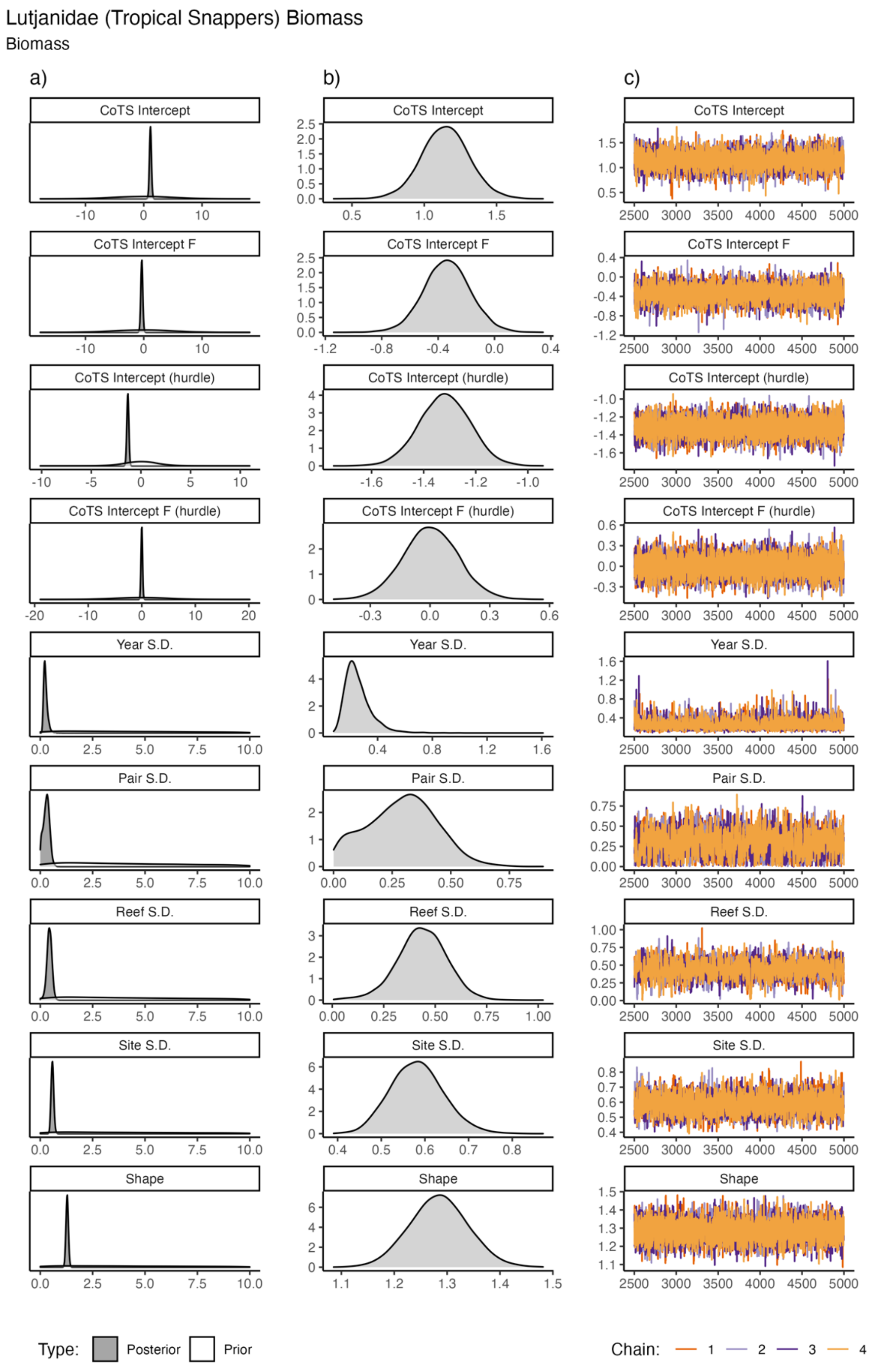

Supplementary Figure 46. Model validation checks.

a) Comparison between prior and posterior distribution of model parameters. b) Posterior distribution of model parameters. c) Chain mixing trace-plots.

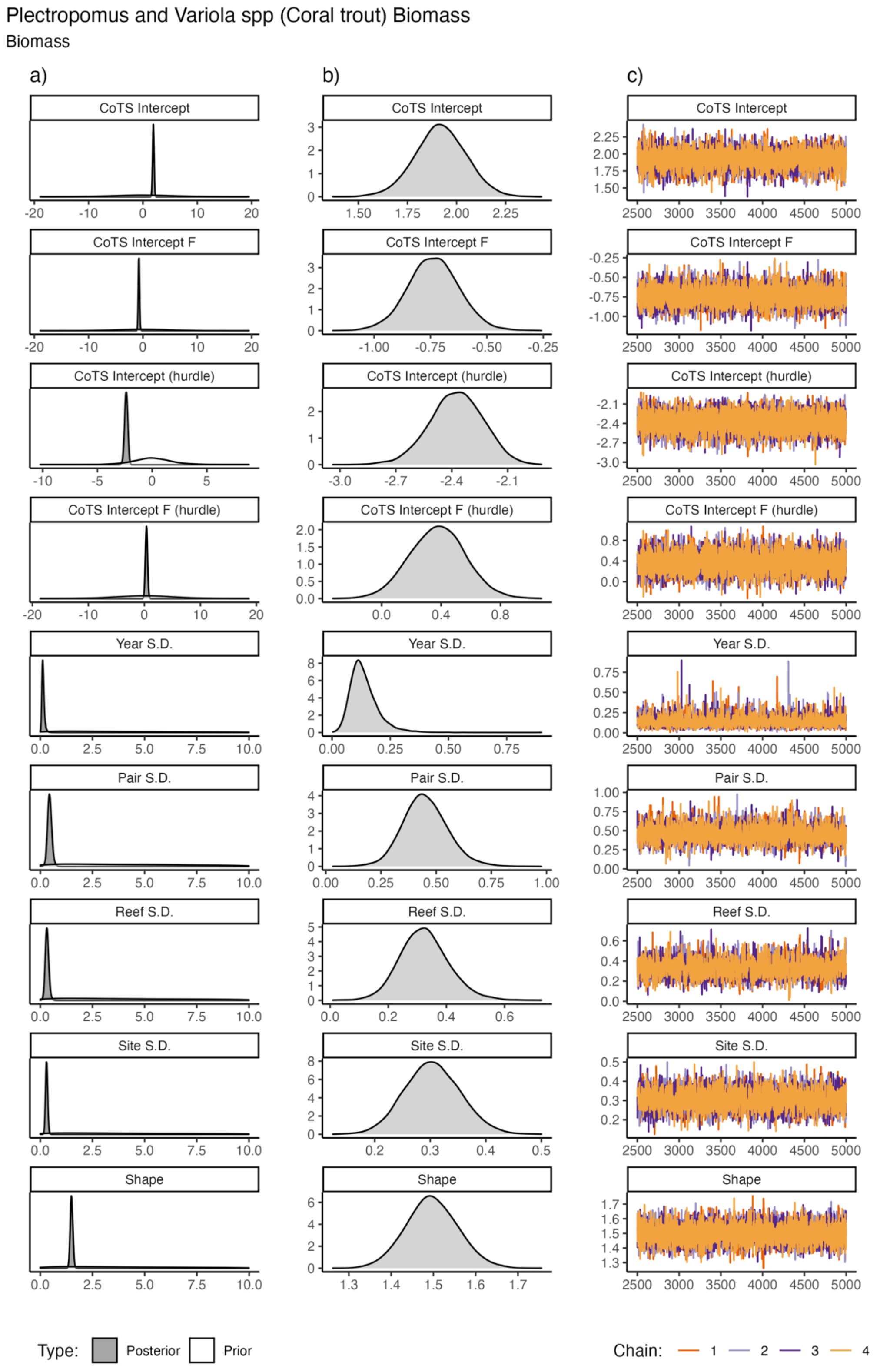

Supplementary Figure 47. Model validation checks.

a) Comparison between prior and posterior distribution of model parameters. b) Posterior distribution of model parameters. c) Chain mixing trace-plots.

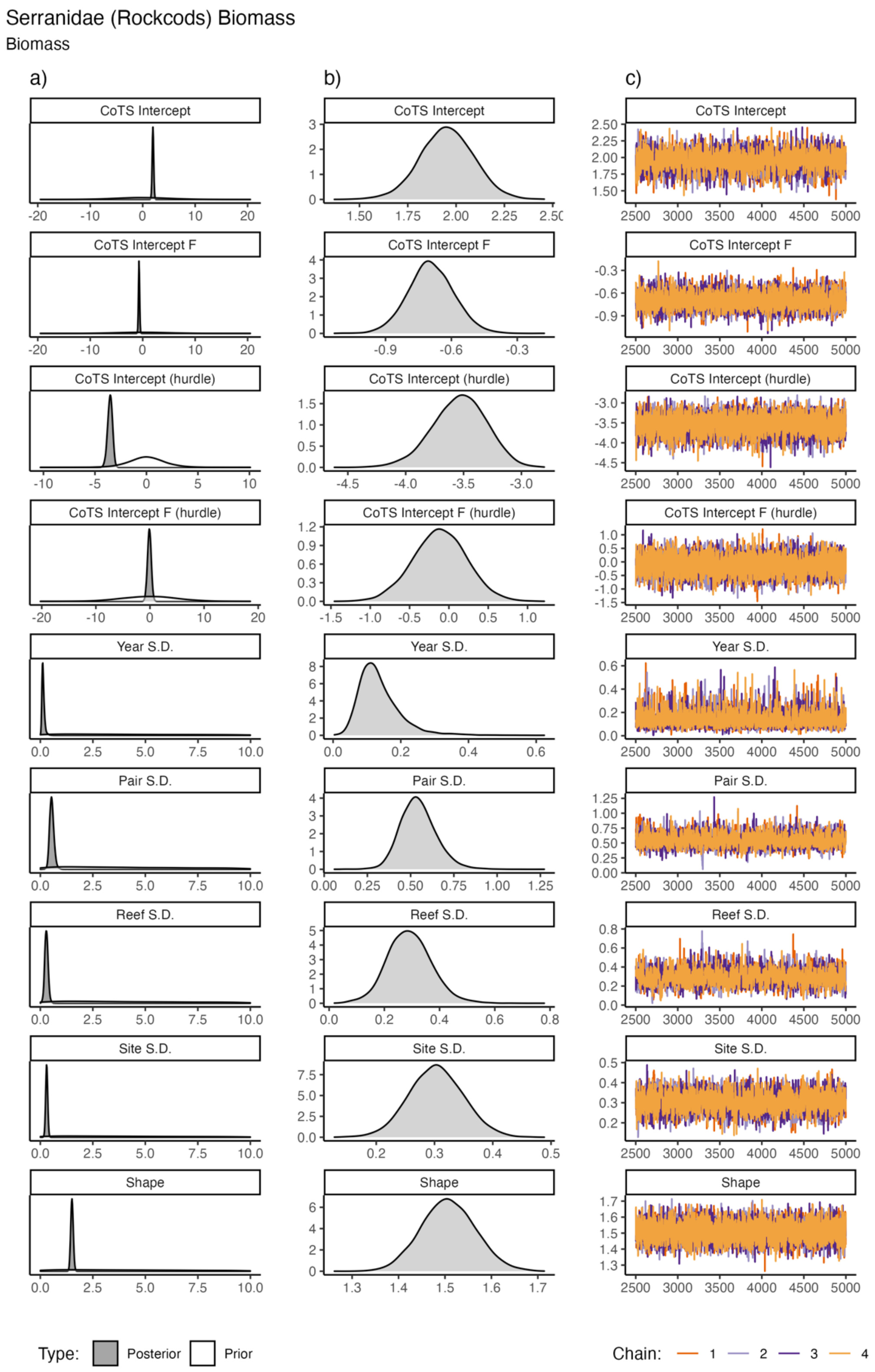

Supplementary Figure 48. Model validation checks.

a) Comparison between prior and posterior distribution of model parameters. b) Posterior distribution of model parameters. c) Chain mixing trace-plots.

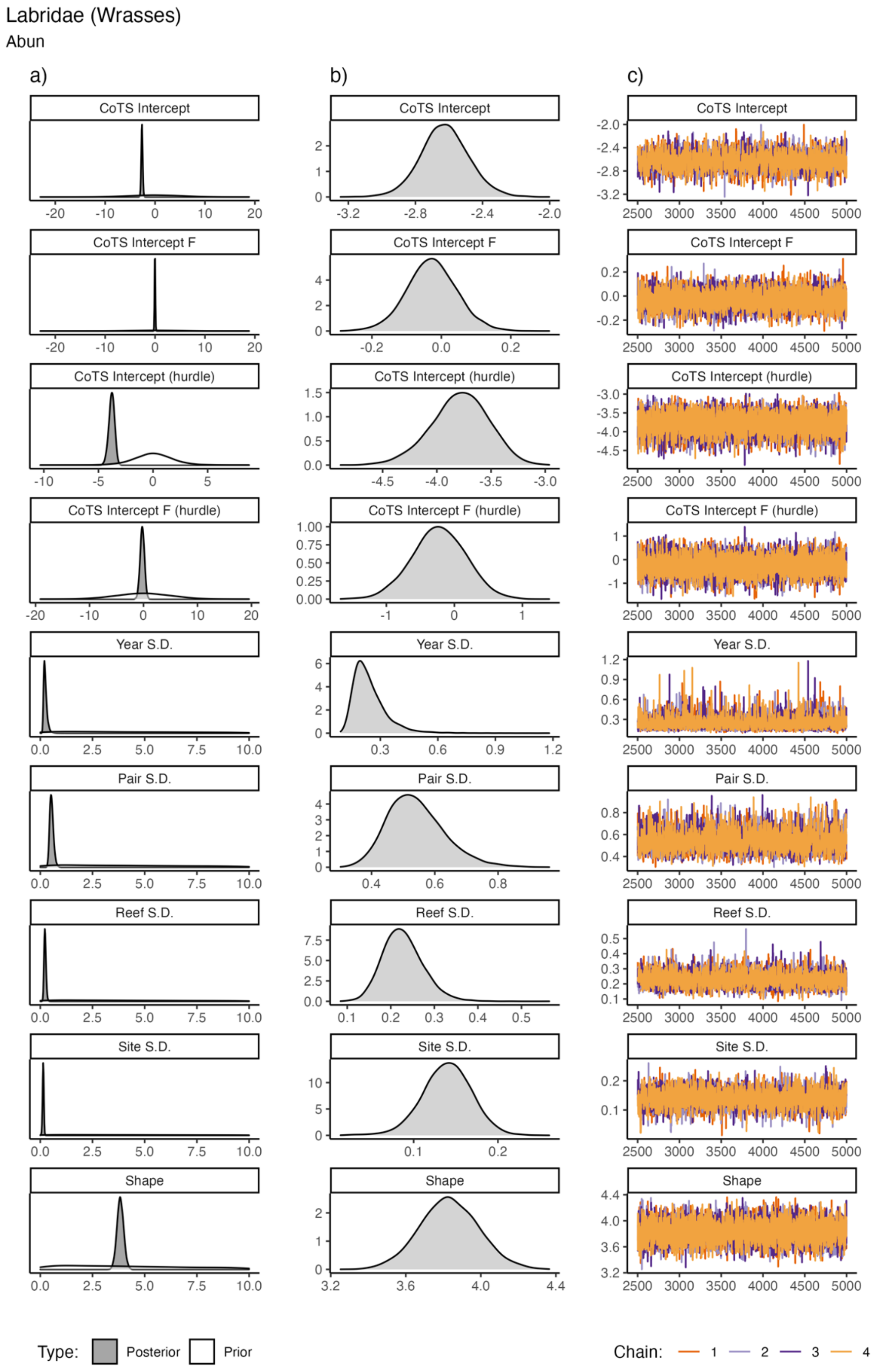

Supplementary Figure 49. Model validation checks.

a) Comparison between prior and posterior distribution of model parameters. b) Posterior distribution of model parameters. c) Chain mixing trace-plots.

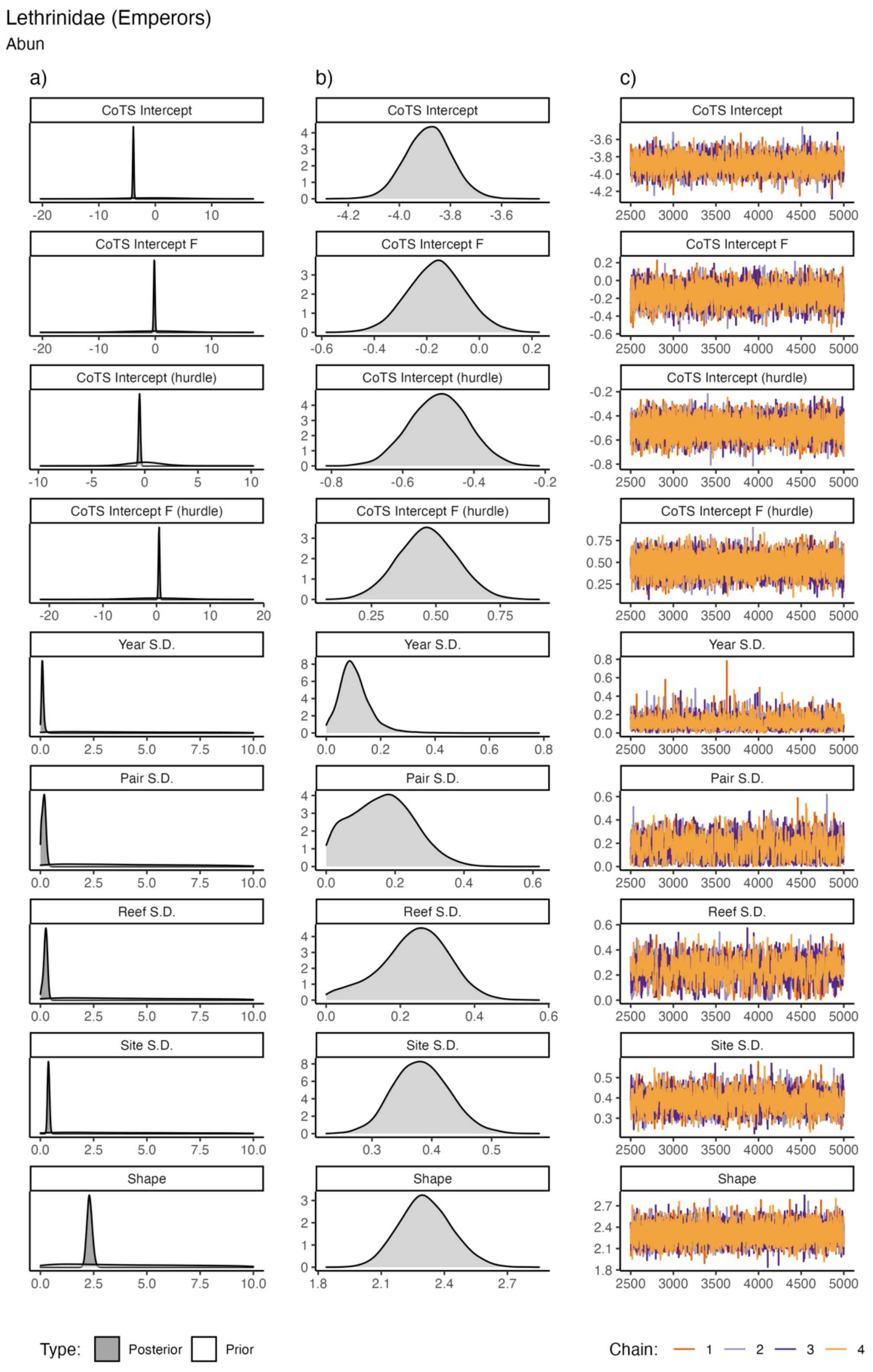

Supplementary Figure 50. Model validation checks.

a) Comparison between prior and posterior distribution of model parameters. b) Posterior distribution of model parameters. c) Chain mixing trace-plots.

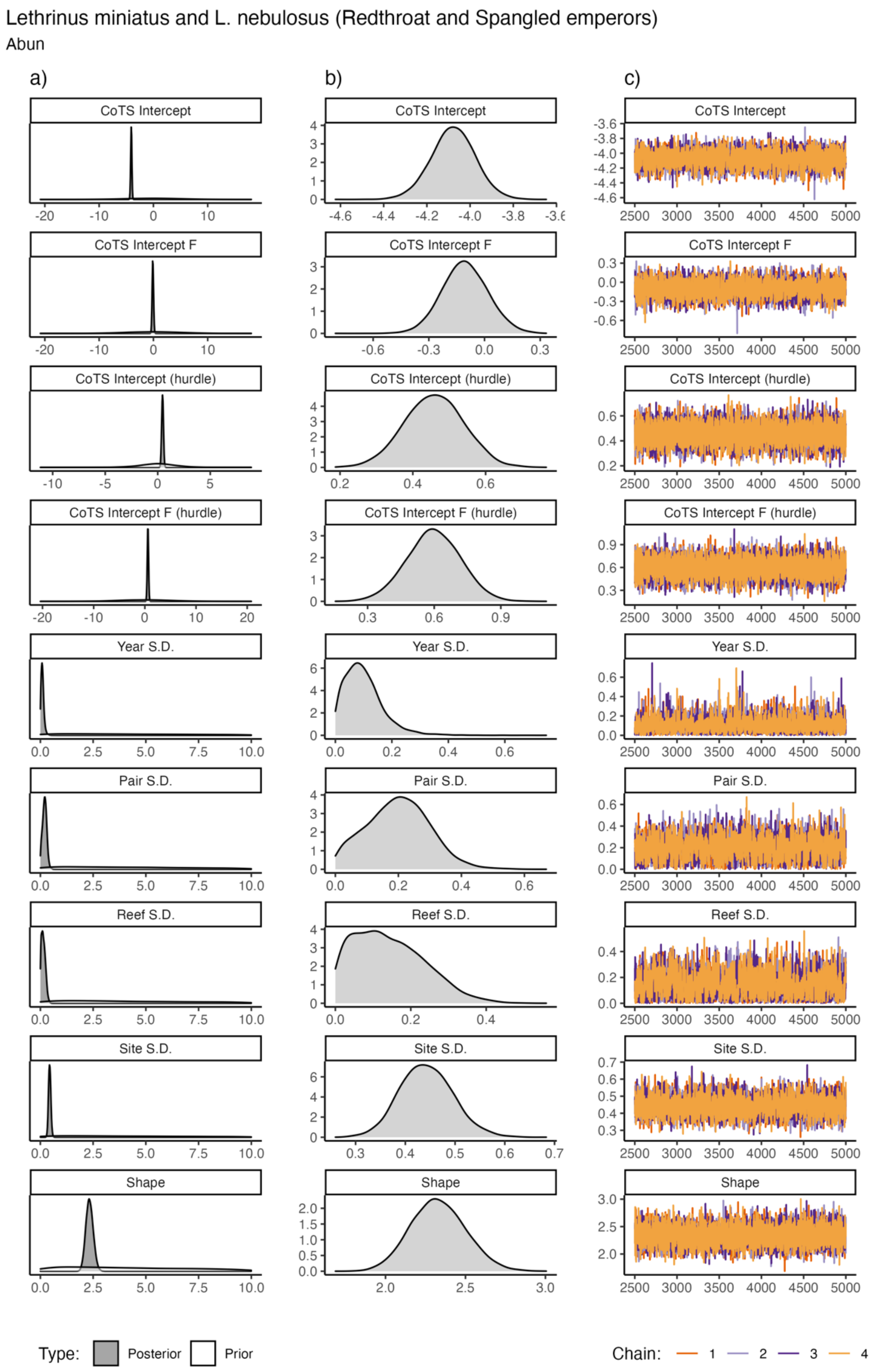

Supplementary Figure 51. Model validation checks.

a) Comparison between prior and posterior distribution of model parameters. b) Posterior distribution of model parameters. c) Chain mixing trace-plots.

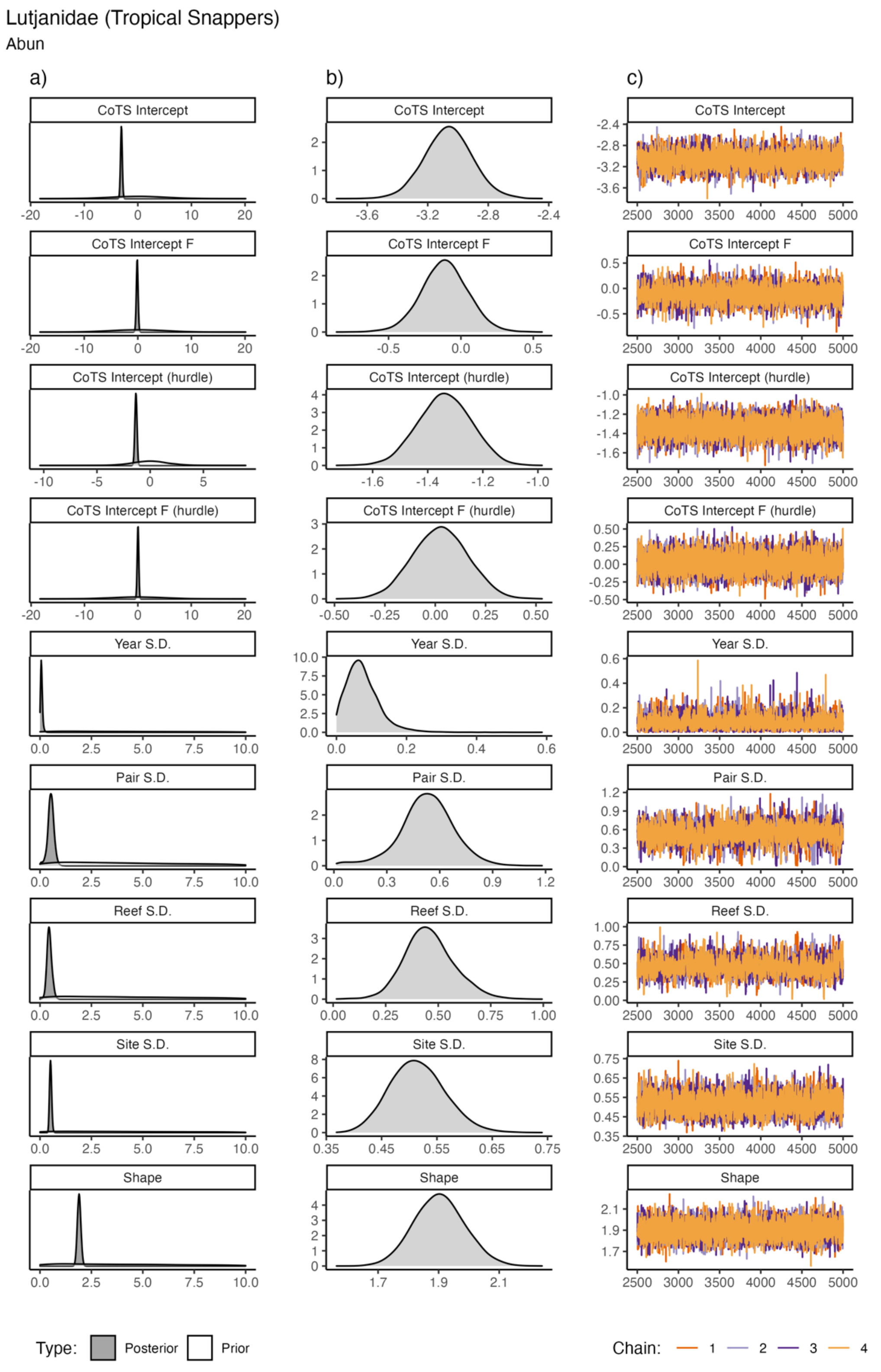

Supplementary Figure 52. Model validation checks.

a) Comparison between prior and posterior distribution of model parameters. b) Posterior distribution of model parameters. c) Chain mixing trace-plots.

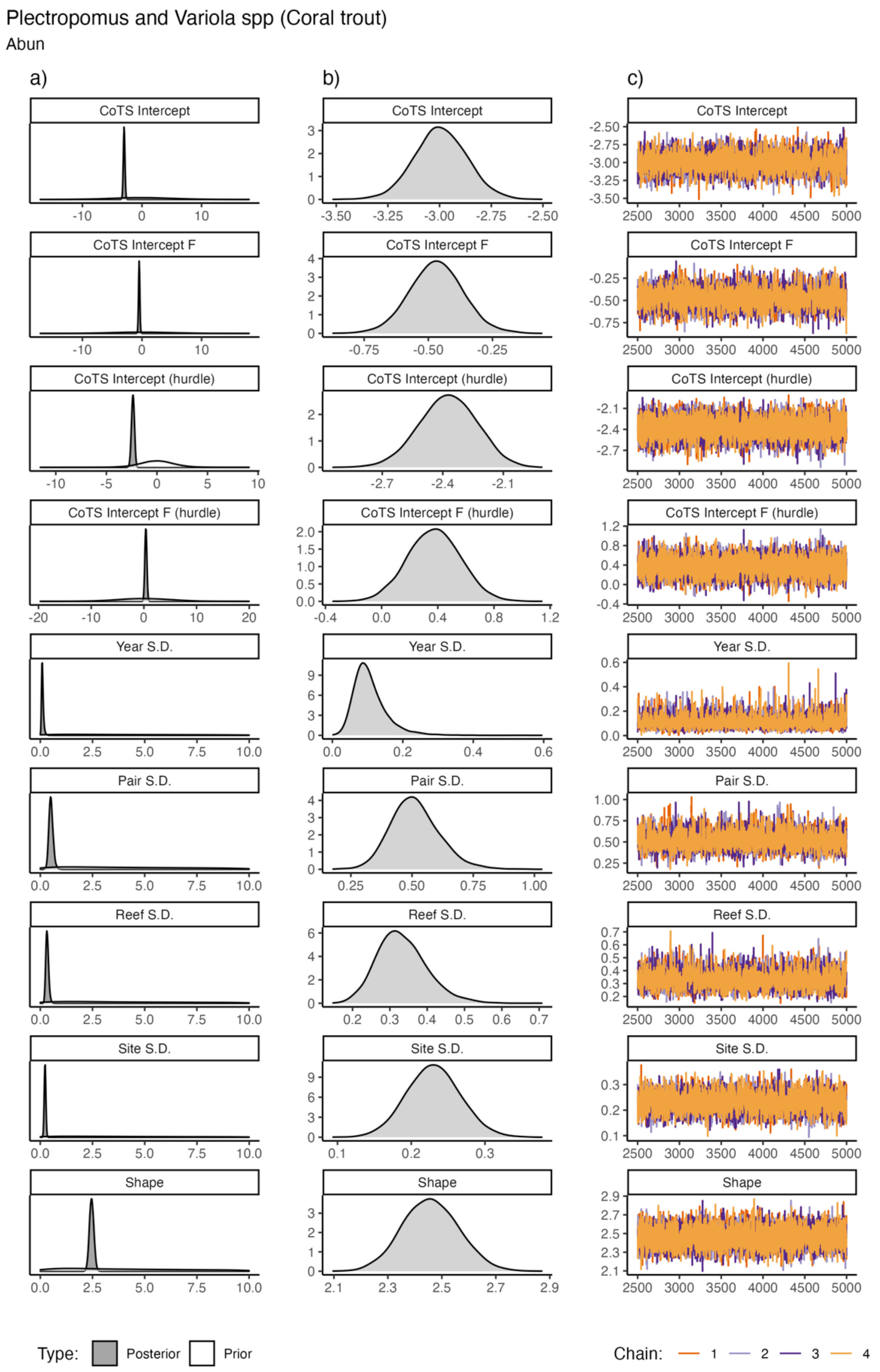

Supplementary Figure 53. Model validation checks.

a) Comparison between prior and posterior distribution of model parameters. b) Posterior distribution of model parameters. c) Chain mixing trace-plots.

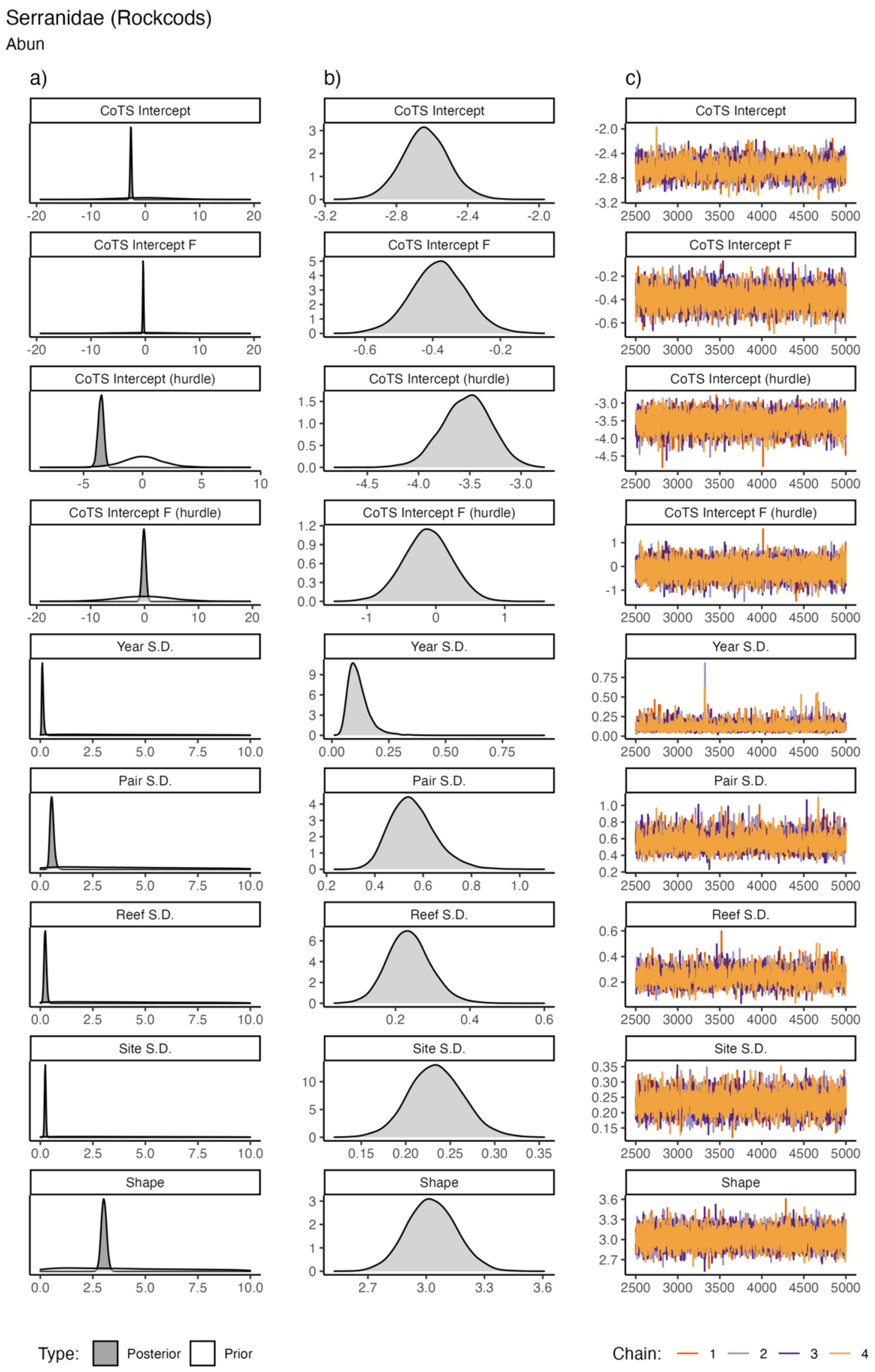

Supplementary Figure 54. Model validation checks.

a) Comparison between prior and posterior distribution of model parameters. b) Posterior distribution of model parameters. c) Chain mixing trace-plots.

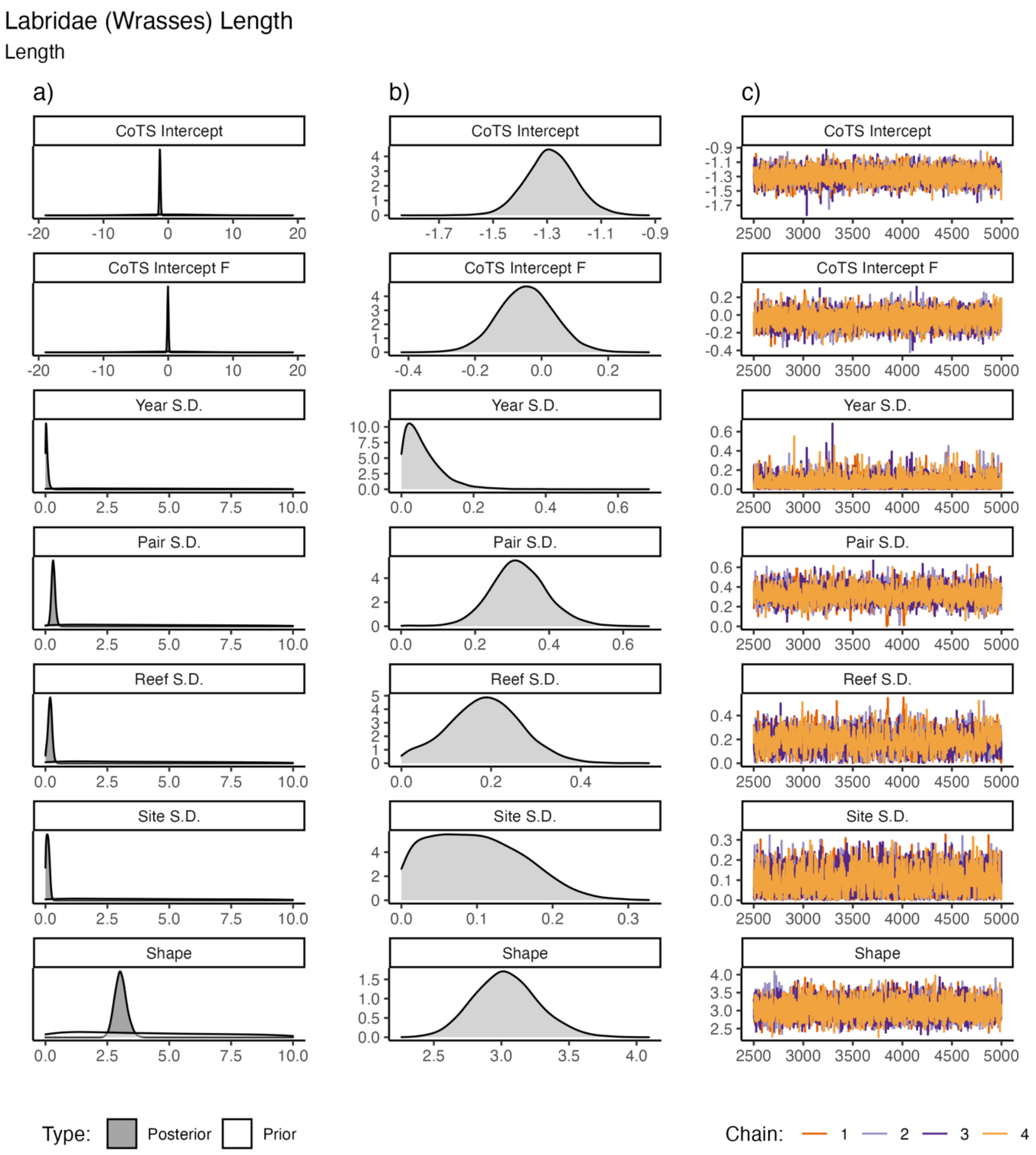

Supplementary Figure 55. Model validation checks.

a) Comparison between prior and posterior distribution of model parameters. b) Posterior distribution of model parameters. c) Chain mixing trace-plots.

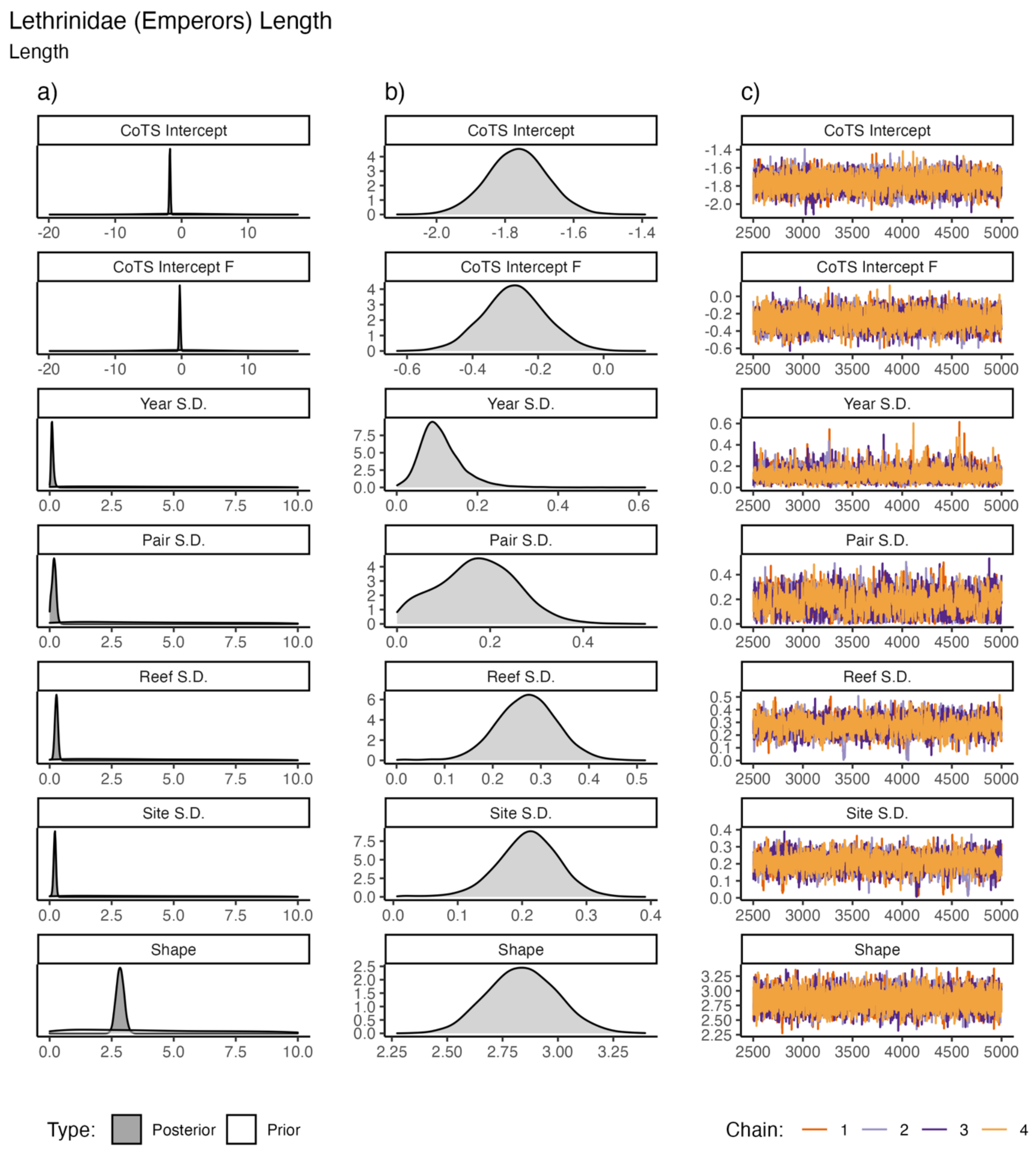

Supplementary Figure 56. Model validation checks.

a) Comparison between prior and posterior distribution of model parameters. b) Posterior distribution of model parameters. c) Chain mixing trace-plots.

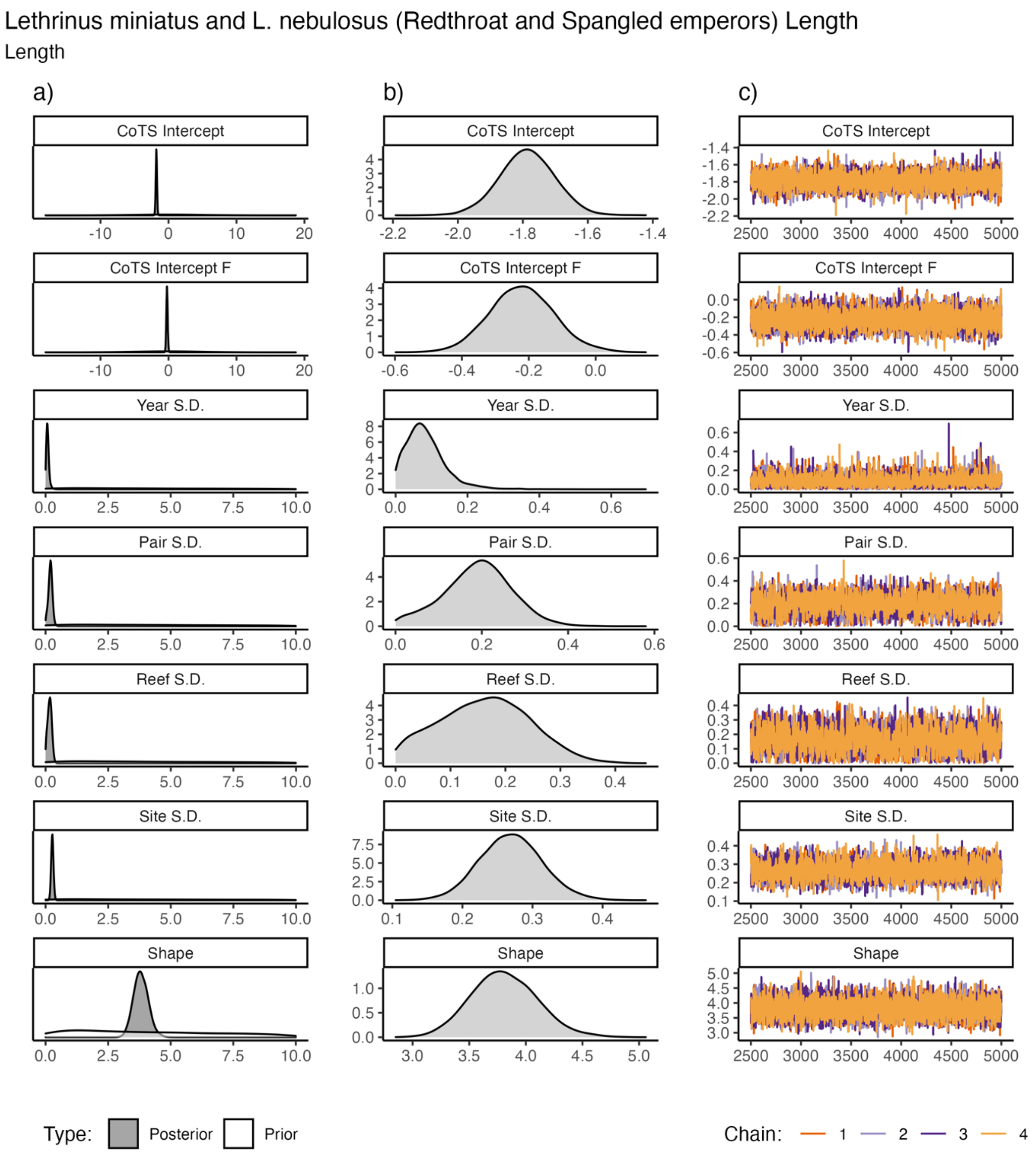

Supplementary Figure 57. Model validation checks.

a) Comparison between prior and posterior distribution of model parameters. b) Posterior distribution of model parameters. c) Chain mixing trace-plots.

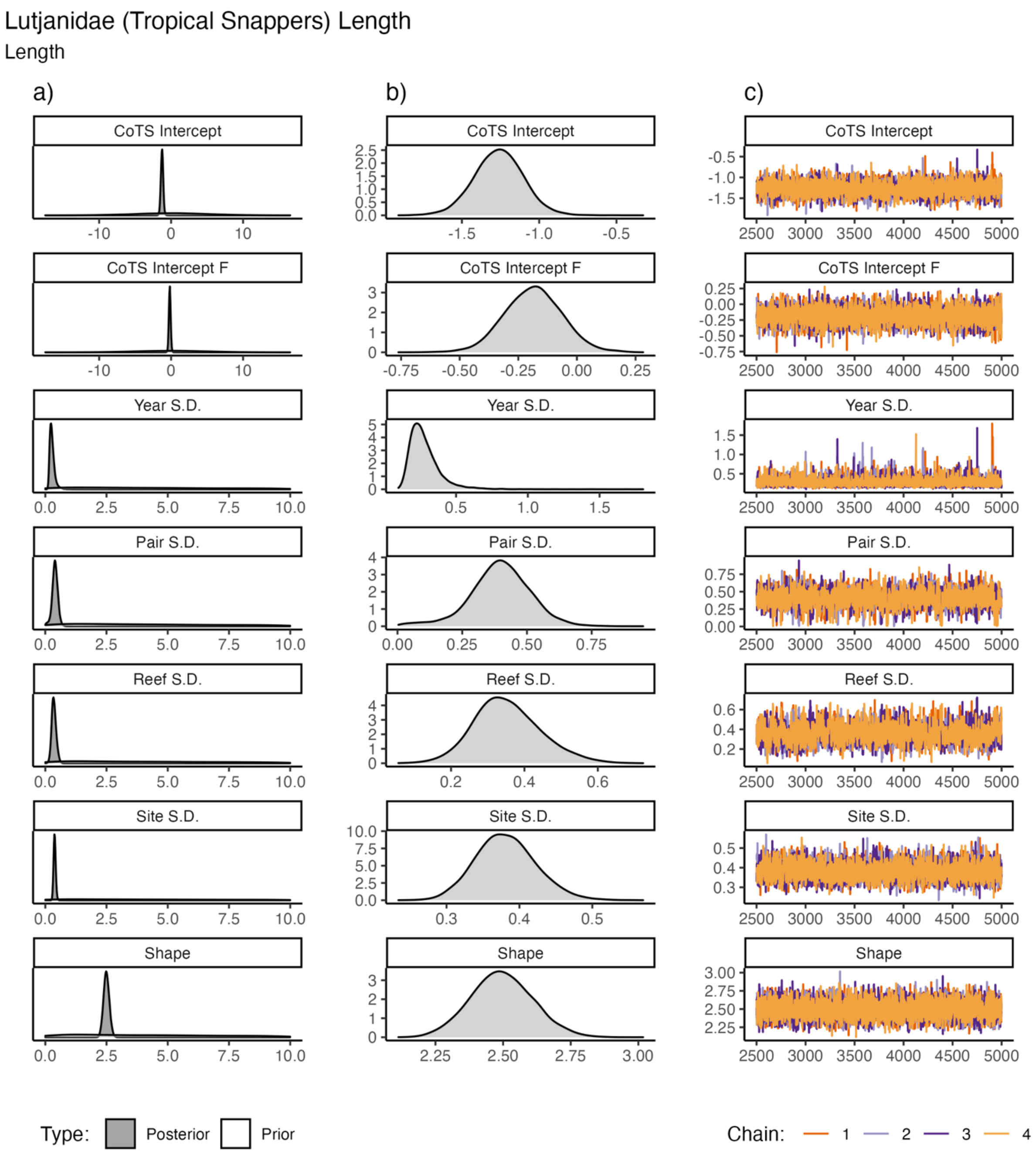

Supplementary Figure 58. Model validation checks.

a) Comparison between prior and posterior distribution of model parameters. b) Posterior distribution of model parameters. c) Chain mixing trace-plots.

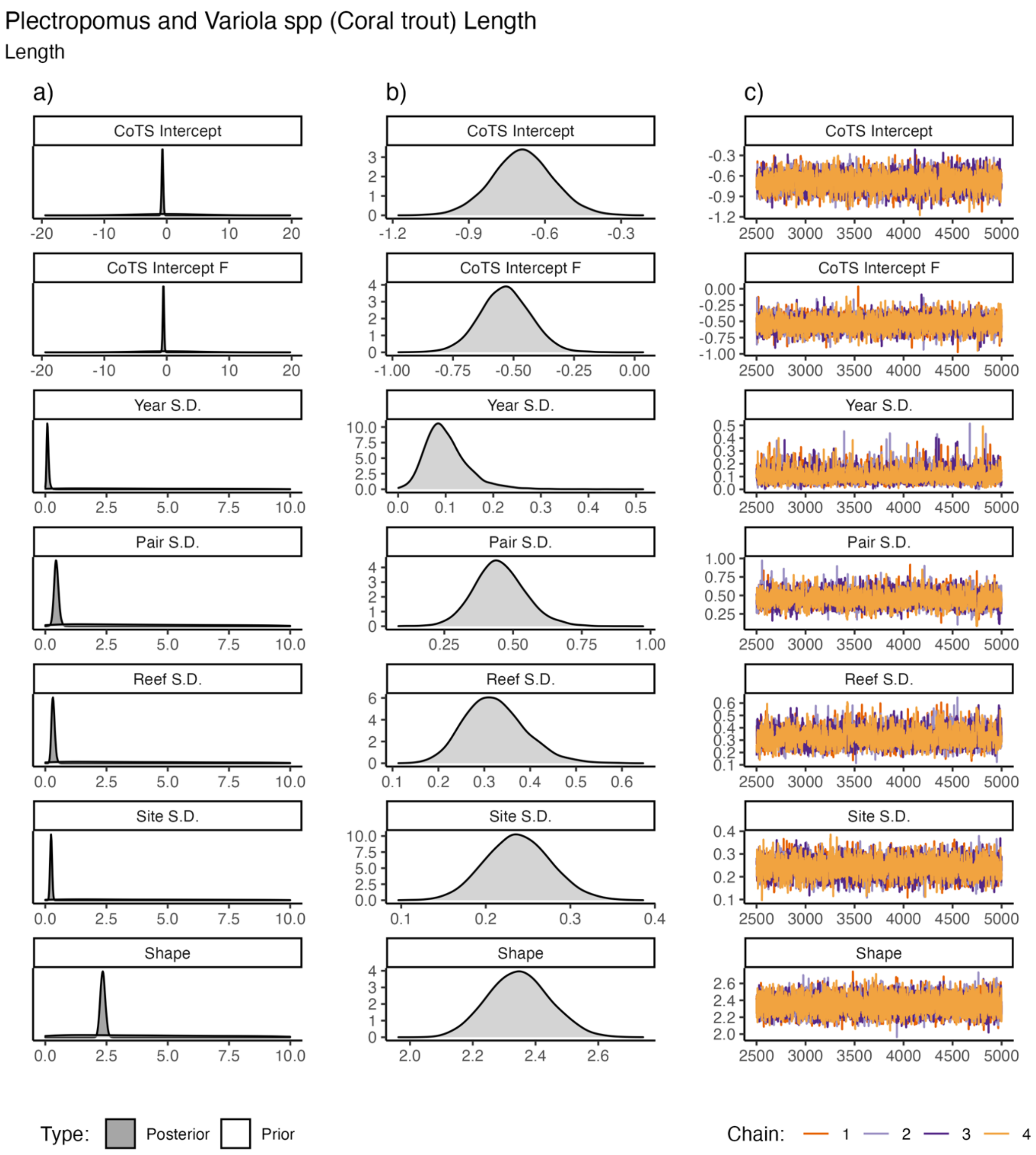

Supplementary Figure 59. Model validation checks.

a) Comparison between prior and posterior distribution of model parameters. b) Posterior distribution of model parameters. c) Chain mixing trace-plots.

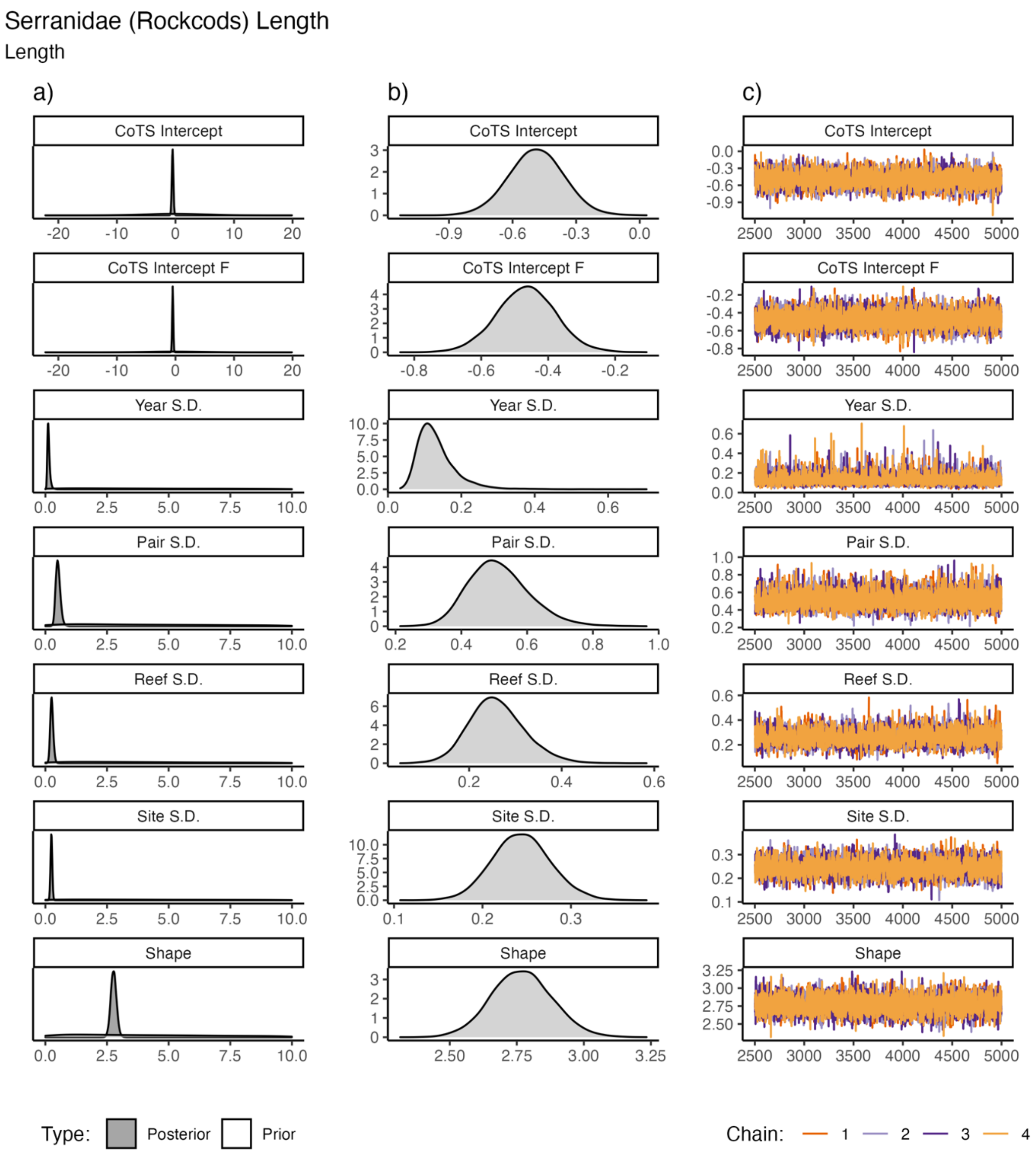

Supplementary Figure 60. Model validation checks.

a) Comparison between prior and posterior distribution of model parameters. b) Posterior distribution of model parameters. c) Chain mixing trace-plots.

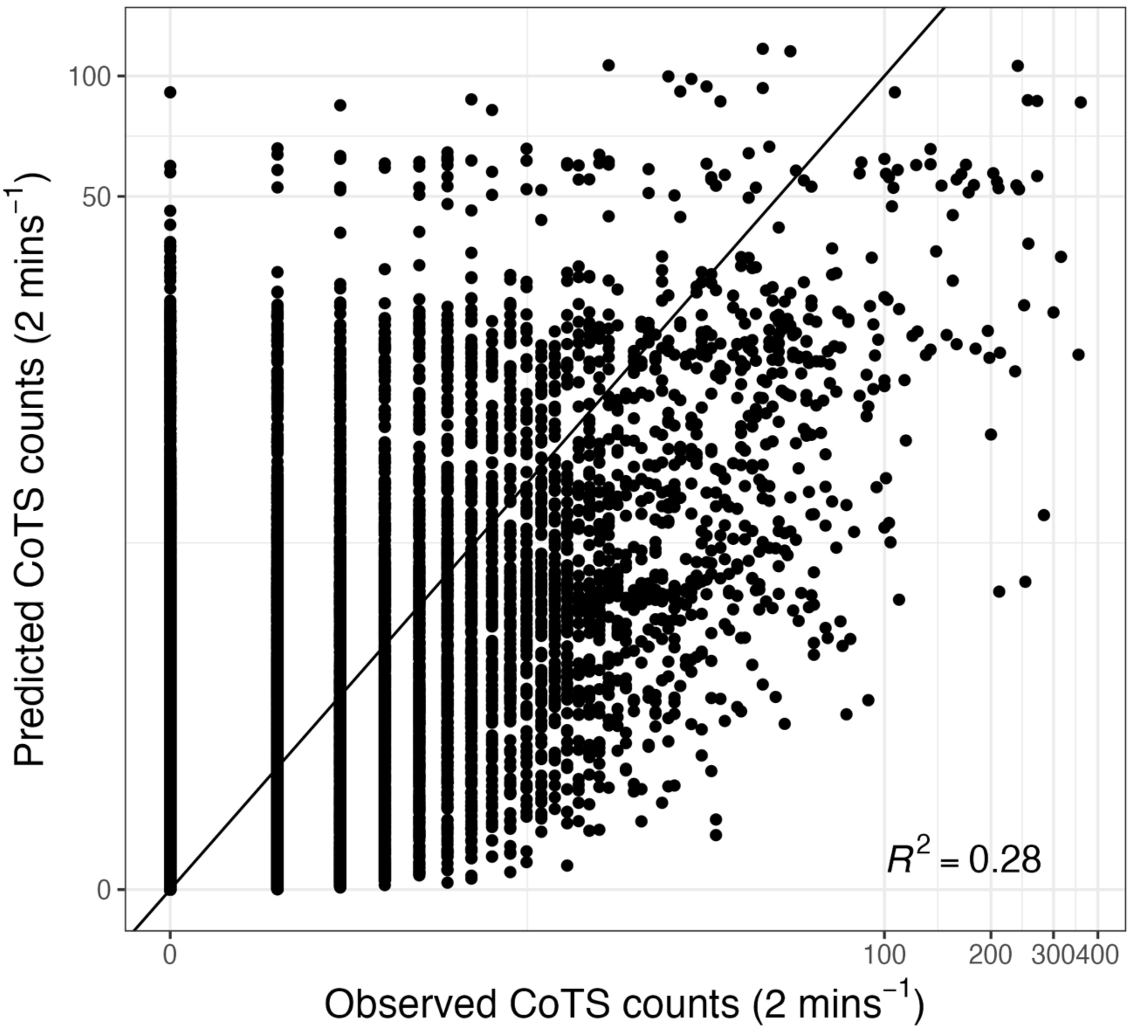

Supplementary Figure 61. Posterior predictive check of model predicting the role of reef zoning and coral cover on Crown-of-Thorns Starfish density.

Observed density Pacific Crown-of-Thorns Starfish (CoTS, *Acanthaster* cf. *solaris*) in the Great Barrier Reef Marine Park, Australia, on the x axis, and observation-level mean posterior prediction on the y axis. Line represents a 1-to-1 fit, both axes are displayed on a natural log scale. See model description in the Supplementary Method 4.

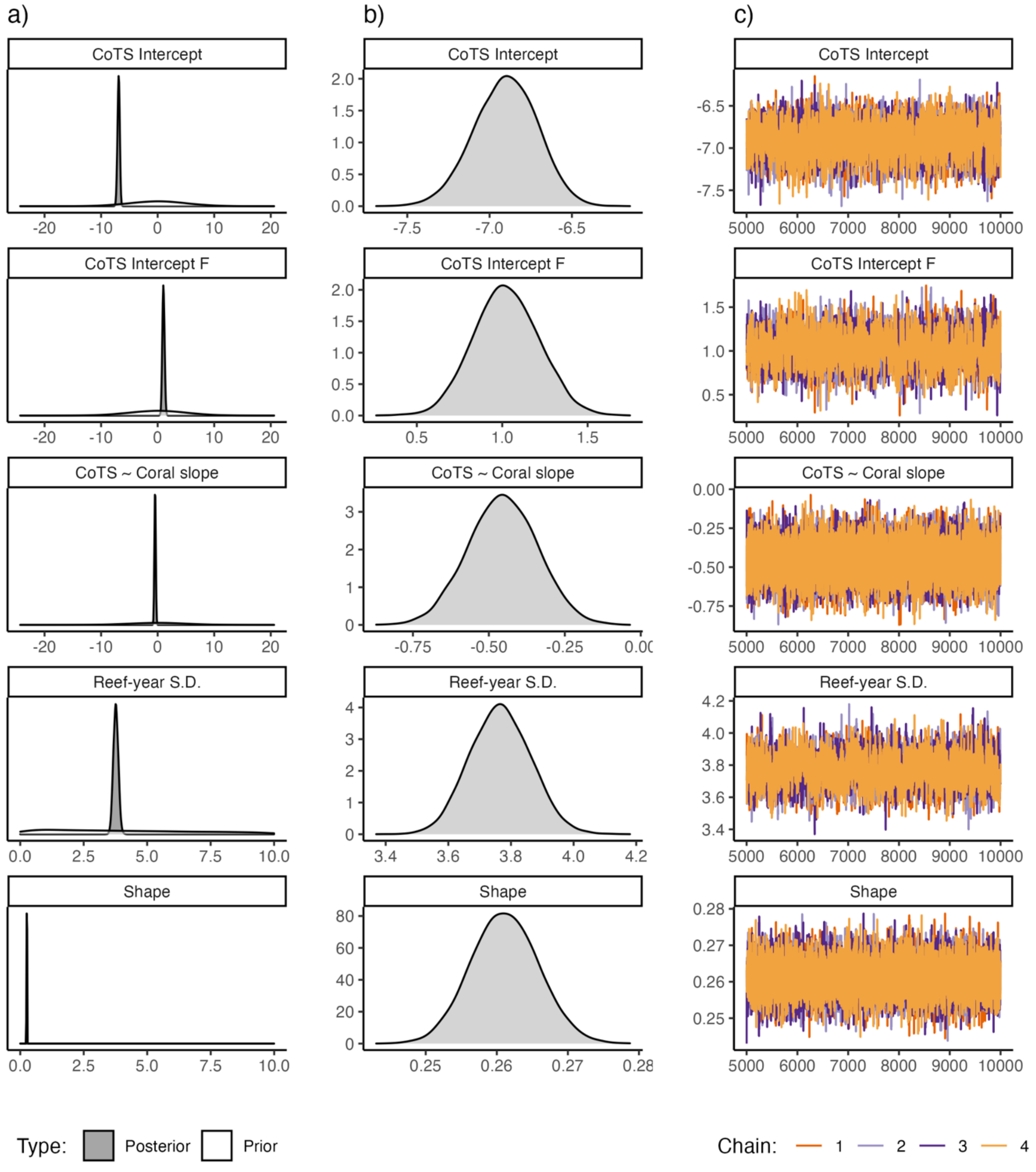

Supplementary Figure 62. Model validation checks.

a) Comparison between prior and posterior distribution of model parameters. b) Posterior distribution of model parameters. c) Chain mixing trace-plots.

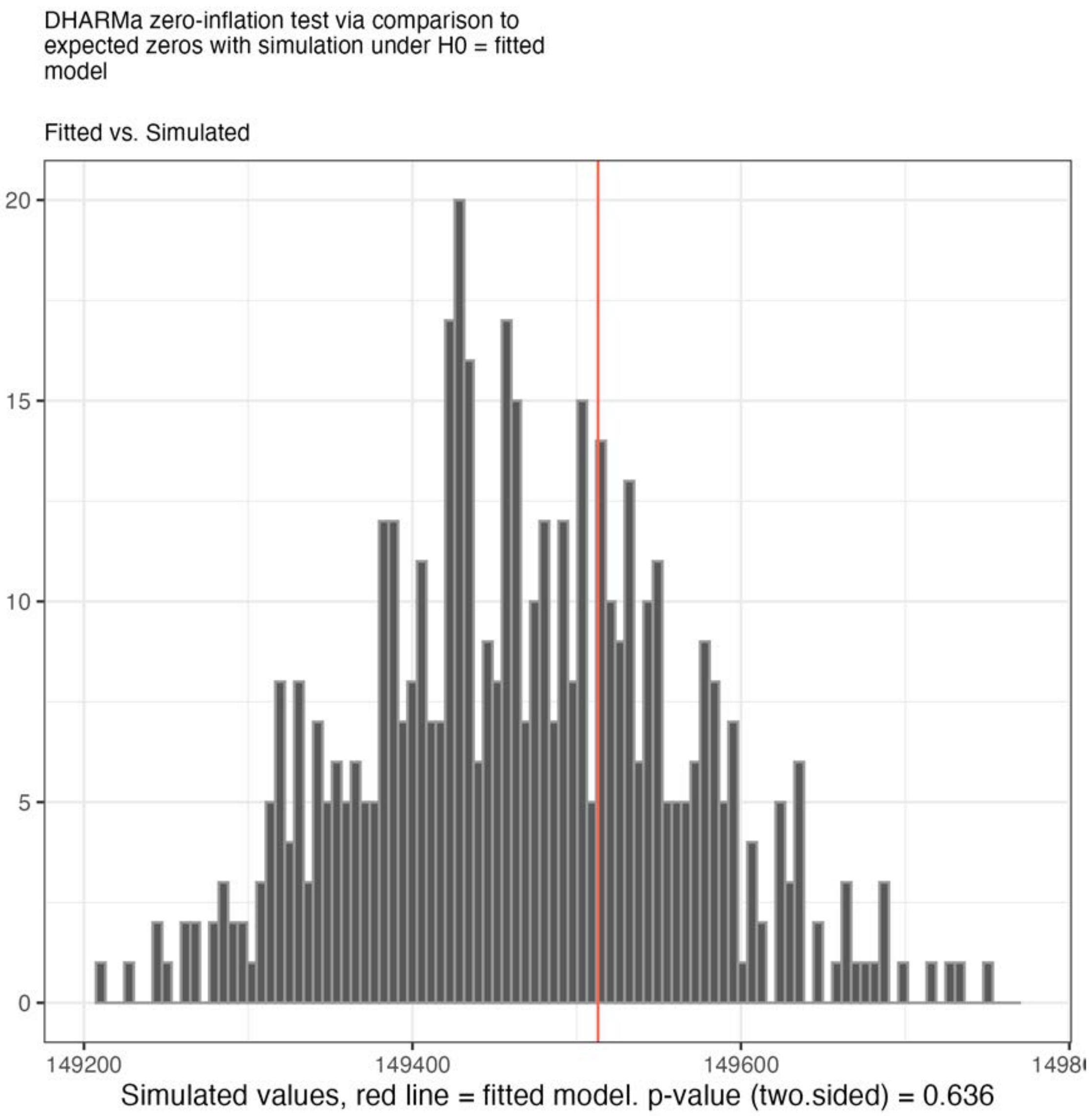

Supplementary Figure 63. Test of zero-inflation in Crown-of-Thorns Starfish data.

The test of zero-inflation, implemented in the R package DHARMa, compares the distribution of expected zeros in the data based on the model against the observed zeros. Results indicate that there are no differences (i.e. no evidence of zero-inflation). See model description in the Supplementary Method 4.

Supplementary Tables

Supplementary Table 1. Zoning (a) use and (b) area information for the Great Barrier Reef Marine Park, Australia, since 19813.

The different uses and activities that are allowed, prohibited or requiring a permit within each of the seven zones are presented (https://www.gbrmpa.gov.au/access-and-use/zoning/interpreting-zones). The proportional area that each of the seven zones covers within the Great Barrier Reef Marine Park is given for both before and since 2004.

(a)

Use	Great Barrier Reef Marine Park Zones						
	General Use (Light Blue)	Habitat Protection (Dark Blue)	Conservation Park (Yellow)	Buffer (Olive Green)	Scientific Research (Orange)	Marine National Park (Green)	Preservation (Pink)
Aquaculture	Permit	Permit	Permit	No	No	No	No
Bait netting	Yes	Yes	Yes	No	No	No	No
Boating, diving, photography	Yes	Yes	Yes	Yes	Yes	Yes	No
Crabbing (trapping)	Yes	Yes	Limited	No	No	No	No
Harvest fishing for aquarium fish, coral and beachworm	Permit	Permit	Permit	No	No	No	No
Harvest fishing for sea cucumber, trochus, tropical rock lobster	Permit	Permit	No	No	No	No	No
Limited collecting	Yes	Yes	Yes	No	No	No	No
Limited impact research	Yes	Yes	Yes	Yes	Yes	Yes	Permit
Limited spearfishing (snorkel only)	Yes	Yes	Yes	No	No	No	No
Line fishing	Yes	Yes	Limited	No	No	No	No
Netting (other than bait netting)	Yes	Yes	No	No	No	No	No
Research (other than limited impact)	Permit	Permit	Permit	Permit	Permit	Permit	Permit
Shipping (other than a designated shipping area)	Yes	Permit	Permit	Permit	Permit	Permit	No
Tourism program	Permit	Permit	Permit	Permit	Permit	Permit	No

Use	Great Barrier Reef Marine Park Zones						
	General Use (Light Blue)	Habitat Protection (Dark Blue)	Conservation Park (Yellow)	Buffer (Olive Green)	Scientific Research (Orange)	Marine National Park (Green)	Preservation (Pink)
Traditional use of marine resources	Permit (or TUMRA)	Permit (or TUMRA)	Permit (or TUMRA)	Permit (or TUMRA)	Permit (or TUMRA)	Permit (or TUMRA)	No
Trawling	Yes	No	No	No	No	No	No
Trolling	Yes	Yes	Yes	Yes	No	No	No

(b)

Area	Great Barrier Reef Marine Park Zones						
	General Use (Light Blue)	Habitat Protection (Dark Blue)	Conservation Park (Yellow)	Buffer (Olive Green)	Scientific Research (Orange)	Marine National Park (Green)	Preservation (Pink)
Percentage area <2004	77.90%	15.20%	0.60%	0.10%	0.01%	4.60%	0.10%
Percentage area ≥2004	33.80%	28.20%	1.50%	2.90%	0.05%	33.30%	0.20%

Supplementary Table 2. Coral reef fish species and families targeted by fisheries on the Great Barrier Reef Marine Park, Australia.

Coral reef fish species and families reported by commercial line, net and trawl, and recreational charter fisheries for the Great Barrier Reef Marine Park, Australia, from 1989 to 2018. Retained catch data was obtained for a total of 532 taxa that are either known or likely consumers of different life stages of Crown-of-Thorns Starfish (CoTS, *Acanthaster* spp.)[20], as well as for large piscivores that may influence CoTS population outbreaks through more complex trophic cascades[4, 39]. Only those species that were included in the statistical analyses are presented here, including family, scientific and common name; 'X' indicates species that have been reported to consume pelagic or benthic CoTS[20]. Retained catches of the common coral trout (*Plectropomus leopardus*) are included in total retained catch for the coral trout species complex (*Plectropomus* spp. and *Variola* spp.). CAAB code represents Codes for Australian Aquatic Biota (CAAB) number (https://www.cmar.csiro.au/data/caab/). Use of the data is by courtesy of the State of Queensland, Australia through the Department of Agriculture and Fisheries.

Scientific name	Common name	CoTS consumption	CAAB code
**Labridae**			
Achoerodus viridis	Groper - eastern blue		37384043
Austrolabrus maculatus	Wrasse - black spot		37384025
Bodianus bilunulatus	Saddleback - pigfish		37384054
Bodianus perditio	Pigfish - gold spot		37384007
Bodianus spp.	Pigfish - unspecified		37384904
Bodianus unimaculatus	Eastern Pigfish		37384061
Cheilinus fasciatus	Redbreast Maori Wrasse	X	37384066
Cheilinus undulatus	Wrasse - humphead maori	X	37384038
Choerodon cephalotes	Tusk fish - purple		37384004
Choerodon cyanodus	Tusk fish - blue		37384072
Choerodon fasciatus	Tusk fish - harlequin		37384073

Scientific name	Common name	CoTS consumption	CAAB code
Choerodon japonicus	Tusk fish - purple line		37384076
Choerodon schoenleinii	Tusk fish - black spot		37384010
Choerodon spp.	Tusk fish - unspecified		37384902
Choerodon venustus	Tusk fish - venus		37384042
Epibulus insidiator	Wrasse - sling-jaw		37384104
Halichoeres trimaculatus	Wrasse - three spot		37384122
Labridae - undifferentiated	Wrasse - unspecified		37384000
Thalassoma lunare	Wrasse - moon	X	37384167
Unnamed	[a wrasse]		37384903
Xiphocheilus typus	Wrasse - blue banded		37384014
**Lethrinidae**			
Gymnocranius audleyi	Seabream - Collar		37351018
Gymnocranius elongatus	Seaperch - swallowtail		37351010
Gymnocranius euanus	Bream - japanese large-eye		37351022
Gymnocranius grandoculis	Bream - maori	X	37351005
Lethrinidae - undifferentiated	Emperor - Unspecified		37351000
Lethrinus atkinsoni	Emperor - yellow tailed	X	37351013
Lethrinus erythracanthus	Emperor - yellow spotted		37351025
Lethrinus erythropterus	Longfin Emperor		37351029
Lethrinus genivittatus	Lancer		37351002
Lethrinus laticaudis	Emperor - grass	X	37351006
Lethrinus lentjan	Emperor - pink-eared	X	37351007
Lethrinus microdon	Smalltooth Emperor		37351011
Lethrinus miniatus	Emperor - red throat	X	37351009
Lethrinus nebulosus	Emperor - spangled	X	37351008
Lethrinus obsoletus	Emperor - orange striped		37351019
Lethrinus olivaceus	Emperor - long nose		37351004
Lethrinus ornatus	Ornate Emperor	X	37351015
Lethrinus rubrioperculatus	Emperor - red ear		37351012
Lethrinus semicinctus	Emperor - reticulated		37351016
Lethrinus variegatus	Emperor - variegated		37351014
Lethrinus xanthochilus	Emperor - yellow lipped		37351020
Monotaxis grandoculis	Sea bream - big eye	X	37351026
Wattsia mossambica	Bream - mozambique		37351027
**Lutjanidae**			
Aphareus furca	Jobfish - small-toothed		37346036
Aphareus rutilans	Rusty jobfish		37346001
Aprion virescens	Jobfish - green		37346027
Etelis carbunculus	Snapper - ruby		37346014
Etelis coruscans	Snapper - flame tail		37346038
Etelis radiosus	Snapper - pale		37346058
Etelis spp.	Tropical snapper		37346914
Lipocheilus carnolabrum	Tang's snapper		37346031

Scientific name	Common name	CoTS consumption	CAAB code
Lutjanus argentimaculatus	Mangrove jack		37346015
Lutjanus bitaeniatus	Snapper - indonesian		37346025
Lutjanus bohar	Bass - red	X	37346029
Lutjanus carponotatus	Stripey - spanish flag		37346011
Lutjanus erythropterus	Nannygai - small mouth		37346005
Lutjanus fulviflamma	Snapper - black spot		37346034
Lutjanus gibbus	Paddle tail	X	37346028
Lutjanus johnii	Golden Snapper		37346030
Lutjanus lemniscatus	Perch - dark tailed sea		37346010
Lutjanus malabaricus	Nannygai - large mouth		37346007
Lutjanus malabaricus and *L. erythropterus*	Nannygai - unspecified		37346911
Lutjanus monostigma	Snapper - onespot		37346045
Lutjanus quinquelineatus	Fiveline Snapper		37346006
Lutjanus rivulatus	Perch - maori sea		37346016
Lutjanus russelli	Perch - moses	X	37346065
Lutjanus russellii	Tropical snapper		37346915
Lutjanus sebae	Emperor - red		37346004
Lutjanus vitta and *L. adetii*	Hussar - unspecified		37346910
Macolor macularis	Midnight Snapper		37346047
Macolor niger	Snapper - black and white		37346048
Paracaesio kusaharii	Snapper - saddleback		37346060
Paracaesio stonei	Cocoa snapper		37346053
Paracaesio xanthura	Fusilier - southern		37346049
Pristipomoides argyrogrammicus	Ornate snapper		37346054
Pristipomoides filamentosus	Jobfish - rosy		37346032
Pristipomoides flavipinnis	Goldeneye snapper		37346055
Pristipomoides multidens	Jobfish - gold banded		37346002
Pristipomoides multidens and *P. typus*	Jobfish - unspecified		37346901
Pristipomoides sieboldii	Lavender snapper		37346064
Pristipomoides spp.	[a tropical snapper]		37346916
Pristipomoides typus	Sharptooth snapper		37346019
Pristipomoides zonatus	Oblique-banded snapper		37346056
Symphorus nematophorus	Chinaman		37346017
**Serranidae**			
Acanthistius ocellatus	Mother in law		37311090
Aethaloperca rogaa	Cod - red flushed		37311134
Anyperodon leucogrammicus	Cod - white lined		37311085
Caprodon krasyukovae	Krasyukova's Perch		37311189
Caprodon schlegelii	Perch - sunrise		37311096
Cephalopholis argus	Peacock cod		37311082
Cephalopholis boenak	Cod - brown banded		37311008
Cephalopholis cyanostigma	Cod - blue spot rock		37311136
Cephalopholis formosa	Cod - tomato		37311137

Scientific name	Common name	CoTS consumption	CAAB code
Cephalopholis leopardus	Cod - leopard rock		37311138
Cephalopholis miniata	Cod - coral		37311083
Cephalopholis sexmaculata	Cod - freckled		37311140
Cephalopholis sonnerati	Cod - tomato		37311045
Cephalopholis spiloparaea	Cod - strawberry rock		37311141
Cromileptes altivelis	Cod - barramundi		37311044
Epinephelus and *Cephalopholis*	Cod - unspecified		37311909
Epinephelus ergastularius and *E. septemfasciatus*	Cod - bar		37311910
Epinephelus coioides	Cod - estuary		37311007
Epinephelus areolatus	Cod - yellow spotted rock		37311009
Epinephelus coeruleopunctatus	Whitespotted Grouper		37311070
Epinephelus cyanopodus	Cod - blue maori	X	37311145
Epinephelus daemelii	Black Rockcod		37311077
Epinephelus ergastularius	Banded Rockcod		37311147
Epinephelus fasciatus	Cod - black-tipped rock		37311014
Epinephelus fuscoguttatus	Cod - flowery		37311021
Epinephelus howlandi	Blacksaddle Rockcod		37311148
Epinephelus lanceolatus	Groper - queensland	X	37311061
Epinephelus maculatus	Cod - black-finned		37311011
Epinephelus magniscuttis	Speckled grouper		37311173
Epinephelus malabaricus	Cod - morgan's		37311150
Epinephelus merra	Cod - birdwire		37311063
Epinephelus morrhua	Grouper - comet		37311151
Epinephelus octofasciatus	Grouper - eight bar		37311152
Epinephelus ongus	Cod - speckled fin		37311069
Epinephelus polyphekadion	Camouflage rockcod		37311047
Epinephelus quoyanus	Cod - long finned		37311040
Epinephelus radiatus	Radiant rockcod		37311042
Epinephelus tauvina	Cod - greasy		37311057
Epinephelus timorensis	Yellowspotted Grouper		37311073
Epinephelus tukula	Cod - potato		37311068
Epinephelus undulatostriatus	Cod - maori		37311086
Plectropomus spp. and *Variola* spp.	Coral trout		37311905
Plectropomus areolatus	Trout - passionfruit		37311081
Plectropomus laevis	Trout - blue spot		37311079
Plectropomus maculatus	Trout - island		37311012
Serranidae subfam. *Anthiinae*	Anthias		37311907
Serranidae subfam. *Epinephelinae*	Cod - reef unspecified		37311908
Unnamed	[a rockcod]		37311904
Variola louti	Cod - coronation trout		37311166

Supplementary Table 3. Model estimates and hypothesis testing for CoTS density as a function of fish biomass removal on reefs that are exposed to fishing.

Parameter estimates were obtained by employing hurdle-gamma models via a Bayesian approach. Models were fitted to biomass removal data for six fish groups, namely (1) Labridae (wrasses), (2) Lethrinidae (emperors), (3) *Lethrinus miniatus* and *L. nebulosus* (redthroat and spangled emperors), (4) Lutjanidae (tropical snappers), (5) Serranidae (rockcods) and (6) *Plectropomus* spp. and *Variola* spp. (coral trout), at six time lags (in years) each. Mean parameter estimates, as well as their 95% Bayesian credible intervals, are presented on the linear predictor scale (with the exception of Prob. 0; please see Supplementary Method 2 for model descriptions). The "Test" column comprises the actual parameter hypotheses tests following a priori expectations, i.e. whether a parameter is lower or greater than 0. The "Test probability" column comprises the exceedance probability i.e. the proportion of a parameter's posterior distribution which supports the test. CoTS density (natural log scale) = β_0; CC (coral cover) slope = β_1; Fish density slope = β_2; Prob. 0 (probability of CoTS density being zero) = $logit^{-1}(\theta_0)$; Hurdle CC slope = θ_1. Twenty of the 36 models provided more than 80% probability, and ten of the 36 models more than 95% probability, that CoTS densities on fished reefs increase with increasing retained catches of coral reef fish.

(1) Labridae (wrasses)

Time lag	Parameter	Estimate	Q2.5	Q97.5	Test name	Test probability
1	CoTS density	-0.61	-1.99	0.84	-	-
	CC slope	-4.37	-7.34	-1.4	< 0	99.81
	Fish density slope	0.01	-0.3	0.31	> 0	53.41
	Prob. 0	0.82	0.71	0.91	-	-
	Hurdle CC slope	4.11	1.98	6.34	> 0	100
2	CoTS density	-0.29	-1.57	1.01	-	-
	CC slope	-3.62	-6.21	-1.06	< 0	99.59
	Fish density slope	-0.23	-0.5	0.03	> 0	4.22
	Prob. 0	0.76	0.64	0.85	-	-
	Hurdle CC slope	2.43	0.48	4.41	> 0	99.48
3	CoTS density	-1.66	-2.99	-0.29	-	-
	CC slope	-3.22	-5.83	-0.55	< 0	99.05
	Fish density slope	0.09	-0.21	0.38	> 0	71.94
	Prob. 0	0.76	0.65	0.86	-	-
	Hurdle CC slope	2.86	0.91	4.9	> 0	99.88
4	CoTS density	-0.7	-2.1	0.77	-	-
	CC slope	-3.66	-6.43	-0.81	< 0	99.21
	Fish density slope	-0.05	-0.35	0.25	> 0	37.78
	Prob. 0	0.74	0.62	0.85	-	-
	Hurdle CC slope	1.89	-0.15	4.04	> 0	96.39
5	CoTS density	-1.1	-2.88	0.74	-	-
	CC slope	-4.08	-7.54	-0.69	< 0	99.06
	Fish density slope	0	-0.42	0.42	> 0	50.27
	Prob. 0	0.77	0.63	0.88	-	-
	Hurdle CC slope	2.57	0.2	4.94	> 0	98.41
6	CoTS density	-0.72	-2.18	0.77	-	-
	CC slope	-3.03	-5.86	-0.13	< 0	97.9
	Fish density slope	-0.2	-0.56	0.15	> 0	12.36
	Prob. 0	0.73	0.59	0.84	-	-
	Hurdle CC slope	1.26	-0.86	3.39	> 0	88.25

(2) Lethrinidae (emperors)

Time lag	Parameter	Estimate	Q2.5	Q97.5	Test name	Test probability
1	CoTS density	-2.45	-3.72	-1.16	-	-
	CC slope	-5.47	-7.63	-3.26	< 0	100
	Fish density slope	0.33	0.14	0.53	> 0	99.96
	Prob. 0	0.73	0.63	0.82	-	-

Time lag	Parameter	Estimate	Q2.5	Q97.5	Test name	Test probability
	Hurdle CC slope	1.33	-0.17	2.83	> 0	95.86
2	CoTS density	-2.2	-3.27	-1.11	-	-
	CC slope	-4.42	-6.53	-2.27	< 0	99.99
	Fish density slope	0.25	0.08	0.41	> 0	99.85
	Prob. 0	0.7	0.6	0.79	-	-
	Hurdle CC slope	0.95	-0.45	2.41	> 0	90.62
3	CoTS density	-0.64	-1.73	0.45	-	-
	CC slope	-4.31	-6.27	-2.26	< 0	100
	Fish density slope	-0.03	-0.19	0.13	> 0	37.63
	Prob. 0	0.72	0.63	0.8	-	-
	Hurdle CC slope	1.44	0.11	2.81	> 0	98.21
4	CoTS density	-1.7	-2.76	-0.63	-	-
	CC slope	-4.41	-6.41	-2.35	< 0	99.99
	Fish density slope	0.15	-0.01	0.31	> 0	96.59
	Prob. 0	0.68	0.58	0.77	-	-
	Hurdle CC slope	0.56	-0.9	2.02	> 0	77.4
5	CoTS density	-1.55	-2.75	-0.37	-	-
	CC slope	-5.38	-7.83	-2.78	< 0	100
	Fish density slope	0.17	-0.02	0.36	> 0	96.51
	Prob. 0	0.73	0.61	0.82	-	-
	Hurdle CC slope	1.34	-0.29	3.01	> 0	94.6
6	CoTS density	-1.2	-2.16	-0.18	-	-
	CC slope	-4.48	-6.56	-2.34	< 0	100
	Fish density slope	0.06	-0.1	0.21	> 0	75.88
	Prob. 0	0.73	0.63	0.81	-	-
	Hurdle CC slope	1.14	-0.31	2.6	> 0	93.94

(3) *Lethrinus miniatus* and *L. nebulosus* (Redthroat and Spangled emperors)

Time lag	Parameter	Estimate	Q2.5	Q97.5	Test name	Test probability
1	CoTS density	-1.45	-2.69	-0.21	-	-
	CC slope	-5.69	-7.96	-3.35	< 0	100
	Fish density slope	0.19	0.01	0.38	> 0	97.85
	Prob. 0	0.75	0.65	0.83	-	-
	Hurdle CC slope	1.55	-0.03	3.19	> 0	97.14
2	CoTS density	-1.88	-2.99	-0.73	-	-
	CC slope	-4.62	-6.82	-2.35	< 0	99.99
	Fish density slope	0.21	0.03	0.39	> 0	99.08
	Prob. 0	0.73	0.64	0.82	-	-
	Hurdle CC slope	1.35	-0.1	2.85	> 0	96.65

Time lag	Parameter	Estimate	Q2.5	Q97.5	Test name	Test probability
3	CoTS density	-1.14	-2.26	-0.01	-	-
	CC slope	-4.54	-6.62	-2.46	< 0	100
	Fish density slope	0.06	-0.11	0.23	> 0	76.89
	Prob. 0	0.74	0.65	0.82	-	-
	Hurdle CC slope	1.59	0.15	3.08	> 0	98.57
4	CoTS density	-1.69	-2.8	-0.59	-	-
	CC slope	-4.21	-6.25	-2.12	< 0	100
	Fish density slope	0.16	-0.01	0.33	> 0	96.43
	Prob. 0	0.7	0.6	0.8	-	-
	Hurdle CC slope	0.81	-0.72	2.34	> 0	85.65
5	CoTS density	-0.55	-1.84	0.81	-	-
	CC slope	-4.97	-7.58	-2.34	< 0	99.99
	Fish density slope	0	-0.22	0.21	> 0	49.09
	Prob. 0	0.74	0.63	0.84	-	-
	Hurdle CC slope	1.52	-0.23	3.31	> 0	95.62
6	CoTS density	-1.35	-2.37	-0.32	-	-
	CC slope	-4.41	-6.58	-2.17	< 0	99.99
	Fish density slope	0.09	-0.08	0.26	> 0	85.54
	Prob. 0	0.73	0.63	0.82	-	-
	Hurdle CC slope	1.14	-0.35	2.68	> 0	93.21

(4) Lutjanidae (tropical snappers)

Time lag	Parameter	Estimate	Q2.5	Q97.5	Test name	Test probability
1	CoTS density	-1.71	-3.19	-0.27	-	-
	CC slope	-5.15	-7.62	-2.62	< 0	99.99
	Fish density slope	0.21	-0.02	0.45	> 0	96.53
	Prob. 0	0.72	0.6	0.82	-	-
	Hurdle CC slope	1.22	-0.49	2.95	> 0	91.46
2	CoTS density	-1.45	-2.62	-0.26	-	-
	CC slope	-3.82	-6.06	-1.52	< 0	99.89
	Fish density slope	0.11	-0.08	0.29	> 0	87.09
	Prob. 0	0.67	0.56	0.77	-	-
	Hurdle CC slope	0.7	-0.8	2.24	> 0	81.39
3	CoTS density	-1.5	-2.42	-0.54	-	-
	CC slope	-3.43	-5.58	-1.27	< 0	99.87
	Fish density slope	0.09	-0.06	0.23	> 0	86.8
	Prob. 0	0.7	0.6	0.79	-	-
	Hurdle CC slope	1.38	-0.13	2.93	> 0	96.31
4	CoTS density	-1.24	-2.24	-0.23	-	-

Time lag	Parameter	Estimate	Q2.5	Q97.5	Test name	Test probability
	CC slope	-4.35	-6.69	-1.97	< 0	99.99
	Fish density slope	0.07	-0.09	0.23	> 0	81.06
	Prob. 0	0.65	0.53	0.77	-	-
	Hurdle CC slope	0.3	-1.36	1.94	> 0	64.11
5	CoTS density	-0.41	-1.67	0.89	-	-
	CC slope	-4.14	-6.89	-1.34	< 0	99.7
	Fish density slope	-0.07	-0.28	0.14	> 0	27.19
	Prob. 0	0.69	0.55	0.8	-	-
	Hurdle CC slope	0.91	-0.96	2.76	> 0	83.13
6	CoTS density	-0.24	-1.27	0.75	-	-
	CC slope	-3.94	-6.15	-1.66	< 0	99.93
	Fish density slope	-0.19	-0.37	-0.01	> 0	1.9
	Prob. 0	0.69	0.57	0.8	-	-
	Hurdle CC slope	0.53	-1.07	2.14	> 0	74.05

(5) Serranidae (rockcods)

Time lag	Parameter	Estimate	Q2.5	Q97.5	Test name	Test probability
1	CoTS density	-1.59	-3.19	0.03	-	-
	CC slope	-5.25	-7.43	-2.98	< 0	100
	Fish density slope	0.14	-0.07	0.34	> 0	90.89
	Prob. 0	0.76	0.66	0.83	-	-
	Hurdle CC slope	1.61	0.1	3.15	> 0	98.21
2	CoTS density	-1.79	-3.08	-0.46	-	-
	CC slope	-4.42	-6.5	-2.32	< 0	99.99
	Fish density slope	0.14	-0.04	0.31	> 0	93.9
	Prob. 0	0.7	0.6	0.79	-	-
	Hurdle CC slope	0.95	-0.44	2.36	> 0	90.93
3	CoTS density	-0.56	-2.05	0.95	-	-
	CC slope	-4.4	-6.39	-2.36	< 0	100
	Fish density slope	-0.04	-0.23	0.15	> 0	34.45
	Prob. 0	0.74	0.65	0.81	-	-
	Hurdle CC slope	1.63	0.29	3.01	> 0	99.1
4	CoTS density	-2.45	-3.7	-1.18	-	-
	CC slope	-4.37	-6.37	-2.4	< 0	100
	Fish density slope	0.22	0.06	0.39	> 0	99.57
	Prob. 0	0.7	0.6	0.79	-	-
	Hurdle CC slope	0.79	-0.59	2.2	> 0	86.77
5	CoTS density	-1.07	-2.74	0.67	-	-
	CC slope	-5	-7.51	-2.43	< 0	99.98
	Fish density slope	0.06	-0.18	0.29	> 0	69.23

Time lag	Parameter	Estimate	Q2.5	Q97.5	Test name	Test probability
	Prob. 0	0.74	0.63	0.83	-	-
	Hurdle CC slope	1.4	-0.26	3.12	> 0	94.99
6	CoTS density	-1.12	-2.38	0.18	-	-
	CC slope	-4.23	-6.26	-2.16	< 0	100
	Fish density slope	0.02	-0.15	0.19	> 0	60.4
	Prob. 0	0.73	0.63	0.81	-	-
	Hurdle CC slope	1.06	-0.32	2.45	> 0	93.34

(6) *Plectropomus* spp. and *Variola* spp. (coral trout)

Time lag	Parameter	Estimate	Q2.5	Q97.5	Test name	Test probability
1	CoTS density	-1.36	-2.94	0.24	-	-
	CC slope	-5.15	-7.47	-2.9	< 0	100
	Fish density slope	0.1	-0.1	0.3	> 0	83.7
	Prob. 0	0.75	0.66	0.83	-	-
	Hurdle CC slope	1.49	-0.03	3.06	> 0	97.22
2	CoTS density	-1.89	-3.15	-0.55	-	-
	CC slope	-4.1	-6.19	-1.93	< 0	99.99
	Fish density slope	0.14	-0.04	0.31	> 0	94.52
	Prob. 0	0.7	0.6	0.79	-	-
	Hurdle CC slope	0.94	-0.45	2.39	> 0	90.82
3	CoTS density	-0.37	-1.86	1.18	-	-
	CC slope	-4.49	-6.54	-2.4	< 0	100
	Fish density slope	-0.06	-0.26	0.13	> 0	26.17
	Prob. 0	0.74	0.65	0.81	-	-
	Hurdle CC slope	1.65	0.26	3.06	> 0	99.1
4	CoTS density	-3.09	-4.46	-1.7	-	-
	CC slope	-3.66	-5.68	-1.68	< 0	100
	Fish density slope	0.3	0.12	0.47	> 0	99.93
	Prob. 0	0.7	0.6	0.79	-	-
	Hurdle CC slope	0.76	-0.67	2.19	> 0	84.7
5	CoTS density	-1.84	-3.66	-0.01	-	-
	CC slope	-4.36	-7.03	-1.68	< 0	99.89
	Fish density slope	0.15	-0.09	0.39	> 0	88.99
	Prob. 0	0.74	0.62	0.83	-	-
	Hurdle CC slope	1.34	-0.37	3	> 0	93.65
6	CoTS density	-1.56	-2.91	-0.17	-	-
	CC slope	-3.9	-6	-1.8	< 0	99.97
	Fish density slope	0.08	-0.1	0.25	> 0	80.94
	Prob. 0	0.72	0.63	0.81	-	-
	Hurdle CC slope	0.99	-0.43	2.41	> 0	91.58

Supplementary Table 4. Pairwise Pearson correlation values for fish biomass removal between the six fish groups for each of the six time lags.

Pearson correlation values were calculated for fish biomass removed between paired fish groups, for each of the six time lags (in years) separately.

**Time lag**	***Plectropomus* and *Variola* spp. (Coral trout)**	**Serranidae (Rockcods)**	***Lethrinus miniatus* and *L. nebulosus* (Redthroat and Spangled emperors)**	**Lethrinidae (Emperors)**	**Lutjanidae (Tropical snappers)**	**Labridae (Wrasses)**
**1 yr**						
Plectropomus and *Variola* spp. (Coral trout)	1.000	0.981	0.622	0.621	0.062	0.254
Serranidae (Rockcods)		1.000	0.650	0.637	0.089	0.310
Lethrinus miniatus and *L. nebulosus* (Redthroat and Spangled emperors)			1.000	0.970	0.120	0.420
Lethrinidae (Emperors)				1.000	0.178	0.415
Lutjanidae (Tropical snappers)					1.000	0.360
Labridae (Wrasses)						1.000
**2 yrs**						
Plectropomus and *Variola* spp. (Coral trout)	1.000	0.993	0.644	0.652	0.146	0.262
Serranidae (Rockcods)		1.000	0.667	0.677	0.173	0.326
Lethrinus miniatus and *L. nebulosus* (Redthroat and Spangled emperors)			1.000	0.970	0.154	0.435
Lethrinidae (Emperors)				1.000	0.214	0.458
Lutjanidae (Tropical snappers)					1.000	0.421

Time lag	*Plectropomus* and *Variola* spp. (Coral trout)	Serranidae (Rockcods)	*Lethrinus miniatus* and *L. nebulosus* (Redthroat and Spangled emperors)	Lethrinidae (Emperors)	Lutjanidae (Tropical snappers)	Labridae (Wrasses)
Labridae (Wrasses)						1.000
**3 yrs**						
Plectropomus and *Variola* spp. (Coral trout)	1.000	0.983	0.193	0.564	0.615	0.070
Serranidae (Rockcods)		1.000	0.268	0.594	0.639	0.103
Lethrinus miniatus and *L. nebulosus* (Redthroat and Spangled emperors)			1.000	0.313	0.378	0.374
Lethrinidae (Emperors)				1.000	0.948	0.100
Lutjanidae (Tropical snappers)					1.000	0.141
Labridae (Wrasses)						1.000
**4 yrs**						
Plectropomus and *Variola* spp. (Coral trout)	1.000	0.982	0.650	0.678	0.152	0.356
Serranidae (Rockcods)		1.000	0.672	0.693	0.189	0.411
Lethrinus miniatus and *L. nebulosus* (Redthroat and Spangled emperors)			1.000	0.969	0.151	0.500
Lethrinidae (Emperors)				1.000	0.215	0.545
Lutjanidae (Tropical snappers)					1.000	0.403
Labridae (Wrasses)						1.000
**5 yrs**						
Plectropomus and *Variola* spp. (Coral trout)	1.000	0.989	0.645	0.642	0.079	0.195
Serranidae (Rockcods)		1.000	0.674	0.671	0.124	0.274

**Time lag**	***Plectropomus* and *Variola* spp. (Coral trout)**	**Serranidae (Rockcods)**	***Lethrinus miniatus* and *L. nebulosus* (Redthroat and Spangled emperors)**	**Lethrinidae (Emperors)**	**Lutjanidae (Tropical snappers)**	**Labridae (Wrasses)**
Lethrinus miniatus and *L. nebulosus* (Redthroat and Spangled emperors)			1.000	0.961	0.124	0.421
Lethrinidae (Emperors)				1.000	0.187	0.472
Lutjanidae (Tropical snappers)					1.000	0.544
Labridae (Wrasses)						1.000
**6 yrs**						
Plectropomus and *Variola* spp. (Coral trout)	1.000	0.984	0.155	0.599	0.653	0.308
Serranidae (Rockcods)		1.000	0.184	0.618	0.672	0.358
Lethrinus miniatus and *L. nebulosus* (Redthroat and Spangled emperors)			1.000	0.107	0.184	0.485
Lethrinidae (Emperors)				1.000	0.941	0.399
Lutjanidae (Tropical snappers)					1.000	0.441
Labridae (Wrasses)						1.000

Supplementary Table 5. Coral reef fish species and families monitored on the Great Barrier Reef Marine Park, Australia.

Coral reef fish species and families have been monitored by the Australian Institute of Marine Science's Long-Term Monitoring Program from 1993 to current. Only those species that were included in the statistical analyses are presented here, including family, scientific and common name. 'X' indicates species that have been reported to consume benthic life stages of Crown-of-Thorns Starfish (CoTS, *Acanthaster* spp.)[20]. CAAB code represents Codes for Australian Aquatic Biota (CAAB) number (https://www.cmar.csiro.au/data/caab/).

Scientific name	Common name	CoTS consumption	CAAB code
**Labridae (Wrasses)**			
Cheilinus fasciatus	Redbreast Maori Wrasse	X	37 384066
Cheilinus undulatus	Humphead Maori Wrasse	X	37 384038
Choerodon fasciatus	Harlequin Tuskfish		37 384073
Hemigymnus fasciatus	Fiveband Wrasse		37 384124
Hemigymnus melapterus	Thicklip Wrasse		37 384125
**Lethrinidae (Emperors)**			
Lethrinus atkinsoni	Yellowtail Emperor	X	37 351013
Lethrinus erythracanthus	Orangespotted Emperor		37 351025
Lethrinus erythropterus	Longfin Emperor		37 351029
Lethrinus harak	Thumbprint Emperor		37 351017
Lethrinus laticaudis	Grass Emperor	X	37 351006
Lethrinus lentjan	Redspot Emperor	X	37 351007
Lethrinus microdon	Smalltooth Emperor		37 351011
Lethrinus miniatus	Redthroat Emperor	X	37 351009
Lethrinus nebulosus	Spangled Emperor	X	37 351008
Lethrinus obsoletus	Orangestriped Emperor		37 351019
Lethrinus olivaceus	Longnose Emperor		37 351004
Lethrinus ornatus	Ornate Emperor	X	37 351015
Lethrinus rubrioperculatus	Spotcheek Emperor		37 351012
Lethrinus semicinctus	Blackblotch Emperor		37 351016
Lethrinus xanthochilus	Yellowlip Emperor		37 351020
Monotaxis grandoculis	Bigeye Seabream	X	37 351026
**Lutjanidae (Tropical Snappers)**			
Lutjanus adetii	Hussar		37 346033
Lutjanus argentimaculatus	Mangrove Jack		37 346015

Scientific name	Common name	CoTS consumption	CAAB code
Lutjanus biguttatus	Twospot Snapper		37 346039
Lutjanus bohar	Red Bass	X	37 346029
Lutjanus boutton	Yellowlined Snapper		37 346040
Lutjanus carponotatus	Stripey Snapper		37 346011
Lutjanus ehrenbergii	Ehrenberg's Snapper		37 346042
Lutjanus fulviflamma	Blackspot Snapper	X	37 346034
Lutjanus fulvus	Blacktail Snapper		37 346043
Lutjanus gibbus	Paddletail	X	37 346028
Lutjanus kasmira	Bluestriped Snapper		37 346044
Lutjanus lemniscatus	Darktail Snapper		37 346010
Lutjanus lutjanus	Bigeye Snapper		37 346008
Lutjanus monostigma	Onespot Snapper		37 346045
Lutjanus quinquelineatus	Fiveline Snapper		37 346006
Lutjanus rivulatus	Maori Snapper		37 346016
Lutjanus russellii	Moses' Snapper	X	37 346065
Lutjanus sebae	Red Emperor		37 346004
Lutjanus semicinctus	Blackbanded Snapper		37 346046
Lutjanus vitta	Brownstripe Snapper		37 346003
Macolor spp.	Midnight Snapper, Black-and-White Snapper		37 346047, 37 346048
**Serranidae (Rockcods)**			
Aethaloperca rogaa	Redmouth Rockcod		37 311134
Anyperodon leucogrammicus	Whitelined Rockcod		37 311085
Cephalopholis argus	Peacock Rockcod		37 311082
Cephalopholis boenak	Brownbarred Rockcod		37 311008
Cephalopholis cyanostigma	Bluespotted Rockcod		37 311136
Cephalopholis microprion	Dot-head Rockcod		37 311139
Cephalopholis miniata	Coral Rockcod		37 311083
Cephalopholis sexmaculata	Sixband Rockcod		37 311140
Cephalopholis urodeta	Flagtail Rockcod		37 311142
Cromileptes altivelis	Barramundi Cod		37 311044
Epinephelus areolatus	Yellowspotted Rockcod		37 311009
Epinephelus coeruleopunctatus	Whitespotted Grouper		37 311070
Epinephelus coioides	Goldspotted Rockcod		37 311007
Epinephelus corallicola	Coral Grouper		37 311066
Epinephelus cyanopodus	Purple Rockcod	X	37 311145
Epinephelus fasciatus	Blacktip Rockcod		37 311014
Epinephelus fuscoguttatus	Flowery Rockcod		37 311021
Epinephelus hexagonatus	Wirenet Rockcod		37 311064
Epinephelus howlandi	Blacksaddle Rockcod		37 311148
Epinephelus lanceolatus	Queensland Groper	X	37 311061
Epinephelus macrospilos	Snubnose Grouper		37 311149
Epinephelus maculatus	Highfin Grouper		37 311011
Epinephelus malabaricus	Blackspotted Rockcod		37 311150

Scientific name	Common name	CoTS consumption	CAAB code
Epinephelus merra	Birdwire Rockcod		37 311063
Epinephelus ongus	Specklefin Grouper		37 311069
Epinephelus polyphekadion	Camouflage Grouper		37 311047
Epinephelus quoyanus	Longfin Rockcod		37 311040
Epinephelus sexfasciatus	Sixbar Grouper		37 311017
Epinephelus spilotoceps	Foursaddle Grouper		37 311155
Plectropomus areolatus	Passionfruit Coral Trout		37 311081
Plectropomus laevis	Bluespotted Coral Trout		37 311079
Plectropomus leopardus	Common Coral Trout		37 311078
Plectropomus maculatus	Barcheek Coral Trout		37 311012
Variola albimarginata	White-edge Coronation Trout		37 311026
Variola louti	Yellowedge Coronation Trout		37 311166

Supplementary Table 6. Model estimates and hypothesis testing for coral reef fish biomass, density and length as a function of reef zoning status.

Parameter estimates were obtained by employing either gamma (for length) or hurdle-gamma (for density and biomass) models via a Bayesian approach. Models were fitted to each of the three response variables for six fish groups, namely (1) Labridae (wrasses), (2) Lethrinidae (emperors), (3) *Lethrinus miniatus* and *L. nebulosus* (redthroat and spangled emperors), (4) Lutjanidae (tropical snappers), (5) Serranidae (rockcods) and (6) *Plectropomus* spp. and *Variola* spp. (coral trout). Mean parameter estimates, as well as their 95% Bayesian credible intervals, are presented on the linear predictor scale (with the exception of Prob. 0; please see Supplementary Method 3 for model descriptions). The "Test" column comprises the actual parameter hypotheses tests following a priori expectations, i.e. whether a parameter is lower or greater than 0 or 1. The "Test probability" column comprises the exceedance probability, i.e. the proportion of a parameter's posterior distribution which supports the test. Response (U) (response on unfished reef on natural log scale) = β_0; Response (F) (response on fished reef on natural log scale) = $\beta_0 + \beta_1$; Delta Response (F - U) = β_1; Prob. 0 (U) (probability of response being zero on unfished reef) = $logit^{-1}(\theta_0)$; Prob. 0 (F) (probability of response being zero on fished reef) = $logit^{-1}(\theta_0 + \theta_1)$; Odds ratio prob. 0 (F / U) = Prob. 0 (F) / Prob. 0 (U). Fifteen of the 18 models provided more than 80% probability, and eleven of the 18 models more than 95% probability, that fish biomass, density and length were higher for five of the six fish groups on unfished reefs compared to fished reefs.

(1) Labridae (wrasses)

Response	Parameter	Estimate	Q2.5	Q97.5	Test	Test probability
Density	Response (U)	-2.62	-2.91	-2.33	-	-
	Response (F)	-2.65	-2.93	-2.36	-	-

Response	Parameter	Estimate	Q2.5	Q97.5	Test	Test probability
	Delta Response (F - U)	-0.03	-0.17	0.12	< 0	65.94
	Prob. 0 (U)	0.02	0.01	0.04	-	-
	Prob. 0 (F)	0.02	0.01	0.03	-	-
	Odds ratio prob. 0 (F / U)	0.86	0.37	1.68	> 1	28.6
Biomass	Response (U)	2.28	1.18	3.39	-	-
	Response (F)	2.11	0.99	3.22	-	-
	Delta Response (F - U)	-0.18	-0.53	0.18	< 0	83.58
	Prob. 0 (U)	0.71	0.67	0.74	-	-
	Prob. 0 (F)	0.71	0.68	0.75	-	-
	Odds ratio prob. 0 (F / U)	1.01	0.94	1.08	> 1	60.34
Length	Response (U)	-1.29	-1.47	-1.1	-	-
	Response (F)	-1.33	-1.53	-1.15	-	-
	Delta Response (F - U)	-0.05	-0.21	0.12	< 0	71.3

(2) Lethrinidae (emperors)

Response	Parameter	Estimate	Q2.5	Q97.5	Test	Test probability
Density	Response (U)	-3.89	-4.06	-3.71	-	-
	Response (F)	-4.05	-4.23	-3.86	-	-
	Delta Response (F - U)	-0.16	-0.37	0.05	< 0	93.38
	Prob. 0 (U)	0.38	0.34	0.42	-	-
	Prob. 0 (F)	0.49	0.46	0.53	-	-
	Odds ratio prob. 0 (F / U)	1.31	1.15	1.48	> 1	100
Biomass	Response (U)	0.97	0.71	1.22	-	-
	Response (F)	0.61	0.35	0.87	-	-
	Delta Response (F - U)	-0.36	-0.63	-0.1	< 0	99.56
	Prob. 0 (U)	0.39	0.35	0.42	-	-
	Prob. 0 (F)	0.5	0.46	0.53	-	-
	Odds ratio prob. 0 (F / U)	1.29	1.13	1.46	> 1	99.98
Length	Response (U)	-1.76	-1.94	-1.59	-	-
	Response (F)	-2.04	-2.21	-1.86	-	-
	Delta Response (F - U)	-0.27	-0.46	-0.08	< 0	99.76

(3) *Lethrinus miniatus* and *L. nebulosus* (redthroat and spangled emperors)

Response	Parameter	Estimate	Q2.5	Q97.5	Test	Test probability
Density	Response (U)	-4.08	-4.28	-3.88	-	-
	Response (F)	-4.18	-4.41	-3.96	-	-
	Delta Response (F - U)	-0.1	-0.34	0.14	< 0	80.36
	Prob. 0 (U)	0.61	0.57	0.65	-	-

Response	Parameter	Estimate	Q2.5	Q97.5	Test	Test probability
	Prob. 0 (F)	0.74	0.71	0.77	-	-
	Odds ratio prob. 0 (F / U)	1.21	1.12	1.31	> 1	100
Biomass	Response (U)	1.18	0.94	1.41	-	-
	Response (F)	0.95	0.7	1.21	-	-
	Delta Response (F - U)	-0.23	-0.5	0.05	< 0	95.08
	Prob. 0 (U)	0.61	0.57	0.65	-	-
	Prob. 0 (F)	0.74	0.71	0.78	-	-
	Odds ratio prob. 0 (F / U)	1.21	1.12	1.31	> 1	100
Length	Response (U)	-1.79	-1.96	-1.62	-	-
	Response (F)	-2.01	-2.19	-1.83	-	-
	Delta Response (F - U)	-0.22	-0.4	-0.03	< 0	98.52

(4) Lutjanidae (tropical snappers)

Response	Parameter	Estimate	Q2.5	Q97.5	Test	Test probability
Density	Response (U)	-3.06	-3.37	-2.75	-	-
	Response (F)	-3.18	-3.48	-2.87	-	-
	Delta Response (F - U)	-0.11	-0.43	0.21	< 0	76.64
	Prob. 0 (U)	0.21	0.18	0.24	-	-
	Prob. 0 (F)	0.21	0.18	0.25	-	-
	Odds ratio prob. 0 (F / U)	1.03	0.83	1.26	> 1	57.62
Biomass	Response (U)	1.15	0.82	1.47	-	-
	Response (F)	0.81	0.49	1.13	-	-
	Delta Response (F - U)	-0.34	-0.66	-0.01	< 0	97.82
	Prob. 0 (U)	0.21	0.18	0.24	-	-
	Prob. 0 (F)	0.21	0.18	0.24	-	-
	Odds ratio prob. 0 (F / U)	1.01	0.81	1.24	> 1	50.8
Length	Response (U)	-1.26	-1.57	-0.95	-	-
	Response (F)	-1.45	-1.76	-1.14	-	-
	Delta Response (F - U)	-0.19	-0.42	0.05	< 0	93.86

(5) Serranidae (rockcods)

Response	Parameter	Estimate	Q2.5	Q97.5	Test	Test probability
Density	Response (U)	-2.64	-2.89	-2.38	-	-
	Response (F)	-3.02	-3.28	-2.76	-	-
	Delta Response (F - U)	-0.38	-0.54	-0.22	< 0	100
	Prob. 0 (U)	0.03	0.02	0.04	-	-
	Prob. 0 (F)	0.03	0.02	0.04	-	-
	Odds ratio prob. 0 (F / U)	0.95	0.46	1.71	> 1	36.85

Response	Parameter	Estimate	Q2.5	Q97.5	Test	Test probability
Biomass	Response (U)	1.95	1.68	2.22	-	-
	Response (F)	1.26	0.99	1.53	-	-
	Delta Response (F - U)	-0.69	-0.89	-0.48	< 0	100
	Prob. 0 (U)	0.03	0.02	0.04	-	-
	Prob. 0 (F)	0.03	0.02	0.04	-	-
	Odds ratio prob. 0 (F / U)	0.95	0.46	1.73	> 1	36.95
Length	Response (U)	-0.49	-0.74	-0.23	-	-
	Response (F)	-0.95	-1.21	-0.7	-	-
	Delta Response (F - U)	-0.47	-0.64	-0.29	< 0	100

(6) *Plectropomus* spp. and *Variola* spp. (coral trout)

Response	Parameter	Estimate	Q2.5	Q97.5	Test	Test probability
Density	Response (U)	-2.99	-3.24	-2.74	-	-
	Response (F)	-3.46	-3.72	-3.21	-	-
	Delta Response (F - U)	-0.47	-0.67	-0.26	< 0	100
	Prob. 0 (U)	0.09	0.07	0.11	-	-
	Prob. 0 (F)	0.12	0.1	0.15	-	-
	Odds ratio prob. 0 (F / U)	1.42	1.01	1.95	> 1	97.81
Biomass	Response (U)	1.92	1.66	2.17	-	-
	Response (F)	1.18	0.93	1.44	-	-
	Delta Response (F - U)	-0.74	-0.96	-0.51	< 0	100
	Prob. 0 (U)	0.09	0.07	0.11	-	-
	Prob. 0 (F)	0.12	0.1	0.15	-	-
	Odds ratio prob. 0 (F / U)	1.42	1.01	1.96	> 1	97.96
Length	Response (U)	-0.69	-0.93	-0.45	-	-
	Response (F)	-1.22	-1.46	-0.99	-	-
	Delta Response (F - U)	-0.53	-0.74	-0.33	< 0	99.99

Supplementary Table 7. Average estimates of fish biomass removal (kg per site) trespassing the Crown-of-Thorns Starfish threshold towards potential outbreaks.

Estimates were obtained from the response of Pacific Crown-of-Thorns Starfish (CoTS, *Acanthaster* cf. *solaris*) density to removal of coral reef fish biomass (Supplementary Figs. 2 and 3) and are presented for those fish groups and time lags where one of the 95% credible intervals overlapped the 'No outbreak' threshold of an average of ≤0.1 CoTS per manta tow. CoTS population outbreak status information from the Great Barrier Reef Marine Park's Eye on the Reef program, following definitions in De'ath (2003)[40]. Note that 'No outbreak' status does not mean reefs are totally CoTS free. Other outbreak status thresholds are: >0.11 = Potential outbreak, >0.22 = Established outbreak, and >1.0 = Severe outbreak.

Fish group	Time lag (yrs)	Estimate	Q2.5	Q97.5	Estimate (kg)	Q2.5 (kg)	Q97.5 (kg)	Exceedance probability
Lethrinidae (Emperors)	1	1.969	3.803	NA	6.162	43.828	NA	0.999
	2	0.548	2.841	NA	0.730	16.131	NA	0.998
	4	NA	1.906	NA	NA	5.726	NA	0.968
	5	NA	2.517	NA	NA	11.394	NA	0.964
Lethrinus miniatus and *L. nebulosus* (Redthroat and Spangled emperors)	1	NA	2.368	NA	NA	9.675	NA	0.974
	2	NA	2.377	NA	NA	9.771	NA	0.990
	4	NA	1.812	NA	NA	5.121	NA	0.963
	6	NA	0.784	NA	NA	1.191	NA	0.861
Lutjanidae (Tropical snappers)	1	NA	2.888	NA	NA	16.965	NA	0.966

Fish group	Time lag (yrs)	Estimate	Q2.5	Q97.5	Estimate (kg)	Q2.5 (kg)	Q97.5 (kg)	Exceedance probability
	2	NA	1.101	NA	NA	2.008	NA	0.877
	3	NA	NA	NA	NA	NA	NA	0.874
	4	NA	NA	NA	NA	NA	NA	0.816
Serranidae (Rockcods)	1	NA	3.968	NA	NA	51.861	NA	0.910
	2	NA	3.192	NA	NA	23.344	NA	0.938
	4	1.709	4.204	NA	4.524	65.927	NA	0.996
Plectropomus spp. and *Variola* spp. (Coral trout)	1	NA	3.511	NA	NA	32.496	NA	0.846
	2	NA	3.245	NA	NA	24.657	NA	0.947
	4	2.930	4.690	NA	17.727	107.833	NA	0.999
	5	NA	3.988	NA	NA	52.949	NA	0.892
	6	NA	2.544	NA	NA	11.734	NA	0.825

Supplementary Table 8. Categories and estimates for coral cover monitored on the Great Barrier Reef Marine Park, Australia.

Coral cover categories are used during manta tow surveys conducted on the Great Barrier Reef Marine Park, Australia, as part of the Australian Institute of Marine Science's Long-Term Monitoring Program. The observer assesses categorical coral cover at the end of each two-minute tow[41].

Category	Coral cover estimate (%)
0	0%
1-	>0-5%
1+	>5-10%
2-	>10-20%
2+	>20-30%
3-	>30-40%
3+	>40-50%
4-	>50-62.5%
4+	>62.5-75%
5-	>75-87.5%
5+	>87.5-100%

Supplementary Table 9. Size, age estimate and life stage categories for Pacific Crown-of-Thorns Starfish (*Acanthaster* cf. *solaris*) monitored on the Great Barrier Reef Marine Park, Australia.

Crown-of-Thorns Starfish (CoTS) categories are used during manta tow surveys conducted on the Great Barrier Reef Marine Park, Australia, as part of the Australian Institute of Marine Science's Long-Term Monitoring Program. The observer assesses the number and size of any CoTS recorded at the end of each two-minute tow[41].

Size	Age estimate	Category
<= 5 cm	Up to one year	J (Early juvenile)
6 - 15 cm	One to two years	A (Juvenile)
15-25 cm	Two to three years	B (Sub-Adult)
>25cm	Three plus year	C (Adult)

Supplementary References

1. Great Barrier Reef Marine Park Authority. Great Barrier Reef Outlook Report 2019. 372. (Great Barrier Reef Marine Park Authority, Townsville, Australia, 2019).
2. Great Barrier Reef Marine Park Authority. Great Barrier Reef Outlook Report 2009. 212. (Great Barrier Reef Marine Park Authority, Townsville, Australia, 2009).
3. McCook, L. J. et al. Adaptive management of the Great Barrier Reef: A globally significant demonstration of the benefits of networks of marine reserves. *Proc. Natl. Acad. Sci. USA* **107**, 18278-18285 (2010).
4. Sweatman, H. No-take reserves protect coral reefs from predatory starfish. *Curr. Biol.* **18**, R598-R599 (2008).
5. Pratchett, M., Caballes, C. F., Rivera-Posada, J. A. & Sweatman, H. P. A. Limits to our understanding and managing outbreaks of Crown-of-Thorn Starfish (*Acanthaster* spp.). *Oceanogr. Mar. Biol. Annu. Rev.* **52**, 133-200 (2014).
6. Vanhatalo, J., Hosack, G. R. & Sweatman, H. Spatiotemporal modelling of crown-of-thorns starfish outbreaks on the Great Barrier Reef to inform control strategies. *J. Appl. Ecol.* **54**, 188-197 (2016).
7. Endean, R. Report on investigations made into aspects of the current *Acanthaster planci* (Crown of Thorns) infestations of certain reefs of the Great Barrier Reef. Queensland Department of Primary Industries (Fisheries Branch) 22 (Queensland Government, Brisbane, Australia, 1969).
8. Barnes, J. H. The crown-of-thorns starfish as a destroyer of coral. *Austral. Nat. Hist.* **15**, 257-261 (1966).
9. Endean, R. Crown-of-thorns starfish on the Great Barrier Reef. *Endeavour* **6**, 10-14 (1982).
10. Wachenfeld, D. R., Oliver, J. K. & Morrissey, J. I. State of the Great Barrier Reef World Heritage Area. 139 (Great Barrier Reef Marine Park Authority, Townsville, Australia, 1998).
11. Kenchington, R. A. Growth and recruitment of *Acanthaster planci* (L.) on the Great Barrier Reef. *Biol. Conserv.* **11**, 103-118 (1977).
12. Hock, K., Wolff, N. H., Condie, S. A., Anthony, K. R. N. & Mumby, P. J. Connectivity networks reveal the risks of crown-of-thorns starfish outbreaks on the Great Barrier Reef. *J. Appl. Ecol.* **51**, 1188-1196 (2014).
13. Reichelt, R. E., Bradbury, R. H. & Moran, P. J. Distribution of *Acanthaster planci* outbreaks on the Great Barrier Reef between 1966 and 1989. *Coral Reefs* **9**, 97-103 (1990).
14. Moran, P. J. et al. Pattern of outbreaks of crown-of-thorns starfish (*Acanthaster planci* L.) along the Great Barrier Reef since 1966. *Austral. J. Mar. Freshw. Res.* **43**, 555 (1992).
15. Uthicke, S., Doyle, J., Duggan, S., Yasuda, N. & McKinnon, A. D. Outbreak of coral-eating Crown-of-Thorns creates continuous cloud of larvae over 320 km of the Great Barrier Reef. *Sci. Rep.* **5**, 16885 (2015).

16. Babcock, R. C., Milton, D. A. & Pratchett, M. S. Relationships between size and reproductive output in the crown-of-thorns starfish. *Mar. Biol.* **163**, (2016).

17. Uthicke, S. et al. Spawning time of *Acanthaster cf. solaris* on the Great Barrier Reef inferred using qPCR quantification of embryos and larvae: do they know it's Christmas? *Mar. Biol.* **166**, 1-10 (2019).

18. Deaker, D. J. et al. The hidden army: corallivorous crown-of-thorns seastars can spend years as herbivorous juveniles. *Biol. Lett.* **16**, 20190849 (2020).

19. Cowan, Z. L., Pratchett, M. S., Messmer, V. & Ling, S. Known predators of crown-of-thorns starfish (*Acanthaster* spp.) and their role in mitigating, if not preventing, population outbreaks. *Diversity* **9**, 7 (2017).

20. Kroon, F. J. et al. DNA-based identification of predators of the corallivorous Crown-of-Thorns Starfish (*Acanthaster* cf. *solaris*) from fish faeces and gut contents. *Sci. Rep.* **10**, 8184 (2020).

21. Department of Agriculture and Fisheries. Queensland Fisheries Summary. 62 (Queensland Government, Brisbane, Australia, 2018).

22. Department of Agriculture and Fisheries. Sustainable Fisheries Strategy 2017-2027. Coral Reef Fin Fish Fishery (line Fishery Reef). Status report for reassessment and approval under protected species and export provisions of the Environment Protection and Biodiversity Conservation Act 1999. 29 (Queensland Government, Brisbane, Australia, 2020).

23. Steven, A. An analysis of fishing activities on possible predators of crown of thorn starfish (*Acanthaster planci*) on the Great Barrier Reef. Unpublished report to GBRMPA. (Project 329: 1986-88). 131 (Great Barrier Reef Marine Park Authority, Townsville, Australia, 1988).

24. Daley, B. Changes in the Great Barrier Reef since European settlement: implications for contemporary management. PhD thesis. (James Cook University, Townsville, Australia, 2005).

25. Department of the Environment and Energy. Assessment of the Queensland Coral Reef Fin Fish Fishery. 39 (Australian Government, Canberra, Australia, 2017).

26. Department of Agriculture and Fisheries. Queensland Fisheries Summary Report. Catch and Effort Data for Queensland's Commercial Fisheries. Updated Data for the 2018/19 Financial Year. 31 (Queensland Government, Brisbane, Australia, 2020).

27. Webley, J., McInnes, K., Teixeira, D., Lawson, A. & Quinn, R. Statewide recreational fishing survey 2013–14. Department of Agriculture and Fisheries. 145 (Queensland Government, Brisbane, Australia, 2015).

28. Williams, D. M. & Russ, G. R. Review of data on fishes of commercial and recreational fishing interest in the Great Barrier Reef. 113 (Great Barrier Reef Marine Park Authority, Townsville, Australia, 1984).

29. Henry, G. W. & Lyle, J. M. The national recreational and indigenous fishing survey. Department of Agriculture, Fisheries and Forestry. 190 (Australian Government, Canberra, Australia, 2003).

30. Taylor, S., Webley, J. & McInnes, K. 2010 Statewide recreational fishing survey. Department of Agriculture, Fisheries and Forestry. 93 (Queensland Government, Brisbane, Australia, 2012).

31. Teixeira D, Janes R, Webley J. 2019/20 Statewide recreational fishing survey key results. Project Report. State of Queensland. 18 (Queensland Government, Brisbane, Australia, 2021).

32. Department of the Environment and Heritage. Assessment of the Queensland marine aquarium fish fishery. 25 (Australian Government, Canberra, Australia, 2005).

33. Department of Primary Industries and Fisheries. A guide to the Queensland Marine Aquarium Fish Fishery and the Queensland Coral Fishery. Department of Employment, Economic Development and Innovation. 16 (Queensland Government, Brisbane, Australia, 2009).

34. Ryan, S. & Clarke, K. Ecological assessment of the Queensland marine aquarium fish fishery. A report to the Australian Government Department of Environment and Heritage on the ecologically sustainable management of the Queensland marine aquarium harvest fishery. 78 (Queenland Government, Brisbane, Australia, 2005).

35. Bürkner, P. C. brms: An R Package for Bayesian multilevel models using Stan. *J. Stat. Softw.* **80**, 1-28 (2017).

36. Gelman, A. & Rubin, D. B. Inference from iterative simulation using multiple sequences. *Stat. Sci.* **7**, 457–472 (1992).

37. Gelman, A., Goodrich, B., Gabry, J. & Vehtari, A. R-squared for Bayesian regression models. *Am. Stat.* **73**, 307–309 (2019).

38. Hartig, F. DHARMa: Residual Diagnostics for Hierarchical (Multi-Level / Mixed) Regression Models. R package version 0.3.3.0. https://CRAN.R-project.org/package=DHARMa. (2020).

39. Cowan, Z. L., Ling, S. D., Caballes, C. F., Dworjanyn, S. A. & Pratchett, M. S. Crown-of-thorns starfish larvae are vulnerable to predation even in the presence of alternative prey. *Coral Reefs* **39**, 293–303 (2020).

40. De'ath, G. Analyses of crown-of-thorns starfish data from the fine-scale surveys and the long-term monitoring program manta tow surveys. 75 (CRC Reef Research Centre Ltd, Townsville, Australia, 2003).

41. Miller, I. R., Jonker, M. J. & Coleman, G. Crown-of-thorns starfish and coral surveys using the manta tow technique. 43 (Australian Institute of Marine Science, Townsville, Australia, 2019).
